# Immunoglobulin Superfamily Protein BTNL9 Functions as a Non-Canonical Transcriptional Regulator to Suppress NSCLC Through Cell Cycle and p53 Pathways

**DOI:** 10.3390/ijms27156598

**Published:** 2026-07-24

**Authors:** Wooi Loon Ng, Pedram Yadollahi, Hwa Jin Cho, Mi Seon Kang, Inhak Choi

**Affiliations:** 1 Innovative Therapeutics Research Institute, Inje University, Busan 47397, Republic of Korea; 2 Department of Microbiology and Immunology, Inje University College of Medicine, Busan 47397, Republic of Korea; 3 Department of Pathology, Busan Paik Hospital, Inje University, Busan 47397, Republic of Korea; eunhasu101@hanmail.net (H.J.C.); pathmsk@hanmail.net (M.S.K.)

**Keywords:** BTNL9, lung cancer, novel transcription regulator, tumor suppressor genes, chemosensitivity

## Abstract

Butyrophilin-like 9 (BTNL9), a member of the immunoglobulin superfamily containing a bZIP-like domain, has a poorly defined role in cancer. Here, we identify BTNL9 as a non-canonical transcriptional regulator and investigate its function in non-small cell lung cancer (NSCLC). Coiled-coil prediction, native PAGE, and co-immunoprecipitation demonstrated BTNL9 homodimerization via its bZIP-like region, while subcellular fractionation and immunofluorescence confirmed its presence in both the nucleus and cytoplasm. Chromatin immunoprecipitation sequencing (ChIP-seq) analysis identified 9709 BTNL9-associated genomic regions, including sites proximal to transcription start sites, with enrichment of a cytosine-rich motif. Whether chromatin association reflects direct DNA binding or indirect co-regulatory interaction remains to be experimentally confirmed. Integrated transcriptomic and protein analyses revealed that BTNL9 overexpression represses genes involved in cell cycle progression and DNA replication while activating a subset of p53-associated pathways. Consistently, functional assays showed that increased BTNL9 expression induces cell cycle arrest, suppresses proliferation and clonogenicity, and inhibits tumor growth in xenograft models. In addition, cytotoxicity assays demonstrated enhanced sensitivity to bortezomib, with context-dependent effects on etoposide response. Analysis of public clinical datasets further showed that low BTNL9 expression is associated with advanced tumor stage, reduced remission rates, and poorer survival outcomes in NSCLC. These findings identify BTNL9 as a non-canonical tumor-suppressive transcriptional regulator with potential biomarker relevance in NSCLC.

## 1. Introduction

Butyrophilins (BTNs) constitute a group of transmembrane proteins that belong to the immunoglobulin superfamily and are structurally homologous with immune-regulatory B7 family proteins. BTNs participate in modulating immune responses via costimulatory or inhibitory signals and have been linked to autoimmune inflammatory diseases and lipid metabolism [1,2,3,4,5]. The human genome harbors 13 BTN/BTN-like (BTNL) genes [6], among which BTNL9 has attracted attention, owing to its distinctive structural and functional features. BTNL9 has been demonstrated to function as a negative regulator of T cell activation in Jurkat T cells and murine models [7,8]. The IgV1 domain of BTNL9 has the capacity to interact with and bind to CD8 T cells, B cells, and NK cells, thereby suppressing immune responses [9]. Most BTN/BTN-like genes possess distinct IgV, IgC, and SPRY/B30.2 domains. While BTNL9 presents with one IgV domain, it lacks a clear IgC domain, unlike the highly structurally similar BTNL3 and BTNL8. Moreover, the absence of two critical cysteine residues at positions 173 and 227, which are crucial for the stabilization of the IgC domain through disulfide bonding, indicates that BTNL9 might not have a typical immunoglobulin structure [6]. Another unique feature of the protein sequence of BTNL9 is that it exhibits a basic leucine zipper domain (bZIP domain), a common feature among transcription factors, but has just a single leucine heptad repeat that encompasses a coiled-coil region (current data in this study). In addition to these unique structural features of BTNL9, recent in vitro studies have revealed that it has inhibitory effects on cell proliferation, invasion, and migration in melanoma and breast cancer cell lines [10,11]. Further, transcriptomics data mining has suggested a link between higher BTNL9 expression and good prognosis of lung, breast, bone, colon, colorectal, pancreatic and thyroid cancer, as well as indicating its potential role in tumor suppression [12,13,14,15,16,17,18,19,20,21,22,23].

In this study, we provide evidence supporting that BTNL9 acts as a non-canonical transcriptional regulator in non-small cell lung cancer (NSCLC). It includes a global mapping of BTNL9’s binding sites and its active involvement in regulating genes critical for DNA replication and the cell cycle. Furthermore, BTNL9 appears to steer genes tied to p53-regulated transcription in a p53-dependent manner. Using a xenograft model, we showed that BTNL9 inhibits tumor growth in vivo. We also demonstrated that BTNL9 protein level in cancer cells is associated with etoposide and bortezomib treatment sensitivity. Analysis of clinical data from NSCLC patients revealed that a high level of BTNL9 expression was associated with improved survival outcomes and higher rates of complete remission in those undergoing primary treatment. Overall, our study offers a deeper understanding of transcriptional regulation and tumor suppression dynamics, hinting at a broader mechanism for cancer control.

## 2. Results

### 2.1. Characterization of Protein Structure and Subcellular Localization of BTNL9

**Figure 1:**
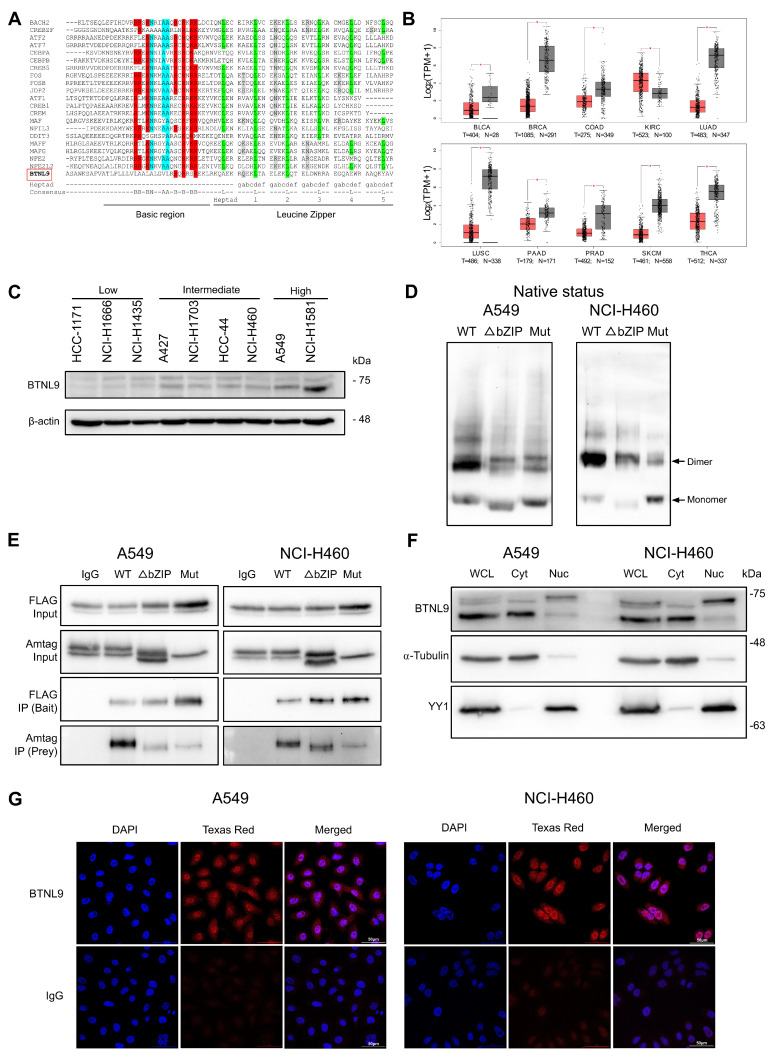
Characterization of protein structure and subcellular localization of BTNL9. (**A**) Alignment analysis of bZIP transcription factor genes shows the conserved leucine zipper region, with BTNL9 corresponding to a single heptad repeat. Key annotations: conserved leucine (L) residues are highlighted in green, polar or charged amino acids in gray, basic amino acids in red, and conserved amino acids in the basic region in aqua blue. Consensus sequence is shown at the bottom of the figure, with B representing either arginine (R) or lysine (K). Heptad repeats and their positions are denoted as 1–5 and “abcdefg”, respectively. (**B**) Boxplots depict the expression levels of BTNL9 (log_2_ TPM + 1) in tumor (T, red) and adjacent normal (N, gray) tissues across multiple cancer types, including BLCA (Bladder Urothelial Carcinoma), BRCA (Breast invasive carcinoma), COAD (Colon adenocarcinoma), KIRC (Kidney renal clear cell carcinoma), LUAD (Lung adenocarcinoma), LUSC (Lung squamous cell carcinoma), PAAD (Pancreatic adenocarcinoma), PRAD (Prostate adenocarcinoma), SKCM (Skin Cutaneous Melanoma) and THCA (Thyroid carcinoma). The number of tumor (T) and normal (N) samples analyzed for each cancer type is indicated below each boxplot. Asterisks (*) indicate statistically significant differences in BTNL9 expression between tumor and normal tissues (* *p* < 0.05). These data were derived from the TCGA dataset, and the figure was generated using GEPIA2. (**C**) Western blot analysis of the protein levels of BTNL9 across different lung cancer cell lines. (**D**) Native PAGE reveals distinct dimeric and monomeric forms of BTNL9 under non denaturing conditions. Wild-type BTNL9 shows prominent dimer bands, whereas ΔbZIP and mutant constructs display altered migration patterns consistent with impaired dimerization capacity. Results were independently reproduced in both A549 and NCI H460 cell lines. (**E**) AM-tagged BTNL9 constructs (WT, ΔbZIP, Mut) were co-expressed with FLAG-tagged BTNL9 and subjected to FLAG immunoprecipitation. Western blot detection of co-immunoprecipitated AM-tagged BTNL9 indicates that deletion or mutation of the bZIP-like domain disrupts BTNL9 self-association. IgG immunoprecipitation serves as a negative control. (**F**) Subcellular fractionation of A549 and NCI-H460 cells followed by SDS-PAGE (8% gel) and Western blot shows BTNL9 proteins in both cytoplasmic and nuclear fractions. Anti-⍺-tubulin and anti-YY1 were used to confirm the accurate partitioning of cytoplasmic and nuclear fractions, respectively. WCL: whole cell lysate, Cyt: cytoplasm, Nuc: nucleus. (**G**) Subcellular localization of BTNL9 in A549 and NCI-H460 cells was assessed by immunofluorescence. Cells were stained with rabbit anti-BTNL9 primary antibody detected with Texas Red-conjugated secondary antibody (red) or rabbit IgG as isotype control. Nuclei were counterstained with DAPI (blue). Images were acquired using a confocal microscope with a 40× objective lens, scale bar = 50 µm. All confocal images presented are unmodified and uncropped from the original acquisition.

Based on the annotated reference protein sequence deposited in the NCBI database (NP_689760.2), BTNL9 contains a basic leucine zipper (bZIP) domain spanning residues 281 and 317. Notably, BTNL9 contains a single leucine heptad repeat that deviates from the typical bZIP pattern of transcription factors, which usually possess four to five heptad repeats. The “a” and “d” positions in the heptad repeat are important for dimerization stability and are typically occupied by hydrophobic amino acids, a feature not observed in the BTNL9 protein sequence (Figure 1A). Moreover, unlike other members of the bZIP transcription factor family, BTNL9 shows reduced similarity in its basic region compared to other transcription factors. These observations indicate that BTNL9 does not align with the typical characteristics of the bZIP transcription factor family and may instead belong to a distinct class of transcriptional regulators.

To investigate whether BTNL9 possesses structural features consistent with transcriptional regulators, we performed conserved domain analysis using the NCBI CD-search (Figure S1A). BTNL9 exhibited three high-confidence domains: an IgV-MOG-like domain (E = 3.4 × 10^−60^), a C-terminal SPRY/PRY domain (E = 3.9 × 10^−89^), and importantly, a significant match to the bZIP_GCN4 family (residues 281–317; E = 3.5 × 10^−3^). Although this region lacks some canonical residues typically found in classical bZIP proteins, the E-value falls within the confidence range for true positive matches, indicating non-random homology and supporting its potential for DNA interaction or dimerization. Coiled-coil prediction analyses consistently identified a single, well defined coiled-coil region within BTNL9 (Figure S1B,C). Using Marcoil, a strong coiled-coil probability peak was detected spanning amino acids 274–315, and this finding was independently supported by Multicoil2, which similarly predicted a high confidence coiled-coil segment in the same region. Together, these results indicate that BTNL9 contains one robust coiled-coil domain, suggesting a structural basis for its potential protein–protein interaction capabilities.

Prior studies have suggested a tumor-suppressive role for BTNL9 in several cancer types, and the structural features described in this study raise the possibility that this activity may be mediated through its transcriptional regulation. To explore this further, we examined BTNL9 mRNA levels in tumor and normal tissues using the TCGA dataset, revealing consistent downregulation in tumors spanning multiple cancer types (Figure 1B). Among these, breast invasive carcinoma, lung adenocarcinoma (LUAD), and lung squamous cell carcinoma (LUSC) exhibited the most pronounced reductions. NSCLC was selected for further investigation because it combines relatively high baseline BTNL9 expression in normal tissue with consistent and marked downregulation in both independent subtypes, providing a favorable dynamic range for mechanistic analysis. We subsequently profiled BTNL9 protein expression in a panel of human lung cancer cell lines (Figure 1C). Based on these data, A549 (LUAD) and NCI-H460 (large cell lung carcinoma, LCLC) were selected for further study due to their relatively rapid doubling times, intermediate BTNL9 expression levels relative to the broader panel, and wild-type TP53 status, providing a defined genetic context while permitting exploration of potential p53-related regulatory effects in subsequent analyses. Although NCI-H1703 and NCI-H1581 are derived from LUSC tumors, both harbor TP53 mutations, which may introduce confounding effects in the interpretation of p53-associated pathways. In addition, NCI-H1581 exhibits semi-suspension growth, limiting its suitability for adherent-based phenotypic assays.

To investigate the role of the bZIP domain in BTNL9 dimerization, we performed native Polyacrylamide Gel Electrophoresis (PAGE) and co-immunoprecipitation (co-IP) assays in A549 and NCI-H460 cells. In addition to the wild-type construct, ΔbZIP and bZIP-mutant constructs were generated to determine whether this putative domain contributes to BTNL9 dimerization (Figure S1D). The bZIP-mutant construct was generated by introducing multiple helix-disrupting proline substitutions into conserved residues of the bZIP domain to impair α-helical coiled-coil formation and thereby disrupt BTNL9 dimerization. Native PAGE analysis revealed the presence of a dimeric BTNL9 species in wild-type constructs, whereas ΔbZIP and bZIP-mutant constructs showed altered migration patterns indicative of a reduced dimerization capacity, most notably in the bZIP-mutant construct (Figure 1D). Because native PAGE does not permit strict quantitative comparison of protein abundance, these data were interpreted qualitatively. Consistently, co-IP using FLAG-tagged full-length BTNL9 as bait demonstrated robust self-association of wild-type BTNL9, while both ΔbZIP and mutant constructs showed markedly reduced interaction in both cell lines (Figure 1E). Together, these results indicate that the bZIP domain is required for efficient BTNL9 homodimerization and complex stability.

We next evaluated BTNL9 subcellular localization through protein fractionation and Western blot analysis (Figure 1F). Three BTNL9 bands were detected below 75 kDa in whole cell lysates of both A549 and NCI-H460 cells, consistent with an estimated molecular weight of approximately 60 kDa for the full-length protein. Notably, the highest molecular weight band was predominantly enriched in the nuclear fraction, while the two smaller bands were largely confined to the cytoplasm. To explore the basis for nuclear localization, we used NLStradamus to predict NLS sequences within the BTNL9 protein. A putative NLS spanning residues 278–294 (RKQRRSREKLRKQAEKR) was identified with a prediction score exceeding the 0.6 confidence threshold (Figure S1E). Alignment of multiple BTNL9 transcript variants confirmed that this NLS is conserved across variants V1, V2 and X1-X7, whereas variants X8 and X9 lack this region due to sequence truncation and correspondingly show markedly lower predicted molecular weights (Figure S1F). Based on molecular weight comparisons and NLS predictions, the three observed bands may potentially correspond to NLS-containing variants, although direct isoform identification through mass spectrometry or isoform-specific approaches will be required to confirm this inference. The isoform-specific distribution observed between nuclear and cytoplasmic compartments suggests additional regulatory mechanisms governing BTNL9 localization that merit future investigation. To visually validate the nuclear presence of BTNL9, we conducted confocal microscopy on A549 and NCI-H460 cells. Consistent with the protein fractionation results, BTNL9 was observed in both the cytoplasmic and nuclear compartments, with pronounced nuclear localization in both cell lines (Figure 1G). This pattern of distribution is consistent with the localization characteristics commonly observed for chromatin-associated regulatory proteins.

### 2.2. Insight of BTNL9 Genome-Wide Binding Sites

**Figure 2:**
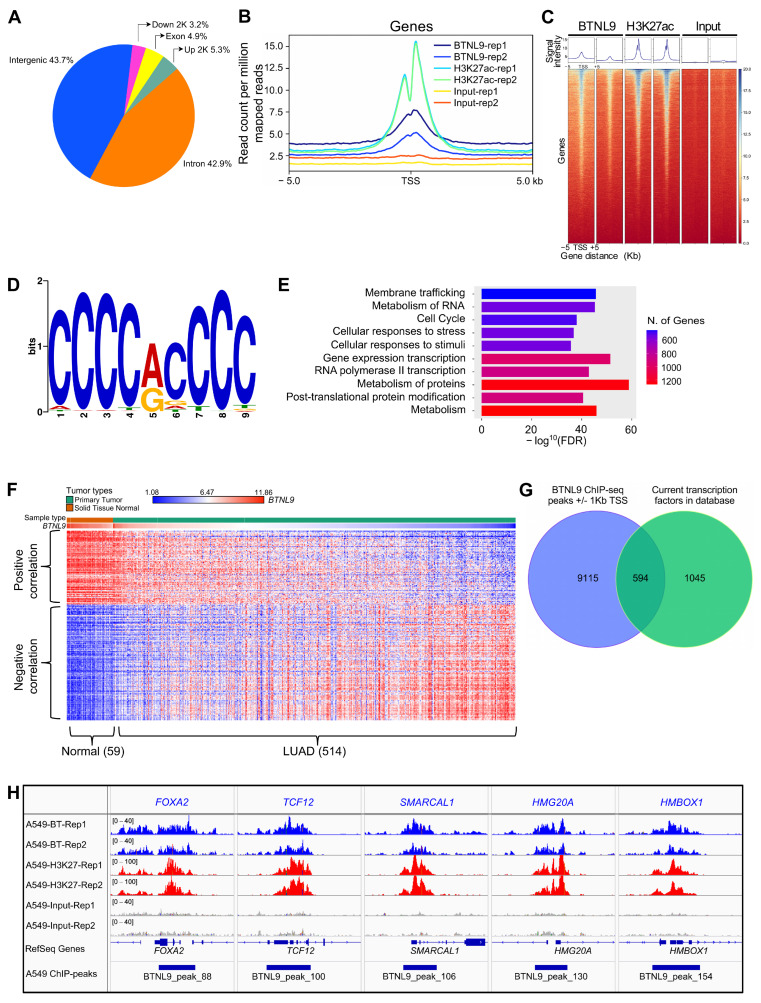
Insight of BTNL9 genome-wide binding sites. (**A**) Distribution of BTNL9 binding sites based on peak location across different genomic regions of the human genome. (**B**) Density plot of read counts showing the relative ChIP-seq read density near the transcriptional start site (TSS) of BTNL9, H3K27ac and ChIP input within ±5 Kb. (**C**) Heat map illustrating the quantity of genes bound by BTNL9, H3K27ac and ChIP input control around TSS. (**D**) De novo motif of the potential conserved DNA binding motif of BTNL9 was generated by using MEME-ChIP motif analysis. (**E**) Bar graph representing Reactome pathway analysis of genes identified from ChIP-seq differential peak calling. The top 10 significant pathways were shown. (**F**) Heatmap representing expression profiles of genes significantly correlated to BTNL9 mRNA levels from TCGA LUAD dataset (Spearman’s correlation coefficient *r* ≤ −0.45 or *r* ≥ 0.45). (**G**) The Venn diagram illustrates candidate transcription factors identified from BTNL9 ChIP-seq peaks, suggesting potential involvement of BTNL9 in their transcriptional regulation. (**H**) Integrative Genomic Viewer (IGV) plot displays ChIP-seq peak of BTNL9, H3K27ac, and input control at the loci of the top 5 transcription factors in the list.

In order to investigate the genome-wide chromatin association of BTNL9, we performed chromatin immunoprecipitation sequencing (ChIP-seq) in A549 cells. Due to the low endogenous BTNL9 protein level in A549 cells, BTNL9-AM-tag was overexpressed and chromatin immunoprecipitation was performed using an anti-AM-tag antibody. The ChIP-seq data revealed a wide distribution of genome binding sites: while 10% of the sites were detected in the upstream promoter and exon regions, the majority (~87%) were located within intergenic and intronic regions (Figure 2A). A similar genomic distribution pattern has been reported for other transcription factors [24,25]. The density plot of read counts and heatmap analysis indicated that BTNL9 bound to transcription start site (TSS) regions, where these genes were actively transcribed (with strong peaks observed for H3K27ac) (Figure 2B,C). This provides initial evidence of the ability of BTNL9 to bind to promoter regions. Utilizing MEME-ChIP motif analysis, we deciphered the potential conserved DNA binding motif of BTNL9 (Figure 2D and Figure S2A). The conserved sequence CCCCRCCCC was derived from the central 200 bp position of BTNL9 ChIP-seq peak replicate data.

Through ChIP-seq peak calling using MACS2 on pooled biological replicates against pooled input controls, we initially filtered peaks based on *p* < 1.0 × 10^−5^ and a Benjamini–Hochberg false discovery rate (FDR) of *q* < 0.005, yielding a total of 26,610 high-confidence BTNL9 associated peaks selected for further analysis (Table S1). To evaluate inter-replicate reproducibility, a genome-wide peak overlap analysis was performed using independently filtered peak sets from replicate 1 and replicate 2 (restricted to standard chromosomes chr1-22, X, and Y). The majority of peaks identified in replicate 2 (86.1%) overlapped with peaks detected in replicate 1, supporting strong reproducibility between biological replicates and validating the pooled peak calling approach (Figure S2B,C). We then used the Genomic Regions Enrichment of Annotations Tool (GREAT) to annotate ChIP peaks to the nearest genes within ±1 kb of the TSS, identifying 9709 BTNL9 associated genomic regions proximal to annotated gene loci (Table S2). To validate these findings, we selected five representative genes involved in cell cycle, DNA replication, and p53-regulated transcription (*TP53*, *CDKN1A*, E2F1, *MCM7* and *BBC3*) based on ChIP-seq enrichment and functional relevance (Table S3) and performed ChIP-quantitative polymerase chain reaction (ChIP-qPCR) in A549 cells overexpressing BTNL9-AM-tag (Figure S2D). Using fold-enrichment analysis, we observed significant enrichment of BTNL9 ChIP-DNA at these loci.

We analyzed these 9709 genes using the ShinyGo V0.82 tool to assess Reactome pathway enrichment, identifying the top 10 enriched functional pathways: membrane trafficking, metabolism of RNA, cell cycle, cellular responses to stress and stimuli, gene expression transcription, RNA polymerase II transcription, metabolism of protein, post-translational protein modification and metabolism (Figure 2E). The results of this analysis imply that BTNL9 binds to genes critical for gene regulation and cell stress responses. To investigate the potential transcriptional regulation of BTNL9 in NSCLC, we performed Spearman’s correlation analysis between BTNL9 and these 9709 genes RNA sequencing (RNA-seq) data in lung adenocarcinoma (LUAD) and lung squamous cell carcinoma (LUSC) patients. Using cBioPortal’s TCGA database, we identified 249 genes with substantial clinical relevance to BTNL9; among them, 92 genes were positively correlated and 157 were negatively correlated, based on Spearman’s correlation coefficient cutoffs of *r* ≤ −0.45 or *r* ≥ 0.45 (Table S4). Heatmaps were generated to visualize the correlation patterns in LUAD (59 normal, 514 tumor samples) and LUSC (51 normal, 502 tumor samples) (Figure 2F and Figure S2E).

In normal tissues adjacent to LUAD, genes formed distinct clusters of positive and negative correlations, suggesting that BTNL9 may have a differential regulatory role. In LUAD tumor samples, the correlation patterns followed a gradient based on BTNL9 expression levels, indicating that these genes may be regulated in a BTNL9-dependent manner. In contrast, LUSC samples exhibited a more asymmetric correlation pattern. While positively correlated genes aligned with BTNL9 expression levels, negatively correlated genes in LUSC did not show a clear relationship with BTNL9, suggesting a weaker or more indirect regulatory influence. This contrast between LUAD and LUSC implies that BTNL9 may have distinct transcriptional roles in different lung cancer subtypes.

We next examined whether BTNL9 specifically binds to the promoter or intronic regions of any potential transcription factors. To this end, we compared 9709 genes from our ChIP-seq data with an updated list of 1639 transcription factors from the AnimalTFDB V4 database. Through this analysis, we identified 594 transcription factors that BTNL9 binds to (Figure 2G and Table S5). We highlighted five transcription factors (*FOXA2*, *TCF12*, *SMARCAL1*, *HMG20A* and *HMBOX1*), where BTNL9 binding sites significantly overlapped with H3K27ac peaks, indicative of active transcription regions (Figure 2H). These findings strongly imply that BTNL9 binds to a substantial number of genes either at the promoter or in intronic regions, highlighting its potential role as a novel transcription regulator in a multilayered hierarchical gene regulatory network.

### 2.3. Analysis of BTNL9 RNA-Seq Data Obtained in A549 Cell Line

**Figure 3:**
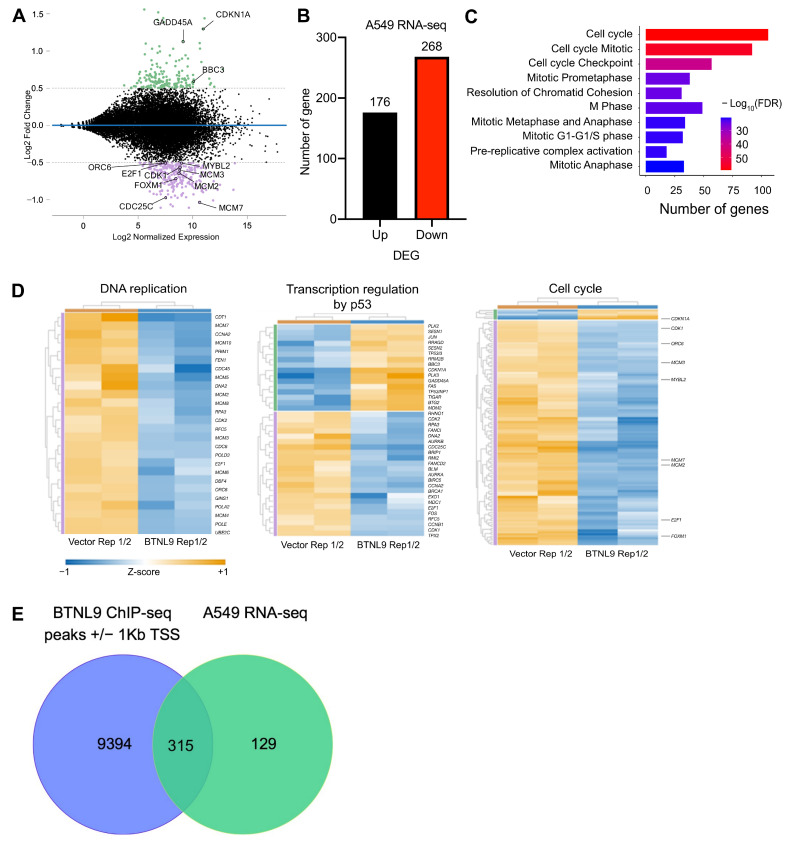
Regulatory role of BTNL9 in cell cycle, DNA replication, and p53-regulated genes in A549 LUAD cell line. (**A**) MA plot displaying the distribution of DEGs from A549 RNA-seq dataset, with upregulated genes shown in green and downregulated genes in purple. This analysis is based on the cut-off value of log_2_ FC ± 0.5 and a false discovery rate (FDR) of ≤0.1. Genes marked with labels were further analyzed in subsequent experiments. (**B**) Bar graph showing the number of DEGs that met the criteria of log_2_ FC ± 0.5 and FDR < 0.1. (**C**) Bar graph representing the top 10 significant pathways from the Reactome pathway analysis of statistically significant genes identified from the RNA-seq. (**D**) Heatmap generated using the PAM (Partitioning Around Medoids) method with the fpc R library, showcasing 3 major DEG clusters associated with DNA replication (26 genes), transcription regulation by p53 (36 genes) and cell cycle (119 genes). (**E**) Venn diagram showing the overlap between two sets of genes identified through both ChIP-seq and RNA-seq analyses in A549 cells.

We investigated the role of BTNL9 in A549 cells by using lentivirus to induce overexpression of BTNL9 with an empty vector or a BTNL9-overexpression (BTNL9-OE) vector. Based on preliminary time-course experiments, BTNL9 expression reached maximal levels on Day 3 following transduction and gradually declined thereafter (Figure S3A). Therefore, total RNA was harvested on Day 3 for RNA-seq analysis to capture the peak transcriptional response associated with BTNL9 overexpression. Duplicate RNA samples from each experimental group were submitted to E-biogen (South Korea) for RNA-seq analysis. For exploratory transcriptomic analysis, we identified 444 candidate differentially expressed genes (DEGs) using predefined discovery thresholds of log_2_ FC ± 0.5 and FDR *q* < 0.1. The MA plot revealed an even distribution of DEGs, with 176 genes identified as upregulated and 268 genes as downregulated (Figure 3A,B, Table S6). To elucidate the functions of BTNL9, we employed the ShinyGo V0.82 tool for DEG analysis. Reactome pathway enrichment analysis of the candidate DEGs identified several pathways potentially associated with BTNL9 expression, with the most significantly enriched pathways related to cell cycle regulation and cell division (Figure 3C). Additionally, we generated a heatmap with hierarchical clustering of genes influenced by BTNL9 expression and detected three clusters that were associated with DNA replication, transcription regulation mediated by p53, and cell cycle (Figure 3D).

Within the candidate DNA replication-associated gene cluster identified from the RNA-seq dataset, BTNL9 overexpression was associated with downregulation of the entire minichromosome maintenance protein (MCM) family gene set, including MCM2–MCM8 and MCM10 (Table S7). The MCM complex acts as a DNA helicase and is a crucial component of the DNA replication licensing system [26]. Moreover, BTNL9 overexpression was associated with altered expression of multiple additional genes involved in DNA replication, including the licensing factor CDT1, genes involved in G2/M transition (CCNA2, CDK2, and UBE2C), polymerase catalytic subunits (POLE and POLA2), and the DNA primase PRIM1, which synthesizes RNA primers required for Okazaki fragment formation.

We also identified a cluster of 36 candidate DEGs associated with p53-mediated transcriptional regulation (Table S7). These genes modulate various aspects of cellular activity such as cell cycle checkpoint, DNA repair, DNA replication, and programmed cell death. Critical effectors in this cluster include *BBC3*, *CDC25C*, *CDK1*, *CDKN1A*, *E2F1*, *GADD45A* and *MDM2*. This observation suggested a potential association between BTNL9 and p53-related transcriptional programs, a hypothesis that was subsequently investigated through targeted validation experiments and is consistent with a potential role for BTNL9 in maintaining genome integrity.

Our analysis identified a candidate cell-cycle-associated cluster comprising 119 genes whose expression was altered following BTNL9 overexpression (Table S7). These genes include common cell cycle regulators such as cyclins and CDKs, cell division cycle genes, and genes encoding centromere-associated proteins such as *CENPA* and *CENPE* (Table S7). Inactivation of these genes is known to lead to mitotic arrest; moreover, CENPA plays an important role in kinetochore recruitment for chromatid segregation [27,28,29]. Furthermore, genes encoding condensins, which are structural maintenance of chromosome (SMC) proteins, and non-SMC condensins were also identified within the cluster, crucial for maintaining sister chromatid structure during mitosis and meiosis [30].

We compared the 444 candidate DEGs with the 9709 BTNL9-associated genes identified by ChIP-seq and found 315 overlapping genes containing the conserved CCCCRCCCC motif within their promoter regions (Figure 3E and Figure S3B). The substantial overlap between transcriptomic and chromatin occupancy datasets suggests a potential association between BTNL9-responsive transcriptional programs and BTNL9-associated chromatin regions, while the recurrent CCCCRCCCC motif may reflect a sequence preference within these loci. (Table S8). To further evaluate BTNL9 association with target gene promoters, we performed a DNA-protein pulldown assay using a 237 bp biotinylated fragment of the E2F1 promoter that encompasses multiple BTNL9 putative binding sites. BTNL9 was strongly enriched in the pulldown fraction, whereas non-DNA-binding controls (p21 and β-actin) were not detected (Figure S3C), demonstrating the specificity of the assay and supporting promoter engagement by BTNL9. Due to the presence of multiple, closely spaced putative binding sites, individual motif-mutated probes were not evaluated. Nevertheless, the combined evidence from pulldown enrichment, the presence of a ChIP-seq peak at this locus, and subsequent ChIP-qPCR validation provides convergent evidence supporting an association between BTNL9 and promoter DNA regions, consistent with a role in transcriptional regulation.

### 2.4. Regulatory Role of BTNL9 in Cell Cycle Progression Through Multiple Pathways

**Figure 4:**
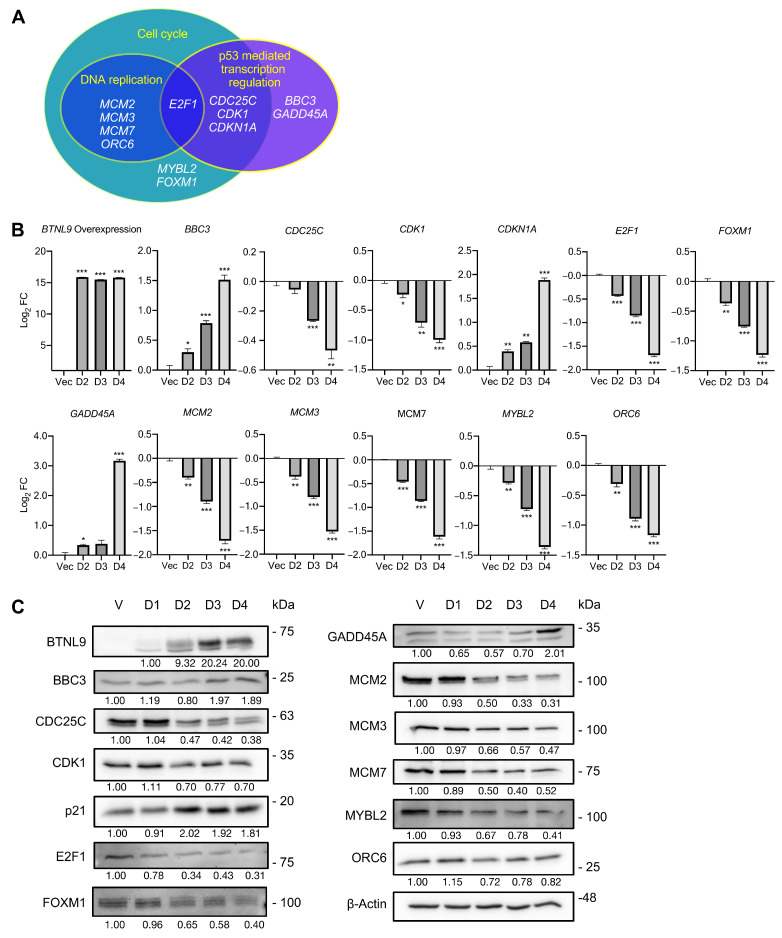
Validation of RNA-seq data using qRT-PCR and Western blot. (**A**) Venn diagram illustrating the overlap between the functional pathways of the selected genes. (**B**) Bar graphs representing qRT-PCR analyses in A549 cells overexpressing either empty vector control or BTNL9, with samples harvested on the indicated day. All values were calculated as mean log_2_ FC ± SD. Significance was determined by a two-tailed Student’s *t*-test: * *p* < 0.05, ** *p* < 0.01 and *** *p* < 0.001. All experiments were conducted in triplicates. Primers for qRT-PCR of tested genes were listed in Table S9. (**C**) Representative Western blot of selected genes in A549 cells overexpressing either empty vector control or BTNL9, with samples harvested on the indicated day. β-actin was used as a loading control. Signal intensities were quantified with iBright Analysis software and semi-quantitative protein expression levels were calculated by normalizing to β-actin.

To gain a deeper understanding of BTNL9’s functions in cancer cells, especially in DNA replication, cell cycle and p53 mediated regulation, we selected several well-characterized genes that could elucidate its role in suppressing tumor activities. We focused on 12 genes from the three clusters identified in our RNA-seq data, as their potential to inhibit tumor growth was suggested by their overlapping functions (Figure 4A). STRING network analysis confirmed functional connectivity among these genes (Figure S4A). Furthermore, our ChIP-seq data revealed potential BTNL9 binding at the TSS or intronic regions of some of these genes, suggesting possible regulatory interactions. From our ChIP-seq analysis, we determined that 8 genes, *BBC3*, *CDC25C*, *CDK1*, *FOXM1*, *GADD45A*, *MCM3*, *MCM7* and *ORC6*, exhibited significant ChIP-seq peaks (Figure S4B and Table S2). This suggests that BTNL9 acts as an upstream transcription regulator, either as an enhancer or repressor, regulating the expression of these genes. Subsequently, we validated the RNA expression profiles of all 12 genes using a two-step reverse transcription real-time PCR (RT-qPCR) assay in both A549 and NCI-H460 cell lines, specifically from Day 2 to Day 4 following the infection with BTNL9 lentivirus. Consistent with the RNA-seq data, we noted significant changes in gene expression on Day 3, which became more pronounced on Day 4 in both cell lines (Figure 4B and Figure S4C). Among these genes, *BBC3*, *CDKN1A* and *GADD45A* were significantly upregulated, whereas *CDC25C*, *CDK1*, *E2F1*, *FOXM1*, *MCM2*, *MCM3*, *MCM7*, *MYBL2*, and *ORC6* showed consistent downregulation in both cell lines.

To determine the protein levels of these genes, we harvested protein lysates from cells infected with either BTNL9- or vector-lentivirus at specified time points and performed a Western blot for the same genes that were previously examined using RT-qPCR. In both cell lines, the trends in the protein expression of most genes were consistent with those observed at the mRNA level (Figure 4C and Figure S4D). This result suggests that the expression levels of BTNL9 were sufficient to drive the transcription and translation of its downstream targets. Subsequently, we aimed to clarify whether p53 was involved in the regulation of BTNL9-regulated DEGs, particularly those within the p53-regulated gene cluster. To elucidate the relationship between BTNL9 and p53, we generated p53 knockout (KO) cell lines with the clustered regularly interspaced short palindromic repeats (CRISPR)-cas9 technique in both A549 and NCI-H460 cells. Western blot analysis confirmed the successful knockout, as no p53 protein expression was detected in p53 KO cells, even in the presence of Nutlin-3, a known MDM2 inhibitor (Figure S4E). To investigate the p53-dependent and p53-independent regulatory effects of BTNL9, RT-qPCR was performed to analyze the expression of several BTNL9 downstream target genes (*BBC3*, *CDC25C*, *CDK1*, *CDKN1A*, and *E2F1*) previously shown to be associated with p53 regulation (Figure S4F). In wild-type (WT) cells overexpressing BTNL9, *BBC3* and *CDKN1A* expression were markedly upregulated, while *CDC25C*, *CDK1*, and *E2F1* were downregulated, consistent with p53-mediated regulation. However, in p53 KO cells, BTNL9 overexpression did not significantly alter the expression of these 5 genes. To support the qPCR findings, we performed Western blot analysis on one representative BTNL9-regulated gene to assess protein level changes (Figure S4G). Overexpression of BTNL9 in A549 and H460 cells resulted in the upregulation of p21 protein expression, consistent with transcriptional induction. Critically, this induction was abolished in p53 KO cells, confirming that the BTNL9-mediated regulation of p21 is strictly p53-dependent. Although only one target was examined at the protein level, the result aligns with mRNA data and reinforces the functional relevance of p53 in BTNL9-driven transcriptional control.

### 2.5. Suppressive Effect of BTNL9 on Cell Proliferation, Clonogenicity, and Tumorigenicity in NSCLC Cells

**Figure 5:**
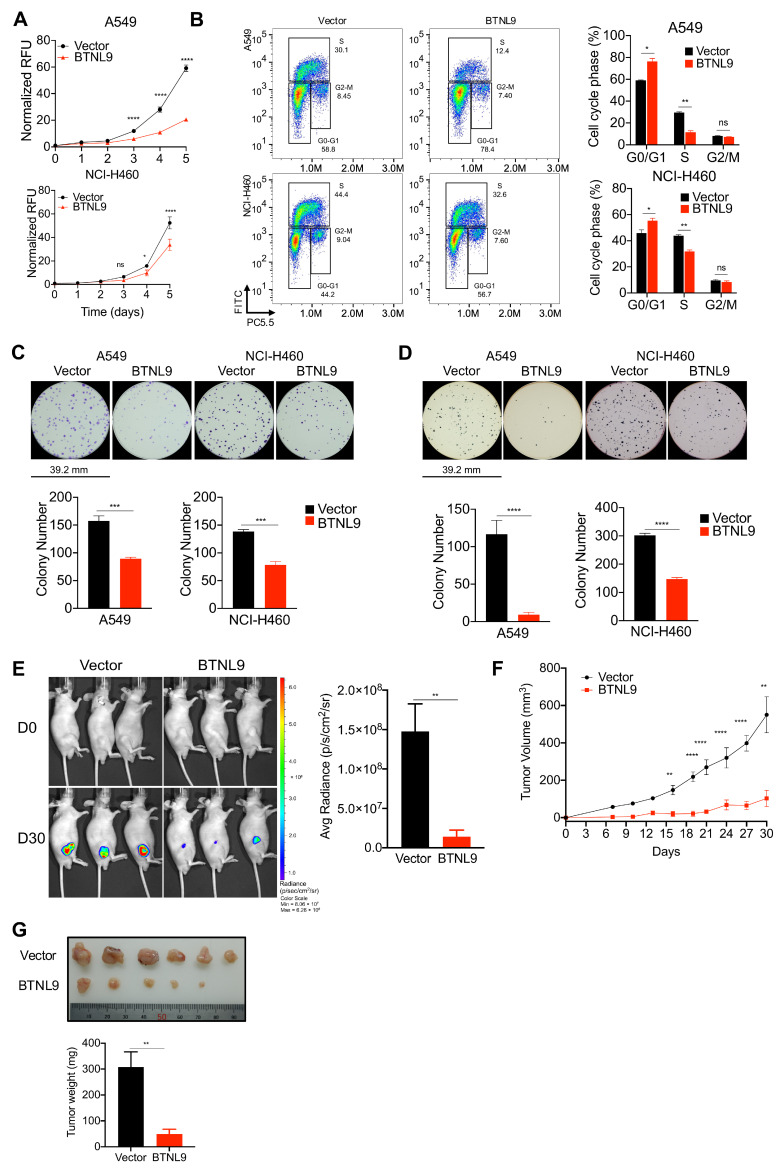
Suppressive effect of BTNL9 on cell proliferation and tumorigenicity in NSCLC cells. (**A**) A549 and NCI-H460 cells, overexpressing either empty vector control or BTNL9, were used in the following assays. All experiments were conducted in biological replicates. Cell proliferation of both cells was evaluated using resazurin to measure cell growth over 5 consecutive days. Growth curves were plotted as mean relative fluorescence units (RFU) ±  SD. (**B**) Representative scatter plots for the cell cycle assay, performed using BrdU staining on both cells, with isotype control serving as a negative control. Cell populations representing G0/G1 phase, S-phase and G2/M phase were gated. Bar graphs representing the compilation of individual experimental results with mean ± SD. (**C**) A clonogenic assay was conducted to investigate survival differences between an empty vector control and BTNL9 overexpression groups. The number of colonies was counted and the bar graph was plotted as the mean ±  SD. (**D**) Representative images of colonies from anchorage-independent growth assay between the empty vector control and BTNL9 overexpression groups. The number of colonies was counted and plotted in bar graphs as mean ±  SD. (**E**) BALB/c nude mice bearing subcutaneous A549 tumors in each group were imaged by IVIS on day 0 and day 30. The average radiance at the tumor site was normalized to the day 0 signal, and data were displayed in bar graphs as mean ± SEM (n = 6; only 3 mice in each group are shown). *p* values were calculated using a two-tailed Student’s *t*-test, ** *p*  <  0.01. (**F**) Tumor volumes in each group were measured every 3 days and the tumor growth curve was plotted as mean ± SEM (n = 6). A significant difference in growth rate was determined using a two-tailed Student’s *t*-test (** *p* <  0.01 and **** *p* < 0.0001). (**G**) Ex vivo image of the solid tumors of each group was photographed and the weights were plotted in a bar graph as mean ± SEM (n = 6; ** *p* < 0.01; unpaired two tail *t*-test). In vivo experiments were repeated twice. For (**A**,**B**) data represents the mean of triplicates and is tested with two-way ANOVA using the Tukey test. * *p* < 0.05, ** *p* < 0.01, **** *p* < 0.0001, and ns denotes no statistical differences. For (**C**,**D**) data represents the mean of triplicates and is tested by Student’s *t*-test (unpaired two-tailed). *** *p*  <  0.001, and **** *p* < 0.0001.

Having demonstrated that BTNL9 regulates the expression of a group of genes involved in cell cycle progression, we aimed to investigate cellular phenotypes, such as cell proliferation and clonogenicity, to further support BTNL9’s regulatory role in cancer cells. Through the cell proliferation assay using A549 and NCI-H460 cell lines, we observed a significant inhibition of cell proliferation on Day 5 in BTNL9-OE cells compared to the empty vector-expressing control cells in both cell lines (Figure 5A). To gain further insights into the mechanisms underlying this decline in proliferative activity, we conducted BrdU labeling assays to investigate whether this effect was cell cycle-dependent or associated with cell death. Our cell cycle analysis revealed dramatic cell cycle arrest at the G0/G1 phase, along with reduced replication activity during the S-phase (Figure 5B). Additionally, our clonogenic assay showed a decrease in colony formation in BTNL9-OE cells compared to the empty vector-expressing control cells (Figure 5C). To further investigate the impact of BTNL9 on tumorigenicity, we conducted an anchorage-independent assay, which revealed a drastic reduction in colony formation in BTNL9-OE cells (Figure 5D). Overall, our experimental results provide compelling evidence for the regulatory role of BTNL9 in suppressing both proliferative activity and tumorigenicity in NSCLC cell lines.

To investigate BTNL9’s tumor-suppressive effect under in vivo conditions, we used a xenograft mouse model by subcutaneously implanting A549 cells transduced with either BTNL9- or vector-lentivirus, and employed bioluminescence imaging to quantify tumor size. Images captured by IVIS on Day 30 revealed significantly larger tumors in the group implanted with A549 cells expressing the empty vector control cells than in the group implanted with BTNL9-OE cells (Figure 5E). Tumor volumes were measured every 3 days until Day 30, with notable differences between the groups becoming evident from Day 18 onward (Figure 5F and Figure S5A). Both visual and weight-based assessments confirmed marked tumor growth inhibition in the group implanted with cells overexpressing BTNL9 (Figure 5G). These findings indicate that BTNL9 acts as a tumor suppressor protein. In vivo Western blot analysis of A549 xenograft tumors revealed that BTNL9 overexpression reduced the protein levels of key cell cycle regulators, including FOXM1, Cyclin A2 (CCNA2), and Cyclin B1 (CCNB1), compared with vector control tumors (Figure S5B). FOXM1 regulates transcriptional targets across G1/S and G2/M, while CCNA2 and CCNB1 are essential for G2/M progression. Their suppression in vivo mirrors the transcriptional repression observed in vitro, reinforcing BTNL9’s role in cell cycle control. Together, these findings provide functional evidence that BTNL9 modulates cell cycle progression and contributes to the suppression of tumor growth.

### 2.6. Higher BTNL9 Expression Level Enhances Drug Sensitivity in NSCLC Cell Lines

To examine whether BTNL9 protein levels influence treatment response, we selected etoposide and bortezomib as representative agents based on their distinct mechanisms of action. Etoposide, a topoisomerase II inhibitor, induces DNA damage through activation of the DNA damage response [31], whereas bortezomib, a 26S proteasome inhibitor, disrupts proteasome-mediated protein degradation, including the turnover of key cell cycle regulators [32]. These pathways are functionally linked to the BTNL9-regulated transcriptional network involved in p53-mediated regulation and cell cycle control, as identified in our transcriptomic and ChIP-based analyses. Using cytotoxicity assays, cell cycle analyses, and downstream marker expression profiling, we aimed to elucidate how BTNL9 influences the efficacy of etoposide and bortezomib treatments.

**Figure 6:**
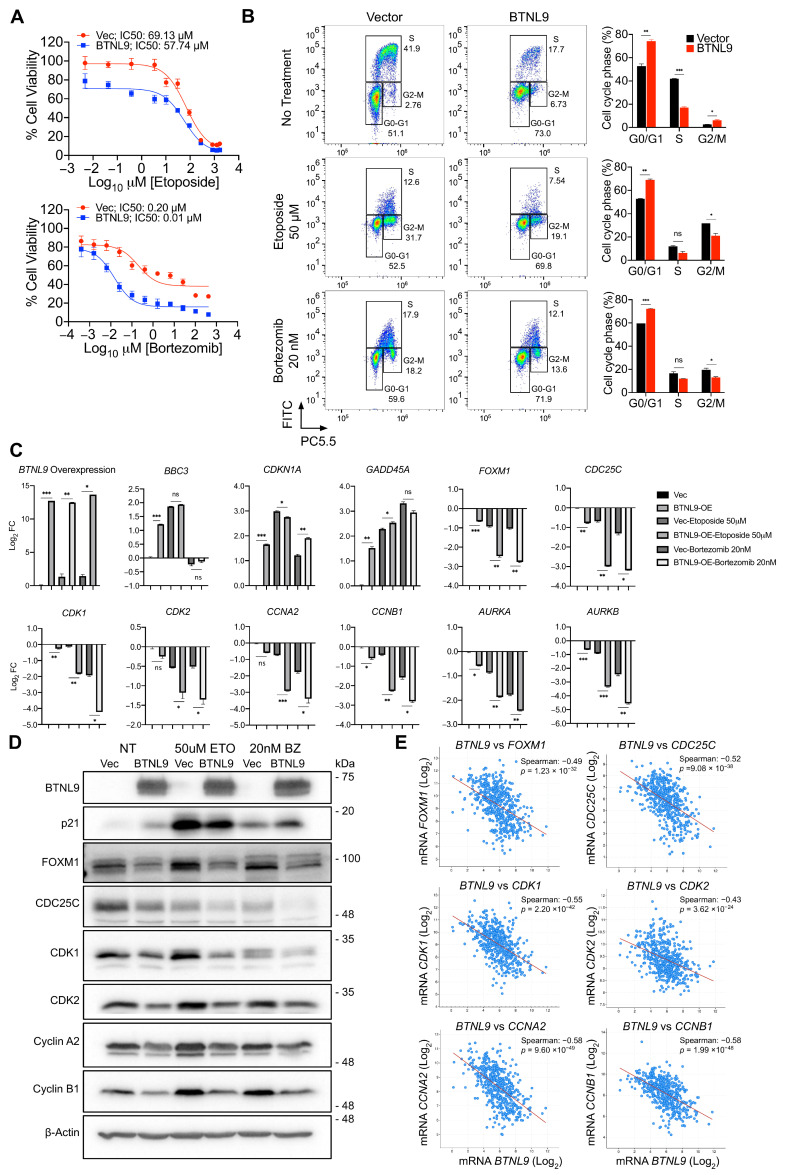
BTNL9 enhances drug sensitivity to etoposide and bortezomib in A549 cells by modulating cell cycle pathways. (**A**) Dose–response curves from cytotoxicity assays of etoposide and bortezomib treatments for 48 h in A549 cells overexpressing BTNL9 or vector control were used to estimate the IC50 value. (**B**) Representative scatter plots for the cell cycle assay, performed using BrdU-staining on vector control and BTNL9-overexpressing in A549 cells. Cell populations representing G0/G1 phase, S-phase and G2/M phase were gated. Bar graphs representing the compilation of individual experimental results with mean ± SD. (**C**) Bar graphs representing RT-qPCR analyses in A549 cells overexpressing either empty vector control or BTNL9. All values were calculated as mean log_2_ FC ± SD. All experiments were conducted in biological triplicates. Primers for qRT-PCR of tested genes were listed in Table S8. (**D**) Representative Western blot of selected key cell cycle markers in A549 cells overexpressing either empty vector control or BTNL9. β-actin was used as a loading control. (**E**) Correlation analysis between *BTNL9* mRNA levels and the expression of cell cycle-related genes (*FOXM1*, *CDC25C*, *CDK1*, *CDK2*, *CCNA2*, and *CCNB1*) in LUAD patient samples derived from TCGA dataset. Spearman correlation coefficients and *p*-values are shown, indicating a negative correlation between BTNL9 expression and these cell cycle-promoting genes. For (**B**–**D**), cells were either with no treatment (NT), or treated with 50 µM etoposide or 20 nM bortezomib for 18 h. For (**B**,**C**), data represents the mean of biological triplicates and is tested by Student’s *t*-test (unpaired two-tailed). * *p* < 0.05, ** *p* < 0.01, *** *p* < 0.001 and ns denotes no statistical differences.

To evaluate the role of BTNL9 in drug sensitivity, cytotoxicity assays were performed on A549 and NCI-H460 cells with either vector control or BTNL9 overexpression. BTNL9 overexpression significantly enhanced drug sensitivity in A549 and NCI-H460 NSCLC cell lines. In cells overexpressing BTNL9, the IC50 values for etoposide and bortezomib were reduced compared to vector control cells. Specifically, for etoposide, the IC50 decreased from 69.13 μM in vector cells to 57.74 μM in BTNL9-overexpressing A549 cells. Similarly, for bortezomib, the IC50 dropped markedly from 0.20 μM in vector cells to 0.01 μM in BTNL9-overexpressing A549 cells (Figure 6A). A similar trend was observed in NCI-H460 cells. When treated with etoposide, the IC50 showed a slight reduction from 0.14 μM in vector cells to 0.06 μM in BTNL9-overexpressing cells. In contrast, bortezomib treatment led to a substantial decrease in IC50, dropping from 9.30 μM in vector cells to 0.27 μM in BTNL9-overexpressing cells (Figure S6A).

BrdU cell cycle assays showed that BTNL9 overexpression significantly altered cell cycle distribution in A549 and NCI-H460 cells, particularly under drug treatment. Under the non-treatment condition, BTNL9-overexpressing cells displayed increased G0/G1 arrest and reduced S-phase entry compared with vector control, indicating suppressed proliferation. BTNL9 overexpression attenuated cell cycle progression following treatment with etoposide (50 μM) or bortezomib (20 nM). In NCI-H460 cells, drug exposure further increased G0/G1 accumulation, whereas A549 cells under BTNL9 overexpression maintained a consistently high G0/G1 fraction regardless of treatment status. In both cell lines, BTNL9 overexpression was associated with a significant reduction in S-phase cells and a concomitant decrease in the G2/M population, consistent with restricted cell cycle progression.

Collectively, these findings indicate that higher BTNL9 expression establishes a dominant growth-restrictive state that limits S-phase entry and constrains progression through later cell cycle phases, which is further reinforced under therapeutic stress. This enforced cell cycle restraint likely contributes to the enhanced chemosensitivity observed in NSCLC cells (Figure 6B and Figure S6B).

RT-qPCR analysis of both A549 and NCI-H460 cells revealed that BTNL9 overexpression led to the downregulation of key cell cycle-related genes, including FOXM1, CDC25C, CDK1, CDK2, CCNA2, CCNB1, AURKA and AURKB, with suppression further enhanced under drug treatment conditions. In contrast, BBC3, CDKN1A and GADD45A did not produce strong modulations compared to those key cell cycle-related genes (Figure 6C and Figure S6C). Western blot analysis confirmed these findings at the protein level. BTNL9-overexpressing cells in A549 and NCI-H460 treated with 50 μM etoposide or 20 nM bortezomib for 18 h exhibited reduced protein levels of FOXM1, CDC25C, CDK1, CDK2, Cyclin A2 (CCNA2), and Cyclin B1 (CCNB1). In contrast, p21 expression was upregulated following treatment with both drugs, with a more striking difference observed between the vector control and BTNL9 overexpression groups, particularly in response to bortezomib (Figure 6D and Figure S6D).

Using RNA-seq data from the TCGA dataset, the correlations between BTNL9 expression and cell cycle-related genes in LUAD and LUSC were analyzed. From the LUAD patient dataset, Spearman’s correlation analysis revealed a significant inverse relationship between BTNL9 mRNA expression and key cell cycle-related genes. BTNL9 showed strong negative correlations with FOXM1 (Spearman: −0.49, *p* = 1.23 × 10^−32^), CDC25C (−0.52, *p* = 9.08 × 10^−38^), CDK1 (−0.55, *p* = 2.20 × 10^−42^), CDK2 (−0.43, *p* = 3.62 × 10^−24^), CCNA2 (−0.58, *p* = 9.60 × 10^−49^), and CCNB1 (−0.58, *p* = 1.99 × 10^−48^) (Figure 6E). These correlations suggest that high BTNL9 expression is associated with the suppression of genes involved in cell cycle progression, particularly those required for S-phase and G2/M transition. From the LUSC patient dataset, the negative correlations were weaker but remained statistically significant for most genes (Figure S6E). For FOXM1, the correlation was weaker (Spearman: −0.10, *p* = 0.0232), and for CDC25C, no significant correlation was observed (Spearman: −0.07, *p* = 0.124). However, negative correlations were significant for CDK1 (−0.21, *p* = 2.01 × 10^−6^), CDK2 (−0.10, *p* = 0.0266), CCNA2 (−0.24, *p* = 6.18 × 10^−8^), and CCNB1 (−0.23, *p* = 1.56 × 10^−7^). This suggests that the relationship between BTNL9 and cell cycle-related genes is less pronounced in LUSC compared to LUAD.

Together, these findings demonstrate that a higher BTNL9 expression level enhances the cytotoxic effects of etoposide and bortezomib in NSCLC cells, lowering IC50 values and reinforcing drug-induced cell cycle arrest. By suppressing S-phase entry and downregulating key G2/M regulators, BTNL9 establishes a chemosensitive state that aligns with the transcriptomic correlations observed in LUAD patient datasets.

### 2.7. Multifaceted Impact of BTNL9 on the Progression and Prognosis of NSCLC

**Figure 7:**
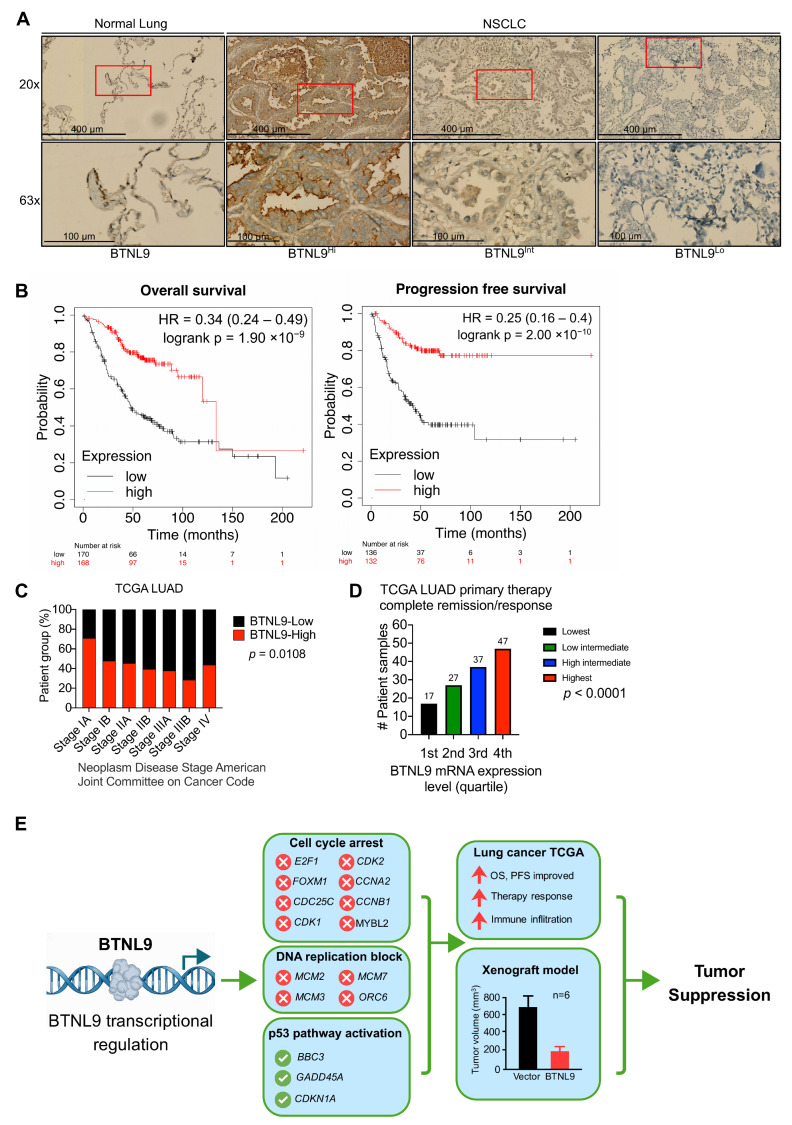
Multifaceted impact of BTNL9 on the progression and prognosis of NSCLC. (**A**) IHC staining of BTNL9 in selected LUAD tumor samples and normal lung tissue from a tissue microarray. Normal lung tissue is shown as a morphological and cellular localization reference for BTNL9 expression in non-malignant lung epithelium. Representative images are displayed at 20× and 63× magnification. The red box indicates an area of higher magnification. The BTNL9 stainings in tumor samples were categorized into high (BTNL9Hi), intermediate (BTNL9Int), and low (BTNL9Lo) expression, based on intensity. (**B**) Kaplan–Meier curve analysis comparing overall survival and progression-free survival in LUAD patients within the first and fourth quartiles of BTNL9 expression. The *p*-value was obtained from the log-rank test. (**C**) Bar graph showing the distribution of LUAD patients from lowest (1st quartile) to highest (4th quartile) BTNL9 mRNA expression level according to tumor stage (American Joint Committee on Cancer, AJCC). Data were generated using the TCGA LUAD dataset. The statistical association between BTNL9 expression and AJCC tumor stage was assessed by a Chi-squared test (χ^2^ = 16.62, df = 6, *p* = 0.0108) and confirmed by Spearman rank correlation trend test (rho = −0.786, *p* = 0.0362). (**D**) Bar graph depicting the number of LUAD patients with complete remission/response following primary treatment, distributed across the four quartiles of BTNL9 expression. Data were generated using the TCGA LUAD dataset. Statistical significance of the progressive trend across quartiles was assessed by Cochran–Armitage trend test (Z = 4.457, *p* < 0.0001). (**E**) Illustration of BTNL9’s function as a transcription regulator with tumor suppressive activity. BTNL9 regulates the transcription of genes involved in cell cycle arrest, DNA replication block, and p53 target gene activation, leading to tumor suppression in lung cancer. Higher BTNL9 expression levels are positively correlated with overall survival, progression-free survival, treatment outcomes, and immune infiltration. The xenograft mouse model also provides the solid evidence of BTNL9’s tumor suppressive activity.

To assess the clinical relevance of BTNL9’s tumor-suppressive role, as observed in our in vitro and in vivo studies, we first performed immunohistochemistry (IHC) staining on paraffin-embedded tissues from normal and lung cancer patients. IHC staining revealed the presence of BTNL9 in the NSCLC cells of LUAD samples, with its expression exhibiting varying intensities—high, intermediate, and low (Figure 7A). These findings are consistent with TCGA RNA-seq data, which show a broad spectrum of BTNL9 mRNA expression levels (Figure 1B). Additionally, high-magnification images of normal lung tissues clearly demonstrated that BTNL9, expressed in pneumocytes, is localized in the nucleus (Figure S7A).

Using the Kaplan–Meier plotter database, we analyzed data from 672 LUAD cases and 527 LUSC cases to generate Kaplan–Meier curves [33]. We divided BTNL9 expression levels into four quartiles for detailed comparison. Comparisons of survival rates between the lowest (1st quartile, BTNL9-Low) and highest (4th quartile, BTNL9-High) levels of BTNL9 demonstrated that LUAD patients with higher BTNL9 expression showed improvements in both overall survival and progression-free survival (Figure 7B). Conversely, no significant correlation between survival rates and BTNL9 expression level was observed for LUSC (Figure S7B). To further validate these associations and account for potential confounding factors, we performed multivariate Cox regression analysis incorporating clinical covariates including age, gender, tumor stage, and smoking history (Figure S7C). In LUAD, BTNL9 remained a significant independent predictor of both overall survival (HR = 0.58, 95% CI: 0.36–0.93, *p* = 0.023) and disease-free survival (HR = 0.50, 95% CI: 0.32–0.78, *p* = 0.002), with hazard ratios remaining significant after adjustment for clinical covariates. In contrast, BTNL9 did not reach statistical significance in LUSC for either overall survival (HR = 1.44, *p* = 0.078) or disease-free survival (HR = 1.59, *p* = 0.075), indicating that BTNL9 serves as an independent prognostic factor in LUAD but not in LUSC.

In addition, we further examined the overall survival of patients with various other cancer types. Our findings revealed that patients with breast cancer, liver cancer, clear cell renal cell carcinoma, and pancreatic ductal adenocarcinoma with higher BTNL9 expression also experienced significantly better overall survival rates. This suggests that higher BTNL9 expression not only benefits lung cancer patients but also those with a range of other cancers (Figure S7D).

Next, we selected TCGA data for LUAD and LUSC from the cBioPortal database for further analysis. For LUAD, patients were grouped based on BTNL9-Low and BTNL9-High expression levels and then sorted according to tumor stage (Figure 7C). BTNL9-High expression was significantly enriched in earlier tumor stages, particularly Stage IA, whereas BTNL9-Low expression was more frequently observed in advanced stages from Stage IIB onward (Chi-squared test: χ^2^ = 16.62, df = 6, *p* = 0.0108; Spearman trend test: rho = −0.786, *p* = 0.0362). In contrast, no significant association between BTNL9 expression and tumor stage was observed in LUSC (Chi-squared test: *p* = 0.217; Cochran–Armitage trend test: *p* = 0.452; Figure S7E), consistent with the subtype-specific prognostic relevance of BTNL9 across NSCLC histological subtypes. These findings suggest that BTNL9 expression is associated with tumor stage progression in a subtype-dependent manner in NSCLC.

We further investigated whether BTNL9 expression levels influence treatment outcomes. In LUAD, patients with higher BTNL9 expression showed a progressive increase in complete remission rates across expression quartiles (Cochran–Armitage trend test: Z = 4.457, *p* < 0.0001; Figure 7D), suggesting a potential association between BTNL9 expression and favorable therapeutic response. A similar trend was observed in LUSC, though with a smaller magnitude (Cochran–Armitage trend test: Z = 2.539, *p* = 0.011; Figure S7F). Collectively, these findings highlight the potential of BTNL9 as a prognostic marker in predicting therapeutic outcomes in NSCLC, particularly in LUAD.

Finally, we investigated the potential roles of BTNL9 in immune cell infiltration into the tumor immune microenvironment (TIME) using TISIDB, a web portal for tumor and immune system interaction. TCGA analysis uncovered a positive correlation between BTNL9 expression and the presence of infiltrated immune cells, including follicular helper T cells (Tfh), activated B cells, macrophages, effector CD8+ T cells, and natural killer (NK) cells, in both the LUAD and LUSC samples (Figure S7G). Interestingly, this correlation appeared slightly more pronounced in the LUSC samples than in the LUAD samples. These findings suggest that BTNL9 may play a role in tumor immunity during the tumor progression. Overall, these analyses imply that BTNL9 impacts the progression and clinical outcome of NSCLC, even though its effect appears to vary between LUSC and LUAD.

## 3. Discussion

In this study, our data support a potential role for BTNL9 as a transcriptional regulator with tumor-suppressive activity in NSCLC. Importantly, the term “transcriptional regulator” is broader than the classical definition of a sequence-specific DNA-binding transcription factor and may include chromatin-associated regulatory proteins, transcriptional co-regulators, cofactor-associated regulatory complexes and long non-coding RNA that modulate gene expression without necessarily directly binding DNA motifs [34,35,36,37,38]. In the present study, BTNL9 demonstrated nuclear localization, reproducible chromatin occupancy by ChIP-seq with ChIP-qPCR validation, and coordinated regulation of transcriptional programs identified by RNA-seq, collectively supporting its involvement in transcriptional regulatory processes.

Subcellular fractionation analysis revealed at least three distinct BTNL9 protein bands in NSCLC cell lines, with the highest molecular weight band predominantly localized in the nuclear fraction and the remaining two bands were found mostly in the cytoplasmic fraction. This distribution pattern was accompanied by contextual analysis of predicted NLS motifs across known BTNL9 transcript variants, suggesting that differential nuclear targeting may contribute to the observed subcellular distribution. Despite the presence of predicted NLS motifs within these potential isoforms, the two smaller bands remained predominantly cytoplasmic, and whether these variants may translocate to the nucleus under specific physiological or stress-associated conditions remains to be determined. It is important to note that this subcellular distribution pattern represents a descriptive observation, and the precise identity of the nuclear band and its correspondence to specific BTNL9 transcript variants remains to be directly confirmed through future isoform-specific studies. The transcriptional regulatory function demonstrated in the present study reflects the activity of the overexpressed full-length BTNL9 construct and does not depend on the identity of specific endogenous isoforms. Given the presence of up to 10 reported BTNL9 transcript variants, future studies involving mass spectrometry-based isoform characterization and isoform-specific knockdown approaches will be necessary to determine which transcript variants correspond to the observed protein bands and to clarify their individual functional contributions to transcriptional regulation.

Multiple lines of evidence support a transcriptional regulatory role for BTNL9. Domain analysis revealed a bZIP_GCN4-like region, a structural motif typical of DNA-binding and dimerization domains. Coiled-coil predictions and native PAGE demonstrated that BTNL9 forms homodimers, and co-immunoprecipitation assays confirmed that the bZIP-like region is required for self-association. Immunofluorescence further indicated nuclear localization consistent with chromatin access. ChIP-seq and ChIP-qPCR analyses showed BTNL9 enrichment at promoter regions of genes such as BBC3, CDKN1A, E2F1, and MCM7, all of which contain predicted BTNL9-associated motifs. A DNA-protein pulldown assay using a biotinylated E2F1 promoter fragment demonstrated selective BTNL9 enrichment. Notably, ChIP-derived motif analysis identified a conserved cytosine-rich sequence enriched within BTNL9-associated promoter regions, suggesting a potential sequence preference for genomic association.

Although BTNL9 contains only a single leucine heptad repeat and exhibits a less canonical basic region compared with classical bZIP transcription factors, its genomic distribution supports a broader regulatory role. In this study, 87% of the identified ChIP peaks were located within the intronic and intergenic regions, a pattern commonly observed for transcription factors involved in enhancer activity, long-range chromatin regulation [39,40], or non-coding RNA-associated mechanisms [41]. These possibilities warrant further investigation. Collectively, these findings support a model in which BTNL9 acts as a non-canonical transcriptional regulator with locus-specific genomic engagement and the capacity to repress key cell cycle-related genes. While direct DNA-binding mechanisms remain to be elucidated through EMSA, promoter-reporter assays, and motif-mutation analyses, we acknowledge that the biotinylated promoter pulldown assay was performed using nuclear extract, and therefore indirect association of BTNL9 with the E2F1 promoter fragment via bridging proteins cannot be formally excluded at this stage. Our ChIP-seq and ChIP-qPCR data collectively establish that BTNL9 is functionally associated with transcriptional regulation, though whether this reflects direct sequence-specific DNA contact or indirect chromatin association through protein–protein interactions remains an important open question for future investigation. It is important to note that the chromatin occupancy analyses presented in this study were performed under AM-tagged BTNL9 overexpression conditions, employing a commercially validated ChIP-grade anti-AM-tag antibody, an approach specifically recommended for low-abundance chromatin-associated proteins lacking validated ChIP-grade antibodies. Confirmation of endogenous physiological BTNL9 chromatin occupancy will require future studies employing validated endogenous ChIP-grade antibodies or CRISPR-based endogenous epitope tagging strategies, which will be valuable for defining the full physiological chromatin occupancy landscape of BTNL9.

RNA-seq analysis further suggested that BTNL9 overexpression is associated with altered expression of genes involved in several biological pathways, including cell cycle regulation, a p53-mediated transcriptional regulation program, and DNA replication. These findings identified candidate pathways potentially relevant to the tumor-suppressive functions of BTNL9 and provided a framework for subsequent experimental validation. Specifically, BTNL9 overexpression was associated with coordinated downregulation of multiple MCM family genes, DNA replication licensing factors, and cell-cycle-associated genes, suggesting a potential role in regulating genomic stability and cell-cycle progression. In addition, the RNA-seq dataset identified a subset of p53-associated genes whose expression was altered following BTNL9 overexpression, raising the possibility of functional interplay between BTNL9-mediated transcriptional regulation and p53 signaling pathways. Similar p53-associated regulatory mechanisms have been reported for NF-Y, TFE3, TFEB, FOXD3, and EYA4, which contribute to tumor-suppressive responses through modulation of p53-dependent transcriptional programs [42,43,44,45]. Importantly, many of the key genes selected for validation in this study represent well-established regulators of tumorigenesis. For example, multiple MCM family members have been proposed as sensitive markers of premalignant lung lesions and are closely linked to tumor progression [46,47,48]. Furthermore, several genes downregulated following BTNL9 overexpression, including *CDC25C*, *CDK1*, *E2F1*, *FOXM1*, *MCM2*, and *MCM7*, have previously been identified as therapeutic targets in cancer [49,50,51,52,53,54]. Although the RNA-seq experiment was performed as a discovery-oriented transcriptomic screen, the transcriptional changes observed in key target genes were independently supported by RT-qPCR, Western blot, ChIP-seq, and ChIP-qPCR analyses, providing convergent evidence for the biological relevance of the validated BTNL9-regulated gene network.

Through our xenograft model, we established BTNL9’s role as a tumor suppressor, consistent with its transcriptional and translational repression of genes involved in cell proliferation and DNA replication. Analysis of TCGA datasets further supports this function, showing that BTNL9 downregulation is associated with tumor progression and poorer clinical outcomes. Importantly, survival analyses revealed that patients with higher BTNL9 expression exhibited significantly better overall and disease-free survival in LUAD, and these associations remained robust in multivariate Cox regression models after controlling for age, gender, smoking history, and tumor stage. This indicates that BTNL9 is an independent prognostic factor in LUAD, consistent with our mechanistic findings involving FOXM1 and cell cycle regulation. In contrast, BTNL9 expression did not reach statistical significance in LUSC, highlighting biological differences between adenocarcinoma and squamous cell carcinoma and suggesting that BTNL9’s prognostic utility may be restricted to LUAD. Beyond lung cancer, associations observed in breast, liver, renal, and pancreatic cancers suggest that BTNL9 may have broader relevance as a biomarker, warranting further validation. In addition, BTNL9 expression was associated with complete remission rates after primary treatment, suggesting potential utility as a predictive biomarker for therapeutic response.

Given the molecular heterogeneity of NSCLC, it is important to evaluate the role of BTNL9 across distinct oncogenic subtypes. In the present study, we employed two genetically distinct NSCLC models, A549 (LUAD, harboring KRAS G12S) and NCI-H460 (LCLC, harboring KRAS Q61H), and observed consistent tumor-suppressive effects of BTNL9 in both cell lines, suggesting that its activity is retained in these KRAS-mutant backgrounds. Although TCGA analyses were conducted in both the LUAD and LUSC cohorts, cell-based validation was limited to these two models, as commonly used LUSC cell lines frequently harbor TP53 mutations that are incompatible with the p53-dependent mechanistic framework of this study. Future investigations should extend these findings to other molecularly defined subtypes, including EGFR- and ALK-driven NSCLC, as well as p53 wild-type LUSC models, to determine whether the tumor-suppressive activity and predictive value of BTNL9 are preserved across diverse oncogenic backgrounds and treatment settings. Such subtype-specific validation will be critical for defining the broader clinical relevance of BTNL9 and its potential utility as a biomarker in precision oncology.

The tumor immune microenvironment orchestrates a complex interplay between tumor cells and infiltrating immune populations. This dynamic interaction determines the balance between anti-tumor and pro-tumor immunity, thereby influencing tumor progression and therapeutic efficacy [55,56,57]. Our TCGA analyses of LUAD and LUSC demonstrated a substantial correlation between BTNL9 expression and increased infiltration of immune populations linked to anti-tumor activity, including cytotoxic T cells, NK cells, and B cells. These findings further suggest that BTNL9 may contribute to clinical prognosis and treatment responsiveness through modulation of the immune microenvironment. Further studies are required to elucidate the mechanisms by which BTNL9 may promote immune cell recruitment or retention within tumors. However, such investigations are complicated by structural differences between human and murine BTNL9 proteins, including poor conservation of the putative bZIP domain region in the murine ortholog, which may limit functional comparability across species (Figure S7H). Accordingly, conventional mouse models may not fully recapitulate human BTNL9 biology. Future studies would benefit from patient-derived models or ex vivo human immune co-culture platforms, which provide a genuinely human biological context to more accurately define the immunoregulatory role of BTNL9.

To examine whether the BTNL9 protein level affected treatment efficacy, we selected etoposide and bortezomib as representative agents acting through DNA damage and proteotoxic stress pathways, respectively. Extending these observations to therapeutic response, we found that modulation of BTNL9 expression enhances sensitivity to both etoposide and bortezomib, albeit with distinct response profiles from both NSCLC cell lines. Although bortezomib is not a standard therapeutic agent for NSCLC, its well characterized ability to induce proteotoxic stress and disrupt cell cycle regulation makes it a useful mechanistic tool for interrogating BTNL9-dependent vulnerabilities. We also note that basal cell cycle distributions, particularly the G2/M fraction in asynchronous cultures, showed modest variability between independent experiments, likely reflecting differences in growth conditions and expression levels. Nevertheless, the overall suppressive effect of BTNL9 on cell cycle progression was consistently observed. One of these regulators, FOXM1, is a transcription factor that plays a pivotal role in cell cycle progression, especially during the G1/S and G2/M transition and mitotic progression [58,59,60]. Its activation is also involved in the phosphorylation of CDK1, CDK2, and Cyclin B1 (*CCNB1*) and Aurora kinase B (*AURKB*) [61,62]. A negative correlation between BTNL9 and these key cell cycle regulators in LUAD TCGA data further supports the role of BTNL9 in their suppression. These findings suggest that BTNL9 expression levels in cancer cells play a crucial role in determining treatment efficacy. Based on this novel discovery, we propose that BTNL9 could serve as an important biomarker for guiding treatment selection. Future studies using in vivo drug-response models will be valuable to further assess the translational relevance of BTNL9-mediated chemosensitivity.

In summary, this study provides multiple lines of evidence supporting BTNL9 as a non-canonical transcriptional regulator with tumor-suppressive functions in NSCLC (Figure 7E). Our findings highlight several unique features of BTNL9, including its isoform-specific subcellular localization, homodimerization capacity, and locus-specific genomic association, which together offer new insight into its potential role in cancer biology. While these results suggest that BTNL9 influences key transcriptional regulation in cell cycle progression and stress responses, further mechanistic studies are required to define its gene-specific regulatory roles. It is noteworthy that the majority of BTNL9-associated ChIP-seq peaks reside in intronic and intergenic regions, consistent with the genome-wide distribution patterns commonly observed for chromatin-associated transcriptional regulators. While the present study focused on promoter-proximal BTNL9 occupancy to establish its core transcriptional regulatory function, the potential roles of distal BTNL9-associated chromatin regions including putative enhancer elements and long-range regulatory interactions represent an important avenue for future investigation. Elucidating how BTNL9 expression is controlled in cancer may provide important insights into tumor biology and its potential as a therapeutic target.

## 4. Materials and Methods

### 4.1. Human Lung Tumor and Adjacent Samples

Patients’ clinical unstained slides were obtained from Inje Biobank of Inje University Busan Paik Hospital, a member of Korea Biobank Network with Institutional Review Board regulations and approval (IRB #2023-09-012).

### 4.2. In Vivo Xenograft Mouse Model

The animal study was approved by the Institutional Animal Care and Use Committee of Inje University under the project title” Study of BTNL9 cancer suppression mechanism” (Institutional Review Board# 2022-010), and fully complied with the accepted standards for the care and use of laboratory animals. Four-week old male BALB/C nude mice (Japan SLC) were used for the subcutaneous tumor growth assay. Mice were housed in a 12 h light/12 h dark cycle at 22 °C and 50–60% humidity. Each mouse was subcutaneously injected in the flank with 100 µL of A549 transduced cells (5 × 10^6^) suspended in Matrigel Basement Membrane Matrix (BD Bioscience, San Jose, CA, USA, #356234). Mice were randomly grouped into two (BTNL9 overexpression and empty vector control), each including 6 mice. The experiment was repeated twice (yielding a total of 12 mice per group) to minimize in vivo bias and outliers. Prior to the in vivo study, a plasmid expressing firefly luciferase marker was transfected into A549 cells, which were then selectively cultured with the antibiotic hygromycin B. Once a stable cell line was established, these cells were transduced with either empty vector control or BTNL9-OE lentivirus. At post-transduction 3 days, we implanted these cells subcutaneously into BALB/c nude mice. Tumor volume was measured in every 3 days by an investigator blinded for both experimental groups. Tumor volume was calculated as V = (L × W2)/2, where L is tumor length and W is tumor width in millimeters (mm). The imaging was conducted and captured by In Vivo Imaging Systems (IVIS) at the start (Day 0) and end of the experiment (Day 30) using 30 mg/mL of D-Luciferin (GoldBio, St. Louis, MO, USA, #LUCK-1G). Mice were euthanized using CO_2_ and the tumors were excised for visual and weight-based assessments.

### 4.3. Non-Small Cell Lung Cancer Cell Lines and Cell Culture

Human lung carcinoma cell lines A549 (KCLB) and NCI-H460 (KCLB) were cultured in RPMI-1640 medium (Cytiva, Marlborough, MA, USA, #SH30027), and HEK-293T (ATCC) was cultured in Dulbecco’s Modified Eagle Medium (DMEM) supplemented with 10% fetal bovine serum (FBS) (Cytiva, #SH30919.03) and 1% penicillin/streptomycin (Cytiva, #SV30010). All the cell lines were maintained at 37 °C with 5% CO_2_ and sub-cultured upon reaching approximately 80% confluency.

### 4.4. Plasmid Construction of Human BTNL9

A codon-optimized human *BTNL9* cDNA (RefSeq: NM_152547.5) was synthesized and cloned into the pcDNA3.1(+) vector backbone (Invitrogen, Waltham, MA, USA) and used as a template for subsequent plasmid constructions. For wild-type (WT) BTNL9 overexpression, the cDNA was subcloned into a third-generation lentiviral backbone vector, pLJM1 (Addgene, Watertown, MA, USA, RRID:Addgene_19319), from which the eGFP reporter gene had been removed. The resulting plasmid was designated pBTNL9-OE. The pBTNL9-AM-tag, pBTNL9-FLAG, pBTNL9-ΔbZIP-AM-tag and pBTNL9-MutbZIP-AM-tag expression constructs (Figure S1D), used for ChIP-sequencing, co-immunoprecipitation (co-IP) and native PAGE experiment, were generated with a BTNL9 cDNA template and cloned into the same modified pLJM1 lentiviral backbone. All primers used for amplifying these construct sequences are listed in Table S9. Gibson Assembly was used for all cloning steps.

### 4.5. Lentivirus Production and Infection

Lentiviral particles were produced using a second-generation packaging system according to previous published protocol with minor modification [63]. Three days after transfection, the viral supernatant was harvested, subsequently filtered through 0.45 μm filter to remove cell debris, and then mixed with Lenti-X concentrator at a 3:1 ratio (Takara, Kusatsu, Japan, # 631231). After incubation overnight at 4 °C, the mix was centrifuged at 1500× *g* for 45 min at 4 °C. The concentrated lentivirus was re-suspended in an appropriate volume and kept at −80 °C. A549 and NCI-H460 were subsequently transduced with lentiviruses carrying either an empty vector or overexpression plasmids (pBTNL9-OE and pBTNL9-AM-tag) in the presence of 8 ug/mL hexadimethrine bromide (Sigma-Aldrich, St. Louis, MO, USA, # H9268) overnight. The fresh medium was replaced the next day and cells were harvested as assigned. Due to the potent anti-proliferative effect associated with sustained BTNL9 overexpression, stable puromycin-selected BTNL9-overexpressing NSCLC cell lines could not be maintained during prolonged culture. Preliminary time-course analysis demonstrated that BTNL9 expression peaked at Day 3 following lentiviral transduction and gradually declined thereafter (Figure S3A), likely reflecting selective depletion of highly expressing cells. Functional assays were therefore initiated during the period of peak BTNL9 expression.

### 4.6. Chromatin Immunoprecipitation Sequencing (ChIP-Seq) and ChIP-qPCR

ChIP DNA was prepared using Tag-ChIP-IT (Active Motif, Carlsbad, CA, USA, #53022) according to the manufacturer’s protocol. A549 and NCI-H460 cells were infected with lentivirus overexpressing pBTNL9-AM-tag and cross-linked with 1% formaldehyde fixative solution on Day 3 before being quenched after 15 min. Following 2 rounds of washing, cell pellets were incubated in Chromatin Prep Buffer for 10 min, followed by 20 strokes of processing with a chilled Dounce homogenizer. After centrifugation, cells were resuspended in ChIP buffer before sonication to yield fragments between 200 and 500 bp in length. Four ug of Antibodies for anti-AM-tag (Active Motif, #91111, RRID:AB_2793779), anti-mouse H3K27ac (Thermo Fisher Scientific, Waltham, MA, USA, #MA5-23516, RRID:AB_2608307) or mouse IgG2a isotype control (Thermo Fisher Scientific, #02-6200, AB_2532943) were added to 30 µg sheared chromatin and incubated at 4 °C overnight. ChIP DNA and input DNA were extracted using the column provided by this kit. Both ChIP DNA and input DNA samples were either analyzed via ChIP-qPCR or submitted to BGI Genomics (Hong Kong, China) for ChIP-seq library construction and sequencing. ChIP DNA was prepared using the DNBSEQ™ MGISEQ-2000RS platform (MGI Tech, Shenzhen, China). Briefly, DNA ends were repaired, 3′-dA overhangs were added, and sequencing bubble adaptors were ligated. The DNA fragments were amplified, heat-denatured, and circularized to form single-strand circular libraries prior to sequencing. Raw sequencing data were filtered to remove adapter sequences, contaminants, and low-quality reads using SOAPnuke (CleanParameter: filter -l 5 -q 0.5 -n 0.1 -Q 2). The resulting clean reads were aligned to the human reference genome (GRCh37/hg19) using BWA (version 0.7.10; parameters: bwa aln -t 4 -o 1 -e 49 -m 100,000 -i 15 -q 10 -I) and SOAPaligner/SOAP2 (version 2.21t, RRID:SCR_005503). For group-level peak calling, sequencing reads from both biological replicates (A549-BT-rep1 and A549-BT-rep2) were pooled and analyzed jointly against pooled input controls (A549-Input-rep1 and A549-Input-rep2) using MACS2 (RRID:SCR_013291; parameters: -g hs -s 50 -p 1.0 × 10^−5^ -m 10 30 -B --trackline). Peaks were identified using a threshold of *p* < 1.0 × 10^−5^ and a Benjamini–Hochberg false discovery rate (FDR) < 0.005, yielding 26,610 high-confidence BTNL9 associated genomic regions. Pooling of biological replicates prior to peak calling is an established strategy to increase the sequencing depth and sensitivity for detecting chromatin-associated regions, particularly for proteins with low endogenous expression. To associate peaks with nearby genes, genomic coordinates of the significant peaks were uploaded to the Genomic Regions Enrichment of Annotations Tool (GREAT, version 4.0.4, RRID:SCR_005807), using a window of ±1 kb around the transcription start site (TSS), identifying 9709 BTNL9-associated genomic regions proximal to annotated genes. To validate ChIP-seq data, enrichment at a selected gene locus was quantified using the percent of input method and confirmed specific BTNL9 binding in both cell lines. In addition, 10% of sheared chromatin as input was purified together with final ChIP-DNA. Equal amounts of ChIP-DNA from IgG2a isotype control and BTNL9-AM-tag pulled down groups were used as templates in ChIP-qPCR. Primers are listed in Table S9.

### 4.7. Co-Immunoprecipitation (Co-IP) Protein Pull-Down Assay

Both A549 and NCI-H460 cells were co-infected with lentiviral generated from pCMV-BTNL9-AM-tag and pCMV-BTNL9-FLAG-tag. Two million cells were seeded in a 2 × 10 cm culture dish (Corning, Corning, NY, USA, #430167) and cells were harvested at Day 3. Co-IP was performed using Pierce crosslink immunoprecipitation kit (Thermo Fisher Scientific, #26147) according to the manufacturer’s instructions. In addition, 10 µg of anti-FLAG antibody (Biolegend, San Diego, CA, USA, #637304) was crosslinked to Protein A/G Plus Agarose and used as bait in the Co-IP process, which was followed by overnight incubation with 500 µg of pre-clear lysate at 4 °C overnight. The eluted BTNL9-AM-tag immunoprecipitated protein was analyzed by Western blot.

### 4.8. Native Polyacrylamide Gel Electrophoresis (PAGE)

Briefly, samples were harvested in RIPA Lysis and Extraction Buffer (Thermo Fisher Scientific, #89900) without any agitation on ice for 30 min. Following centrifugation, protein concentration was quantified using Pierce BCA kit (Thermo Fisher Scientific, # 23225). For native PAGE, samples were prepared in Pierce™ LDS Sample Buffer, Non-Reducing (Thermo Fisher Scientific, #84788), while denatured samples were boiled before being loaded into the NativePAGE™ Bis-Tris Mini Protein Gels, 3–12% (Thermo Fisher Scientific, #BN1001BOX). The gel was run for 1.5 h using a cathode (Thermo Fisher Scientific, #BN2002) and NativePAGE™ Running Buffer (Thermo Fisher Scientific, #BN2001). Proteins were transferred onto a PVDF membrane for Western blot analysis.

### 4.9. MEME-ChIP DNA Motif Analysis

Both BTNL9 ChIP peak replicate samples were further processed to meet the criteria before being submitted for motif analysis. Peaks with a Benjamini–Hochberg FDR < 0.005 were selected to extract 500 bp from the central region of each peak and generated the final BED format. In the CentriMo option within the MEME (version 5.5.9, RRID:SCR_001783) [64], a maximum region width of 200 bp was set for motif analysis. The only motif sequence that appeared from both replicate samples analyses was selected as the conserved motif in this study.

### 4.10. Generation of Sequence Logos Using WebLogo 3

Conserved BTNL9 motif sequence logos were generated using the WebLogo 3 (https://weblogo.berkeley.edu). Briefly, 19 bp of adjacent BTNL9 putative binding sites aligned nucleotide sequences in FASTA format were uploaded to the WebLogo interface. The output logo depicted the relative frequency and information content of residues at each position, with symbol height being proportional to conservation. Logos were exported in a high resolution image format (PDF) for inclusion in figure.

### 4.11. DNA-Protein Pulldown (Streptavidin-Biotin Assay)

A 237 bp biotinylated DNA fragment corresponding to the E2F1 promoter region containing the multiple predicted BTNL9 binding motif was synthesized and purified (Table S9). Nuclear extracts were prepared from A549 cells transiently overexpressing FLAG-tagged BTNL9 using a standard nuclear extraction protocol. The biotinylated DNA probe was incubated with nuclear extracts at 4 °C to allow DNA-protein complex formation. Complexes were captured using Dynabeads MyOne Streptavidin T1 magnetic beads (Thermo Fisher Scientific, #65602) according to the manufacturer’s instructions. After sequential washes [3 rounds of 1× EMSA buffer (20 mM HEPES (pH 7.2), 32 mM KCl, 0.1 mM EDTA (pH 8), 10% glycerol, 70 ng/μL BSA, 8 ng/μL poly dI-dC and 2.5 mM DDT)], including stringent conditions with additional 0.2% Tween-20, bound proteins were eluted and analyzed by SDS-PAGE followed by immunoblotting with anti-FLAG antibody (1:1000 dilution).

### 4.12. RNA-Sequencing (RNA-Seq)

The total RNA was isolated using Trizol reagent (Thermo Fisher Scientific, # 15596026) and sent to RNA-sequencing service provider (Ebiogen, Seoul, Republic of Korea). RNA quality was assessed by Agilent TapeStation 4000 system (Agilent Technologies, Santa Clara, CA, USA), and RNA quantification was performed using ND-2000 Spectrophotometer (Thermo Fisher Scientific). Library construction was performed using QuantSeq 3′ mRNA-Seq Library Prep Kit (Lexogen, Vienna, Austria) according to the manufacturer’s instructions. This 3′ end sequencing approach enables accurate gene-level expression quantification but precludes detection of alternative splicing events or isoform-level transcript changes, which were not objectives of the present study. High-throughput sequencing was performed as single-end 75 sequencing using NextSeq 550 (Illumina, San Diego, CA, USA). The sequencing raw data was analyzed by ROSALIND^®^ (https://rosalind.bio/, RRID:SCR_006233), with a HyperScale architecture developed by ROSALIND, Inc. (San Diego, CA, USA). Reads were trimmed using cutadapt1. Quality scores were assessed using FastQC2. Reads were aligned to the Homo sapiens genome build hg19 using STAR3. Individual sample reads were quantified using HTseq4 and normalized via Relative Log Expression (RLE) using DESeq2 R library (version 1.48.0). Read Distribution percentages, violin plots, identity heatmaps, and sample multidimensional scaling (MDS) plots were generated as part of the QC step using RSeQC6. DEseq2 [65] was also used to calculate fold changes and *p*-values and performed optional covariate correction. Clustering of genes for the final heatmap of differentially expressed genes (DEGs) was done using the PAM (Partitioning Around Medoids) method using the fpc R library (version 2.2-13). Hypergeometric distribution was used to analyze the enrichment of pathways, gene ontology, domain structure, and other ontologies. The topGO R library (version 2.62.0) was used to determine local similarities and dependencies between GO terms in order to perform Elim pruning correction. Several database sources were referenced for enrichment analysis, including Interpro9, NCBI10, MSigDB11,12, REACTOME13, and WikiPathways. Enrichment was calculated relative to a set of background genes pertinent to the study. RNA-seq was performed with two biological replicates per group, with a discovery-oriented FDR threshold of *q* < 0.1 applied to maximize detection sensitivity. RNA-seq transcriptomic profiling was performed exclusively in A549 cells. Both A549 and NCI-H460 cells were used for targeted RT-qPCR validation of 12 selected DEGs to assess cross-cell-line consistency of key transcriptional changes, and do not constitute independent transcriptome-level replication.

### 4.13. RNA Extraction and cDNA Synthesis

Total RNA for qPCR was extracted using a TaKaRa MiniBEST Universal RNA Extraction Kit (Takara Bio, Kusatsu, Japan, #9767A), according to the manufacturer’s protocol. RNA concentration and quality were measured with Nanodrop 2000 (Thermo Fisher Scientific). cDNA was synthesized from 2 μg of total RNA in 20 μL reactions using High-Capacity cDNA Reverse Transcription Kit (Thermo Fisher Scientific, #4368814). After synthesis, the cDNA was diluted 4 times with double distilled water and stored at −20 °C.

### 4.14. Quantitative Real-Time PCR (qPCR)

Quantitative real-time PCR following reverse transcription (RT-qPCR) was performed in a CFX Opus 96 Real-Time PCR System (Bio-Rad, Hercules, CA, USA) using KAPA SYBR FAST Universal Kit (Sigma-Aldrich, #KK4601). For quantification using the comparative CT method of gene expression, corresponding primers used in this study are listed in Table S9. All reactions were performed in triplicate and normalized to GAPDH, which served as a control housekeeping gene.

### 4.15. Immunofluorescence Staining and Confocal Microscopy Analysis

Both A549 and NCI-H460 cells were seeded on poly-l-lysine (Sigma-Aldrich, #P8920) coated slide overnight prior to the process for immunofluorescence staining. Cells were fixed with 4% Paraformaldehyde at room temperature for 15 min and permeabilized with 0.3% Triton-X-100 in 5% BSA for 15 min, followed by 5% goat serum for 1 h. Either 1:250 dilution of anti-rabbit BTNL9 antibody (LS-Bio, Seattle, WA, USA, #LS-C399149) or rabbit IgG control was added for overnight incubation. Slides were washed and incubated at room temperature with 1:1000 diluted anti-rabbit IgG Alexa Fluor^®^ 594-conjugated (Thermo-Fisher Scientific, #A32740, RRID:AB_2762824) for 1 h, followed by DAPI to counterstain nuclei. Slides were mounted with VECTASHIELD^®^ Antifade Mounting Medium (Vector Laboratories, Newark, CA, USA, #H-1000-10). All confocal images were captured with Nikon Confocal laser microscope A1+ at 40× lens. Confocal images were presented without cropping or digital manipulation.

### 4.16. Western Blot

Cells were lysed in RIPA Lysis and Extraction Buffer (Thermo Fisher Scientific, #89900) with Halt™ Protease and Phosphatase Inhibitor Cocktail (Thermo Fisher Scientific, #78440). The concentration of protein lysate was measured using Pierce™ BCA Protein Assay Kits (Thermo Fisher Scientific, #23225). Then, 30 µg of protein lysate was resolved by 10% SDS-PAGE and transferred to Immobilon-PSQ PVDF Membrane (Sigma-Aldrich, #ISEQ00010). Transferred membranes were incubated with a 5% Blotting-Grade Blocker (Bio-Rad, #1706404), followed by 1:1000 dilution of primary antibodies for overnight incubation. All primary antibodies and secondary antibodies used in this study are tabulated in Table S10 with an RRID number. The HRP-conjugated secondary antibody was incubated at room temperature for 1 h prior to signal development by Immobilon Forte Western HRP substrate (Sigma-Aldrich, #WBLUF0500). All images were captured with iBright CL1500 (Thermo Fisher Scientific, #A43678) and the signal intensity was quantified with iBright Analysis software version 5.2.2.

### 4.17. Fractionation Assay

To determine the subcellular localization of BTNL9, we performed the fractionation assay as described in a reference paper with minor modifications [66]. Briefly, 5 million cells were used for whole cell lysate (WCL), and isolation of cytoplasm fraction (Cyt) and nucleus fraction (Nuc). Additionally, 15 µL of each fraction were loaded for Western blot.

### 4.18. Proliferation Assay

Cell proliferation was determined by seeding 500 cells/well for A549 cell line and 150 cells/well for NCI-H460 with triplicate wells in a 96-well microplate (Corning, #3596). One tenth of the growth medium’s volume of Resazurin (R&D Systems, Minneapolis, MN, USA, #AR002) was added from Day 0 onward and incubated for 4 h at 37 °C in the presence of 5% CO_2_. Fluorescence was measured using the microplate reader Viroskan Lux (Thermo Fisher Scientific, #VLBL00D0) by setting the excitation at 544 nm and emission at 590 nm in Skanlt software 6.0.1 (Thermo Fisher Scientific). Multiple points per well were selected to eliminate cross-emission between wells. Growth curves were then generated by subtracting blank readings and normalizing to the initial reading on Day 0.

### 4.19. Cytotoxicity Assay

To assess the effect of BTNL9 overexpression on drug sensitivity, A549 and NCI-H460 cells were infected with lentivirus carrying either a vector control or BTNL9 overexpression construct. The following day, cells were counted and seeded at a density of 5000 cells per well in 96-well plates in triplicate for each condition. On Day 2, either etoposide (Sigma-Aldrich, #E1383-25mg) or bortezomib (Sigma-Aldrich, #5043140001) were added to the wells at a range of concentrations to generate dose–response curves, and control wells were treated with the vehicle only. Cells were incubated with the drugs for 48 h at 37 °C in a humidified incubator with 5% CO_2_. After the incubation period, resazurin solution was added and proceeded following the same method as the proliferation assay. The fluorescent signal was normalized to the vehicle control, and dose–response curves were plotted using PRISM software (version 8.3.1). The IC50 values were calculated for both vector control and BTNL9-overexpressing cells to evaluate the impact of BTNL9 on drug sensitivity. Drug concentrations were selected to produce a robust mechanistic signal in vitro and are not intended to reflect clinical pharmacokinetics.

### 4.20. Cell Cycle Assay

To assess cell proliferation in a cell-cycle dependent manner, transfected cells were seeded in a 6-well microplate (NEST, Wuxi, China, #703003). After 24 h, cells were pulsed with 10 µM of BrdU (Sigma-Aldrich, #B5002) for 1 h, harvested, and then stained using BrdU cell proliferation assay kit (BD Bioscience, San Jose, CA, USA, #556028). Flow cytometry was performed using CytoFlex (Beckman Coulter, Brea, CA, USA, #A001-1-1102). The Flow cytometry data was analyzed by FlowJo software (version 10.2, RRID:SCR_008520). These experiments were repeated twice.

### 4.21. Clonogenic Assay

Initially, we determined the plating efficiency by seeding varying numbers of cells. For A549, 500 cells were seeded and for NCI-H460, 250 cells were seeded per 6 cm plate, with the experiments done in biological replicates and repeated twice (SPL Life Sciences, Pocheon, Republic of Korea, #20060). Plates were incubated at 37 °C in the presence of 5% CO_2_ for 10 days until colonies became visually apparent. The plates were stained with 0.1% crystal violet solution containing 20% of methanol for 20 min and gently rinsed with running water. Plate images were captured and colonies were counted with ImageJ (version 2.14.0/1.54f, National Institutes of Health, RRID:SCR_003070) [67].

### 4.22. Soft Agar Assay

To determine anchorage-independent growth, soft agar assay was performed twice with biological triplicates. Low-gelling temperature agarose (Sigma-Aldrich, #A9414-25G) was dissolved in ultra-sterile water to make 2% agarose stock gel. The gel was liquefied by heating and kept in a water bath set at 45 °C. The plate was prewarmed inside incubator at 37 °C and coated with a 0.6% diluted gel to create a bottom layer, which was then solidified at 4 °C for 30 min. The base-coated plate was prewarmed again at 37 °C and an adequate cell number (10,000 cells for A549, and 600 cells for NCI-H460 per well) was added into the 0.3% agarose containing growth media. The suspension was mixed gently avoiding bubble formation and dispensed evenly on top of the base layer. The plate was then incubated at 4 °C for an additional 30 min, after which 1.5 mL of media was added to prevent the agar from disintegrating. After 2–3 weeks, colonies were stained by 1 mg/mL of Nitro blue Tetrazolium Chloride (NBT) (Thermo Fisher Scientific, #N6495) at 37 °C overnight. Captured images were analyzed by OpenCFU (version 3.9.0) [68].

### 4.23. Immunohistochemistry Staining

IHC was conducted on paraffin-embedded tissue of normal lung tissue and lung tumor obtained from 98 LUAD patients (45 male, 53 female; mean diagnosis age 59.9 years; tumor stage I–IV; histological grade 2–3) under IRB approval (#2023-09-012) as described in Section 4.1. The intensity of cytoplasmic staining was evaluated semi-quantitatively by a pathologist as low, intermediate, or high grade. For comparative analysis, cases were grouped into Strong (high staining intensity; n = 25) and Weak (low-to-intermediate staining intensity; n = 73) expression categories. Baseline clinical characteristics including sex, diagnosis age, tumor stage, histological grade, tumor size, pleural invasion, lymphovascular invasion, and EGFR mutation status were comparable between groups (all *p* > 0.05). Slides were heated for 1 h at 60 °C, deparaffinized in xylene, rehydrated in graded alcohol and rinsed in distilled water. Antigen retrieval and immunohistochemistry were performed using either a Benchmark XT or Discovery XT automated immunohistochemistry system (Ventana Medical Systems, Inc., Tucson, AZ, USA) with an OptiView DAB IHC Detection kit. Slides were incubated for 34 min at room temperature with diluted BTNL9 antibody to 1:100 (LS-Bio; #LS-C399149). After antibody staining, sections were washed in distilled water, lightly counterstained with hematoxylin, rehydrated and mounted with coverslips. IHC images were obtained on a NanoZoomer RS Digital Pathology System scanner. The intensity of cytoplasmic staining was evaluated as low, intermediate and high grade.

### 4.24. Generation of p53 Knockout (KO) Cell Lines Using CRISPR-Cas9

CRISPR-Cas9-mediated knockout of *TP53* was performed in A549 and NCI-H460 cells. Single-guide RNAs (sgRNAs) targeting exon 6 of the *TP53* gene were adopted from a previously published study [69] and cloned into a modified lentiviral HypaCas9 vector (Addgene, RRID:Addgene_138557), which had been engineered to include a U6 promoter and sgRNA scaffold for sgRNA expression. Lentiviral particles were produced in HEK-293T cells using standard packaging plasmids (psPAX2 and pMD2.G) and used to transduce the target cells in the presence of polybrene (8 μg/mL). Transduced cells were selected with blasticidin (10 μg/mL) for 10 days. The knockout efficiency was confirmed by immunoblotting for p53 protein.

### 4.25. Cox Regression and Forest Plot Analysis

Survival analysis was performed using the Posit R platform (formerly RStudio, version 2025.05.1). Clinical and molecular data (extracted from cBioportal for LUAD and LUSC) were imported into R (version 4.5.3), and survival time and event status were defined as outcome variables. A Cox proportional hazards regression was conducted using the survival package (version 3.8.3) to estimate hazard ratios (HRs) and 95% confidence intervals (CIs) for BTNL9 expression and relevant covariates. Model assumptions were verified by testing proportional hazards with Schoenfeld residuals. Forest plots were generated using the forestplot (version 3.1.6) and ggplot2 packages (version 4.0.3) to visualize HRs across subgroups. Each plot displayed point estimates (HRs) as squares and 95% CIs as horizontal lines, with the line of unity (HR = 1) indicated for reference. 

### 4.26. Quantification and Statistical Analysis

All experiments were carried out independently three times. Data were expressed as mean ± standard deviation (SD) and compared using Student’s *t*-test and two-way ANOVA. Differences with *p* < 0.05 were considered statistically significant (* *p* < 0.05, ** *p* < 0.01, and *** *p* < 0.001). Data were processed with Graph PadPrism for Windows, version 8 (Graph Pad Software Inc., San Diego, CA, USA).

### 4.27. Use of Artificial Intelligence Tools

The graphical abstract was generated using ScholarViz (Nano Banana Pro; https://scholarviz.com). AI-assisted tools were used for language refinement during manuscript preparation. All scientific content, data interpretation, and conclusions remain solely the responsibility of the authors.

## Data Availability

All data associated with this study are available in the main text or the Supplementary Materials. ChIP-seq and RNA-seq data have been deposited into the NCBI’s Short Read Archive (RRID:SCR_004801; BioProject ID PRJNA1011818) with Series GSE242317 (ChIP-seq) and Series GSE242319 (RNA-seq). This study did not generate any original code. Any additional information required to reanalyze the data reported in this paper and requests for materials generated in this study should be directed to the lead contact, Inhak Choi (miccih@inje.ac.kr), and are available with a completed materials transfer agreement.

## Supplementary Materials

The following supporting information can be downloaded at: https://www.mdpi.com/article/10.3390/ijms27156598/s1.
