# Supplementary material for: Immunoglobulin Superfamily Protein BTNL9 Functions as a Non-Canonical Transcriptional Regulator to Suppress NSCLC Through Cell Cycle and p53 Pathways
Source: Int J Mol Sci. 2026 Jul 24;27(15):6598. doi: 10.3390/ijms27156598 (PMC13466322; doi:10.3390/ijms27156598)
Supplement: Supplementary file 1 [file ijms-27-06598-s001.zip › Suppl figures & tables.pdf]

International Journal of *Molecular Sciences*

MDPI

Supplementary Information

Immunoglobulin Superfamily Protein BTNL9 Functions as a Non-Canonical Transcriptional Regulator to Suppress NSCLC Through Cell Cycle and p53 Pathways

Wooi Loon Ng [1,2,*,†], Pedram Yadollahi [1,2,†,‡], Hwa Jin Cho [3,§], Mi Seon Kang [3] and Inhak Choi [1,2,*]

[1] Innovative Therapeutics Research Institute, Inje University, Busan 47397, Republic of Korea;

[2] Department of Microbiology and Immunology, Inje University College of Medicine, Busan 47397, Republic of Korea;

[3] Department of Pathology, Busan Paik Hospital, Inje University, Busan 47397, Republic of Korea; eunhasu101@hanmail.net (H.J.C.); pathmsk@hanmail.net (M.S.K.)

[*] Correspondence: miccih@inje.ac.kr (I.C.); wooiloon@yahoo.com (W.L.N.)

[†] These authors contributed equally to this work.

[‡] Current address: Bobby R. Alford Department of Otolaryngology Head and Neck Surgery, Baylor College of Medicine, Houston, TX 77030, USA.

[§] Current address: Department of Pathology, Busan & Gyeongnam Reference Lab, Seegene Medical Foundation, Busan 48792, Republic of Korea.

Figures S1, S2, S3, S4, S5, S6 & S7
Tables S3, S7, S9 & S10

Supplementary Figures

Figure S1

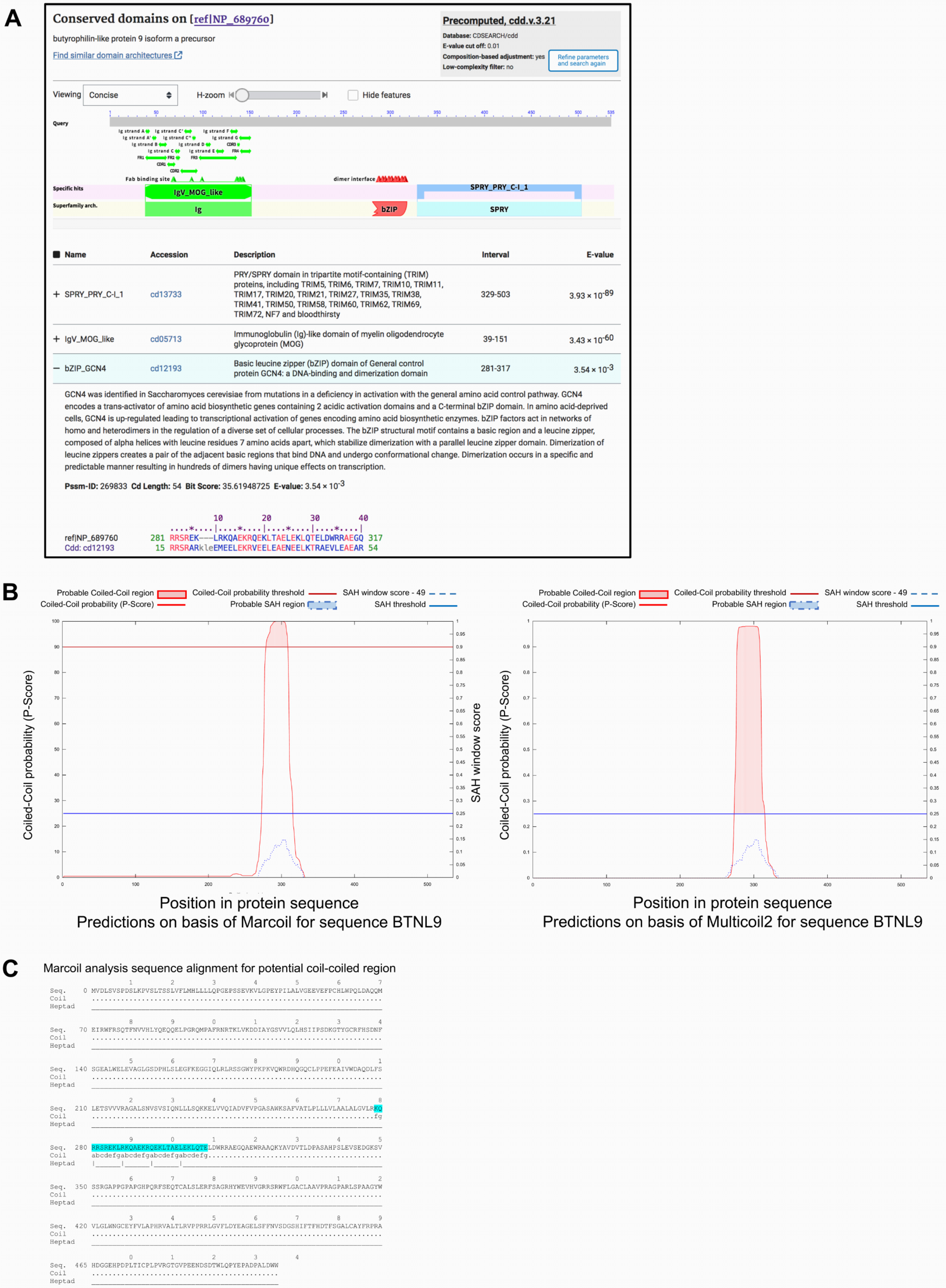

Figure S1

D

BTNL9 ΔbZIP

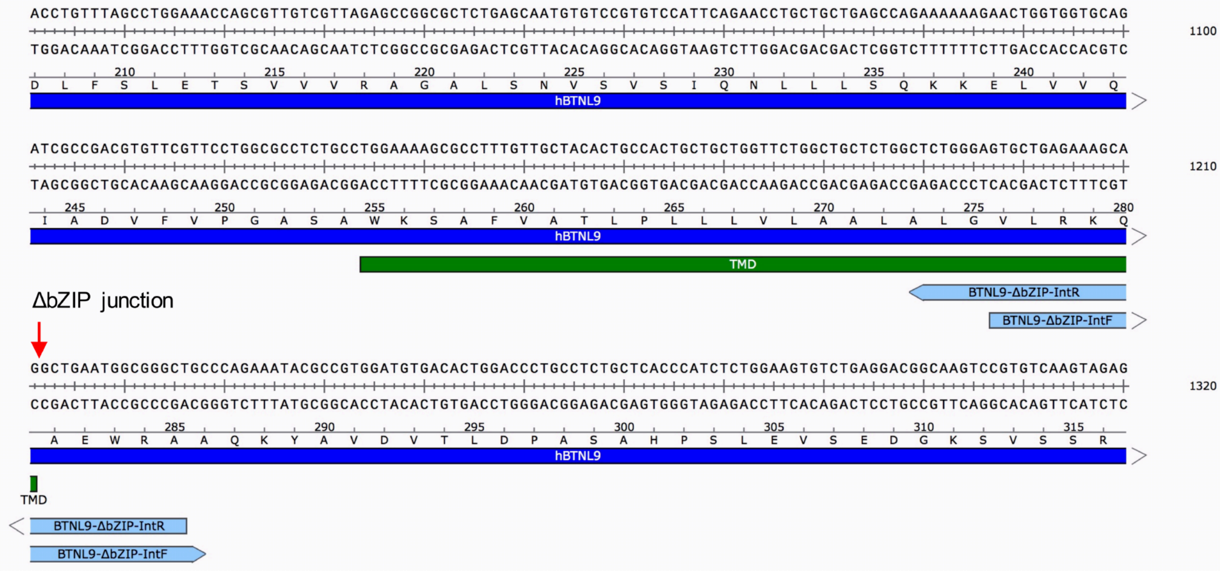

BTNL9 bZIP-mutant

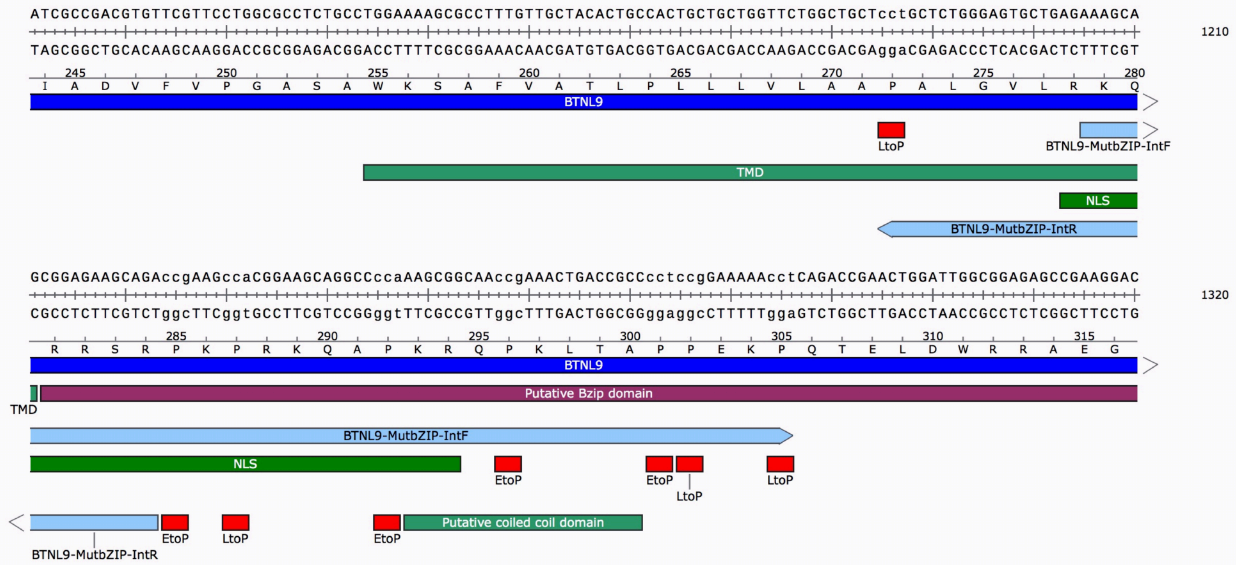

Figure S1

E

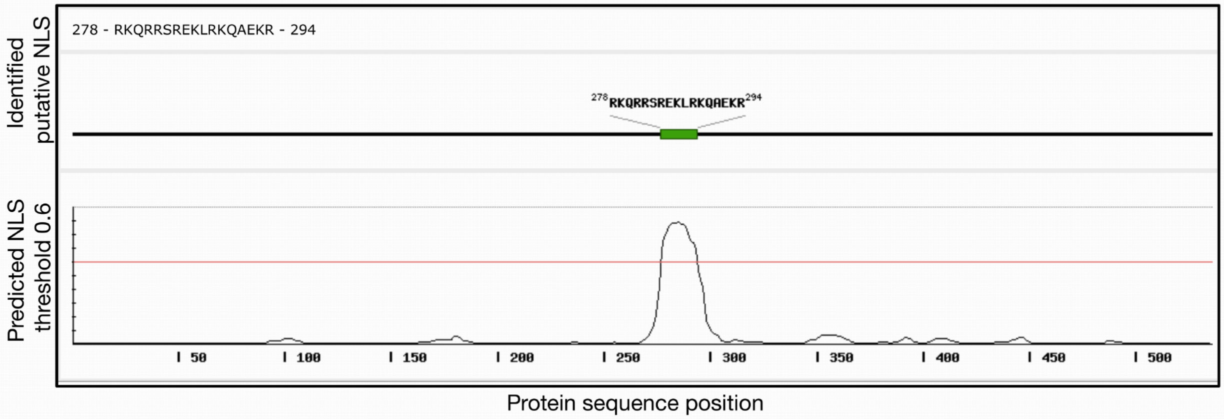

F

```
CLUSTAL O(1.2.4) multiple sequence alignment

V1      mvdlsvspdslkpvsltsslvflmhllllqpgepssevkvlgpeypilalvgeevefpch    60
V2      mvdlsvspdslkpvsltsslvflmhllllqpgepssevkvlgpeypilalvgeevefpch    60
X1      mvdlsvspdslkpvsltsslvflmhllllqpgepssevkvlgpeypilalvgeevefpch    60
X2      mvdlsvspdslkpvsltsslvflmhllllqpgepssevkvlgpeypilalvgeevefpch    60
X3      mvdlsvspdslkpvsltsslvflmhllllqpgepssevkvlgpeypilalvgeevefpch    60
X4      mvdlsvspdslkpvsltsslvflmhllllqpgepssevkvlgpeypilalvgeevefpch    60
X5      ------------------------------------------------------------    0
X6      mvdlsvspdslkpvsltsslvflmhllllqpgepssevkvlgpeypilalvgeevefpch    60
X7      mvdlsvspdslkpvsltsslvflmhllllqpgepssevkvlgpeypilalvgeevefpch    60
X8      ------------------------------------------------------------    0
X9      ------------------------------------------------------------    0

V1      lwpqldaqqmeirwfrsqtfnvvhlyqeqqelpgrqmpafrnrtklvkddiaygsvvlql    120
V2      lwpqldaqqmeirwfrsqtfnvvhlyqeqqelpgrqmpafrnrtklvkddiaygsvvlql    120
X1      lwpqldaqqmeirwfrsqtfnvvhlyqeqqelpgrqmpafrnrtklvkddiaygsvvlql    120
X2      lwpqldaqqmeirwfrsqtfnvvhlyqeqqelpgrqmpafrnrtklvkddiaygsvvlql    120
X3      lwpqldaqqmeirwfrsqtfnvvhlyqeqqelpgrqmpafrnrtklvkddiaygsvvlql    120
X4      lwpqldaqqmeirwfrsqtfnvvhlyqeqqelpgrqmpafrnrtklvkddiaygsvvlql    120
X5      ---------meirwfrsqtfnvvhlyqeqqelpgrqmpafrnrtklvkddiaygsvvlql    51
X6      lwpqldaqqmeirwfrsqtfnvvhlyqeqqelpgrqmpafrnrtklvkddiaygsvvlql    120
X7      lwpqldaqqmeirwfrsqtfnvvhlyqeqqelpgrqmpafrnrtklvkddiaygsvvlql    120
X8      ------------------------------------------------------------    0
X9      ------------------------------------------------------------    0

V1      hsiipsdkgtygcrfhsdnfsgealwelevaglgsdphlslegfkeggiqlrlrssgwyp    180
V2      hsiipsdkgtygcrfhsdnfsgealwelevaglgsdphlslegfkeggiqlrlrssgwyp    180
X1      hsiipsdkgtygcrfhsdnfsgealwelevaglgsdphlslegfkeggiqlrlrssgwyp    180
X2      hsiipsdkgtygcrfhsdnfsgealwelevaglgsdphlslegfkeggiqlrlrssgwyp    180
X3      hsiipsdkgtygcrfhsdnfsgealwelevaglgsdphlslegfkeggiqlrlrssgwyp    180
X4      hsiipsdkgtygcrfhsdnfsgealwelevaglgsdphlslegfkeggiqlrlrssgwyp    180
X5      hsiipsdkgtygcrfhsdnfsgealwelevaglgsdphlslegfkeggiqlrlrssgwyp    111
X6      hsiipsdkgtygcrfhsdnfsgealwelevaglgsdphlslegfkeggiqlrlrssgwyp    180
X7      hsiipsdkgtygcrfhsdnfsgealwelevaglgsdphlslegfkeggiqlrlrssgwyp    180
X8      ------------------------------------------------------------    0
X9      ------------------------------------------------------------    0

V1      kpkvqwrdhqgqclppefeaivwdaqdlfsletsvvvragalsnvsvsiqnlllsqkkel    240
V2      kpkvqwrdhqgqclppefeaivwdaqdlfsletsvvvragalsnvsvsiqnlllsqkkel    240
X1      kpkvqwrdhqgqclppefeaivwdaqdlfsletsvvvragalsnvsvsiqnlllsqkkel    240
X2      kpkvqwrdhqgqclppefeaivwdaqdlfsletsvvvragalsnvsvsiqnlllsqkkel    240
X3      kpkvqwrdhqgqclppefeaivwdaqdlfsletsvvvragalsnvsvsiqnlllsqkkel    240
X4      kpkvqwrdhqgqclppefeaivwdaqdlfsletsvvvragalsnvsvsiqnlllsqkkel    240
X5      kpkvqwrdhqgqclppefeaivwdaqdlfsletsvvvragalsnvsvsiqnlllsqkkel    171
X6      kpkvqwrdhqgqclppefeaivwdaqdlfsletsvvvragalsnvsvsiqnlllsqkkel    240
X7      kpkvqwrdhqgqclppefeaivwdaqdlfsletsvvvragalsnvsvsiqnlllsqkkel    240
X8      ------------------------------------------------------------    0
X9      ------------------------------------------------------------    0

V1      vvqiadvfvpgasawksafvatlplllvlaalalgvlrkqrrsreklrkqaekrqeklta    300
V2      vvqiadvfvpgasawksafvatlplllvlaalalgvlrkqrrsreklrkqaekrqeklta    300
X1      vvqiadvfvpgasawksafvatlplllvlaalalgvlrkqrrsreklrkqaekrqeklta    300
X2      vvqiadvfvpgasawksafvatlplllvlaalalgvlrkqrrsreklrkqaekrqek---    297
X3      vvqiadvfvpgasawksafvatlplllvlaalalgvlrkqrrsreklrkqaekrqek---    297
X4      vvqiadvfvpgasawksafvatlplllvlaalalgvlrkqrrsreklrkqaekrq-----    295
X5      vvqiadvfvpgasawksafvatlplllvlaalalgvlrkqrrsreklrkqaekrqeklta    231
X6      vvqiadvfvpgasawksafvatlplllvlaalalgvlrkqrrsreklrkqaekrqeklta    300
X7      vvqiadvfvpgasawksafvatlplllvlaalalgvlrkqrrsreklrkqaekrq-----    295
X8      -------mqkg------sqgcalphplpgasl------paqpgpadhrgwecrigeklta    41
X9      -------mqkg------sqgcalphplpgasl------paqpgpadhrgwecrigeklta    41
               :  *     :  .:**  *  *:*      : .  . *    :

V1      elekLqteldwrraegqaewraaqkya-----------------------------vdvt    331
V2      eleklqteldwrraegqaecfvlashppg-----------egiqaasnstt--------t    341
X1      eleklqteldwrraegqaewraaqkyaekrevcppwgrhgrgpdrpgraltlwlcavdvt    360
X2      ----lqteldwrraegqaewraaqkya-----------------------------vdvt    324
X3      ----ltaeldwrraegqaewraaqkya-----------------------------vdvt    324
X4      ---------dwrraegqaewraaqkya-----------------------------vdvt    317
X5      eleklqteldwrraegqaewraaqkya-----------------------------vdvt    262
X6      eleklqteldwrraegqaecfvlashppg-----------egiqaasnstt--------t    341
X7      ---------dwrraegqaecfvlashppg-----------egiqaasnstt--------t    327
X8      eleklqteldwrraegqaewraaqkya-----------------------------vdvt    72
X9      -------eldwrraegqaewraaqkya-----------------------------vdvt    65
                 **********  .  .:                              *

V1      ldpasahpslevsedgksvssrgappgpapghpqrfseqtcalslerfsagrhywevhvg    391
V2      lna---------------------------------------------------------    344
X1      ldpasahpslevsedgksvssrgappgpapghpqrfseqtcalslerfsagrhywevhvg    420
X2      ldpasahpslevsedgksvssrgappgpapghpqrfseqtcalslerfsagrhywevhvg    384
X3      ldpasahpslevsedgksvssrgappgpapghpqrfseqtcalslerfsagrhywevhvg    384
X4      ldpasahpslevsedgksvssrgappgpapghpqrfseqtcalslerfsagrhywevhvg    377
X5      ldpasahpslevsedgksvssrgappgpapghpqrfseqtcalslerfsagrhywevhvg    322
X6      lna---------------------------------------------------------    344
X7      lna---------------------------------------------------------    330
X8      ldpasahpslevsedgksvssrgappgpapghpqrfseqtcalslerfsagrhywevhvg    132
X9      ldpasahpslevsedgksvssrgappgpapghpqrfseqtcalslerfsagrhywevhvg    125
        *:

V1      rrsrwflgaclaavpragparlspaagywvlglwngceyfvlaphrvaltlrvpprrlgv    451
V2      ------------------------------------------------------------    344
X1      rrsrwflgaclaavpragparlspaagywvlglwngceyfvlaphrvaltlrvpprrlgv    480
X2      rrsrwflgaclaavpragparlspaagywvlglwngceyfvlaphrvaltlrvpprrlgv    444
X3      rrsrwflgaclaavpragparlspaagywvlglwngceyfvlaphrvaltlrvpprrlgv    444
X4      rrsrwflgaclaavpragparlspaagywvlglwngceyfvlaphrvaltlrvpprrlgv    437
X5      rrsrwflgaclaavpragparlspaagywvlglwngceyfvlaphrvaltlrvpprrlgv    382
X6      ------------------------------------------------------------    344
X7      ------------------------------------------------------------    330
X8      rrsrwflgaclaavpragparlspaagywvlglwngceyfvlaphrvaltlrvpprrlgv    192
X9      rrsrwflgaclaavpragparlspaagywvlglwngceyfvlaphrvaltlrvpprrlgv    185

V1      fldyeagelsffnvsdgshiftfhdtfsgalcayfrprahdggehpdplticplpvrgtg    511
V2      ------------------------------------------------------------    344
X1      fldyeagelsffnvsdgshiftfhdtfsgalcayfrprahdggehpdplticplpvrgtg    540
X2      fldyeagelsffnvsdgshiftfhdtfsgalcayfrprahdggehpdplticplpvrgtg    504
X3      fldyeagelsffnvsdgshiftfhdtfsgalcayfrprahdggehpdplticplpvrgtg    504
X4      fldyeagelsffnvsdgshiftfhdtfsgalcayfrprahdggehpdplticplpvrgtg    497
X5      fldyeagelsffnvsdgshiftfhdtfsgalcayfrprahdggehpdplticplpvrgtg    442
X6      ------------------------------------------------------------    344
X7      ------------------------------------------------------------    330
X8      fldyeagelsffnvsdgshiftfhdtfsgalcayfrprahdggehpdplticplpvrgtg    252
X9      fldyeagelsffnvsdgshiftfhdtfsgalcayfrprahdggehpdplticplpvrgtg    245

V1      vpeendsdtwlqpyepadpaldww    535
V2      ------------------------    344
X1      vpeendsdtwlqpyepadpaldww    564
X2      vpeendsdtwlqpyepadpaldww    528
X3      vpeendsdtwlqpyepadpaldww    528
X4      vpeendsdtwlqpyepadpaldww    521
X5      vpeendsdtwlqpyepadpaldww    466
X6      ------------------------    344
X7      ------------------------    330
X8      vpeendsdtwlqpyepadpaldww    276
X9      vpeendsdtwlqpyepadpaldww    269
```

Nuclear localization sequence

Figure S1. Conserved domain analysis identifies a statistically significant non-canonical bZIP-like region within BTNL9. **(A)** NCBI CDD analysis of the human BTNL9 protein (NP_689760) revealed three conserved structural modules: an N-terminal IgV-MOG–like domain (residues 39–151; E = 3.4×10^{-60}), a central region containing a predicted bZIP_GCN4-like motif (residues 281–317; E = 3.5×10^{-3}), and a C-terminal SPRY/PRY domain (residues 329–503; E = 3.9×10^{-89}). The putative bZIP-like region overlaps a heptad-repeat leucine zipper pattern and a cluster of basic residues, consistent with DNA-binding/dimerization features found in bZIP proteins. Although non-canonical in sequence composition, the statistically significant match supports the presence of a bona fide bZIP-like structural element within BTNL9. **(B)** Coiled-coil prediction analysis using Marcoil (left) and Multicoil2 (right) shows a single high-probability coiled-coil region in BTNL9 spanning amino acids 274–315. Both algorithms identify a sharp peak exceeding the coiled-coil probability threshold (red shading), consistent with a predicted leucine-zipper–like segment. The predicted Single α-Helix (SAH) score (blue dashed line) remains below the SAH threshold, supporting a structured coiled-coil rather than an extended SAH conformation. **(C)** Marcoil analysis identifies a single, high-probability coiled-coil region within BTNL9. The heptad repeat pattern (abcdefg) is mapped onto the corresponding amino acid sequence, with the predicted coiled-coil segment highlighted. This region spans residues 274–315, consistent with a canonical coiled-coil structural motif. **(D)** Schematic representation of BTNL9 ΔbZIP and bZIP-mutant constructs. The upper panel shows the primer positions used to generate the BTNL9 ΔbZIP construct, with the engineered junction indicated as the "ΔbZIP junction. The lower panel illustrates the bZIP-mutant construct, indicating the specific point mutations introduced within the bZIP domain together with the corresponding primer positions. These schematics outline the design strategy for generating BTNL9 variants used in functional assays. **(E)** Full-length protein sequence of BTNL9 was analyzed using NLStradamus, a program to predict potential NLS domain. Short sequence RKQRRSREKLRKQAEKR was identified as NLS, with threshold score above 0.6. **(F)** Protein sequence alignment of all available BTNL9 variants from NCBI revealed a conserved NLS domain except for variant X8 and X9.

Figure S2

A

BTNL9-rep-1

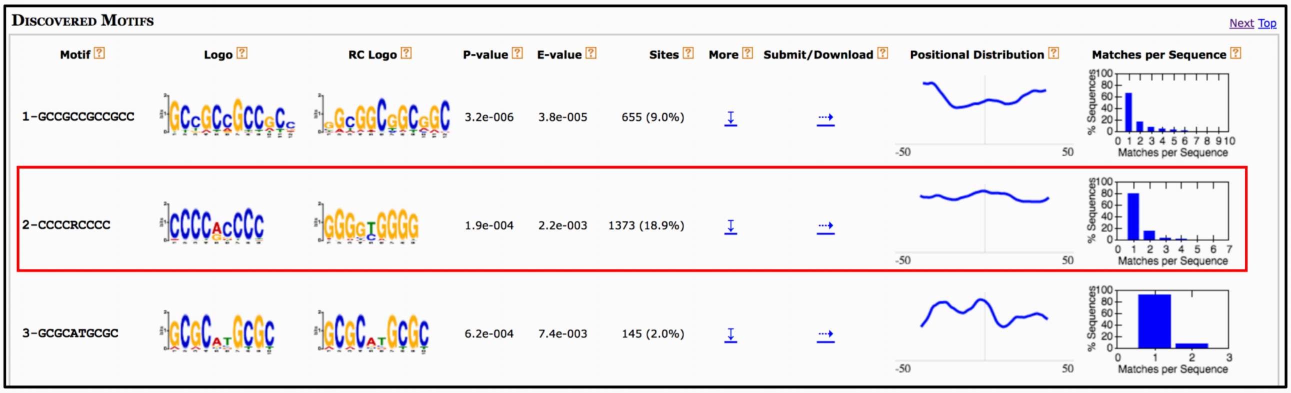

BTNL9-rep-2

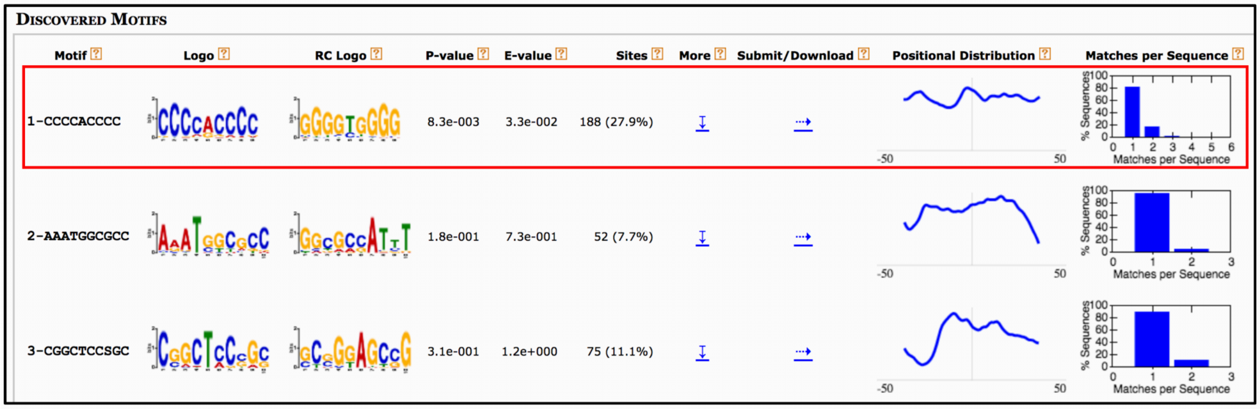

B

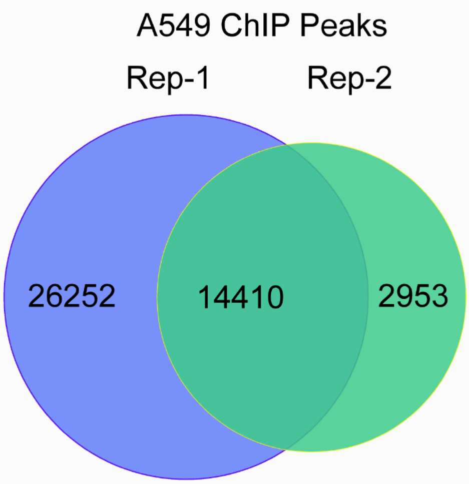

C

Metric	Value
Rep-1 total peaks	40,662
Rep-2 total peaks	17,363
Overlapping peaks	14,410
Rep-1 overlapping Rep-2	35.40%
Rep-2 overlapping Rep-1	86.10%
Final promoter-associated peaks	9,709

Figure S2

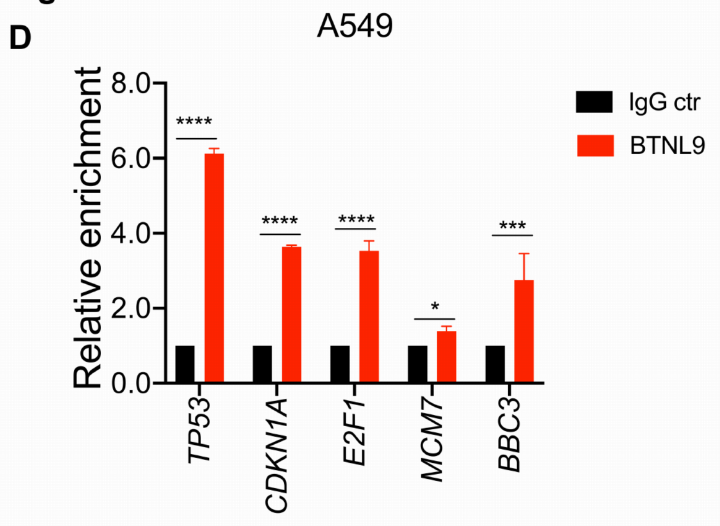
D
A549
Relative enrichment
8.0
6.0
4.0
2.0
0.0
****
****
****
*
***
TP53
CDKN1A
E2F1
MCM7
BBC3
IgG ctr
BTNL9

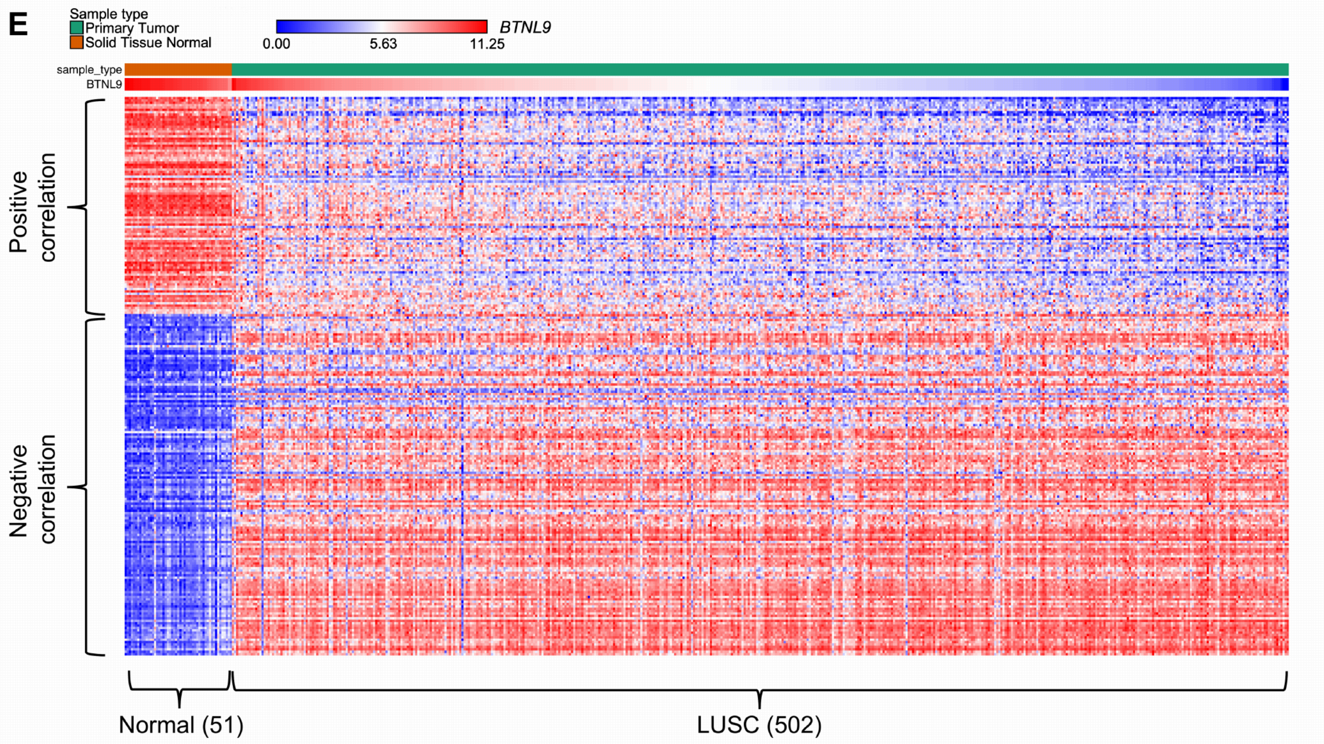
E
Sample type
Primary Tumor
Solid Tissue Normal
BTNL9
0.00
5.63
11.25
sample_type
BTNL9
Positive correlation
Negative correlation
Normal (51)
LUSC (502)

Figure S2. Potential target genes and transcription factors of BTNL9 and its binding regions. **(A)** MEME-ChIP DNA motif analyses of both BTNL9 ChIP-seq replicates revealed a potential DNA binding conserved motif sequence. The red box highlights the consistent presence of the same motif CCCCRCCCC in both sets of ChIP-seq data. **(B)** Venn diagram showing overlap of BTNL9 ChIP-seq peaks between biological replicates. Peak sets were independently filtered from Rep-1 (40,662 peaks) and Rep-2 (17,363 peaks) using identical thresholds ($p < 1.0 \times 10^{-5}$, FDR < 0.005). The asymmetry in peak numbers reflects the difference in sequencing depth between replicates (Rep-1: 70.4M reads; Rep-2: 47.4M reads. **(C)** Summary statistics of inter-replicate peak reproducibility. A total of 14,410 peaks overlapped between replicates, with 86.1% of Rep-2 peaks confirmed in Rep-1, demonstrating strong inter-replicate concordance. The 9,709 promoter-proximal peaks were derived from the pooled replicate peak set used for downstream analyses. **(D)** Bar graphs representing the ChIP-qPCR validation of chromatin enrichment for 4 genes (*TP53*, *CDKN1A*, *E2F1, MCM7* and *BBC3*), as identified in ChIP-seq data. Results were presented fold enrichment method calculation. Significance was determined by a two-tailed Student's *t*-test: * $p < 0.05$, *** $p < 0.001$ and **** $p < 0.0001$. **(E)** Heatmap illustrating expression profiles of 46 genes significantly correlated to BTNL9 mRNA levels from TCGA LUSC dataset (Spearman's correlation coefficient $r \leq -0.45$ or $r \geq 0.45$).

Figure S3

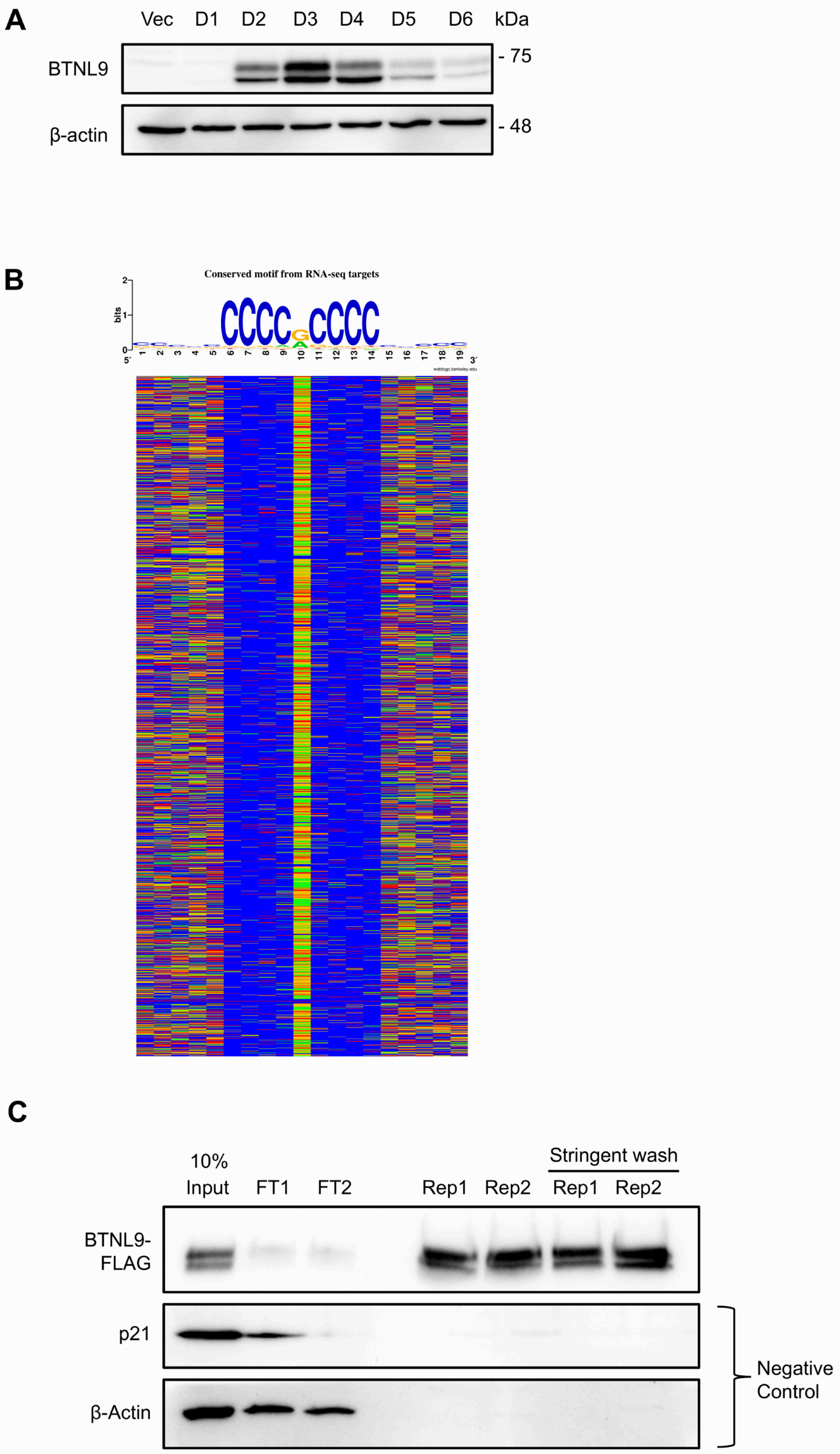
A
Vec
D1
D2
D3
D4
D5
D6
kDa
BTNL9
- 75
β-actin
- 48
B
Conserved motif from RNA-seq targets
bits
C
10%
Input
FT1
FT2
Rep1
Rep2
Stringent wash
Rep1
Rep2
BTNL9-
FLAG
p21
β-Actin
Negative
Control

Figure S3. BTNL9 expression and motif-dependent promoter interaction. **(A)** Western blot showing the temporal expression pattern of BTNL9 in A549 that were infected with either a empty vector control or BTNL9 lentivirus and harvested from Day 1 to Day 6, using β-actin as a loading control. **(B)** Sequence logo (top) and heatmap (bottom) representing conserved nucleotide motifs at promoter regions of genes co-identified by BTNL9 ChIP-seq and RNA-seq in A549 cells. The sequence logo illustrates an 9-nucleotide consensus motif enriched across BTNL9-bound promoters, with high conservation of cytosine and guanine residues. The heatmap displays aligned promoter sequences from overlapping BTNL9 targets, highlighting positional conservation and homology at putative BTNL9 binding sites. These data suggest a shared cis-regulatory element among BTNL9-regulated genes. **(C)** A 235 bp biotinylated fragment of the E2F1 promoter containing the predicted BTNL9 binding motif was incubated with nuclear extracts from A549 cells overexpressing FLAG-tagged BTNL9. DANN-protein complexes were captured using streptavidin-coated magnetic beads and analyzed by immunoblotting. "Input" represents 10% of the nuclear extract prior to pulldown, while FT1 and FT2 denote flow-through fractions collected after binding. BTNL9 was efficiently enriched by the DNA probe and remained strongly detectable following stringent washing (0.2% Tween-20; Rep1, Rep2), indicating a stable association with the promoter fragment. p21 and β-actin served as negative controls and were absent from pulldown fractions, confirming the specificity of the interaction

Figure S4

A

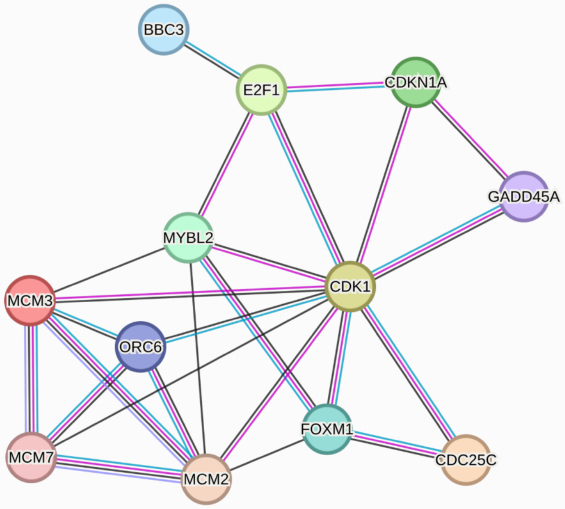
BBC3
E2F1
CDKN1A
GADD45A
MYBL2
CDK1
MCM3
ORC6
FOXM1
CDC25C
MCM7
MCM2

B

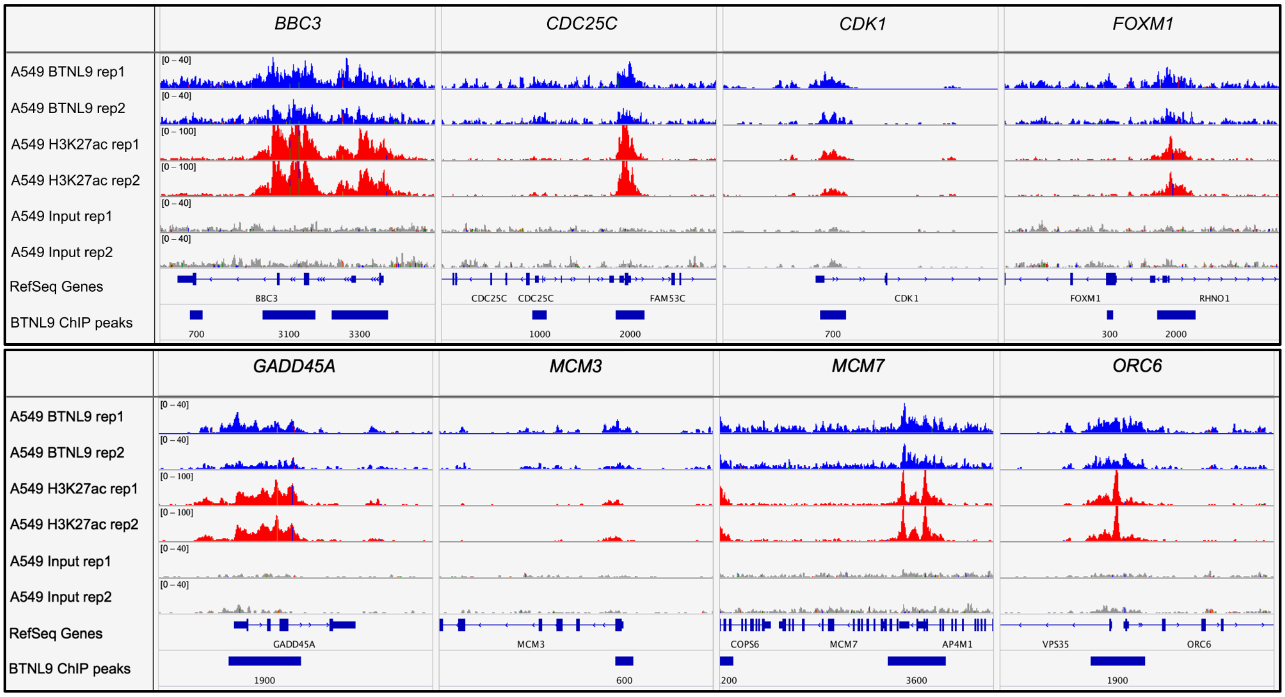
BBC3
CDC25C
CDK1
FOXM1
GADD45A
MCM3
MCM7
ORC6
A549 BTNL9 rep1
A549 BTNL9 rep2
A549 H3K27ac rep1
A549 H3K27ac rep2
A549 Input rep1
A549 Input rep2
RefSeq Genes
BTNL9 ChIP peaks
[0 – 40]
[0 – 100]
BBC3
CDC25C
CDC25C
FAM53C
CDK1
FOXM1
RHNO1
GADD45A
MCM3
COPS6
MCM7
AP4M1
VPS35
ORC6
700
3100
3300
1000
2000
700
300
2000
1900
600
200
3600
1900

Figure S4

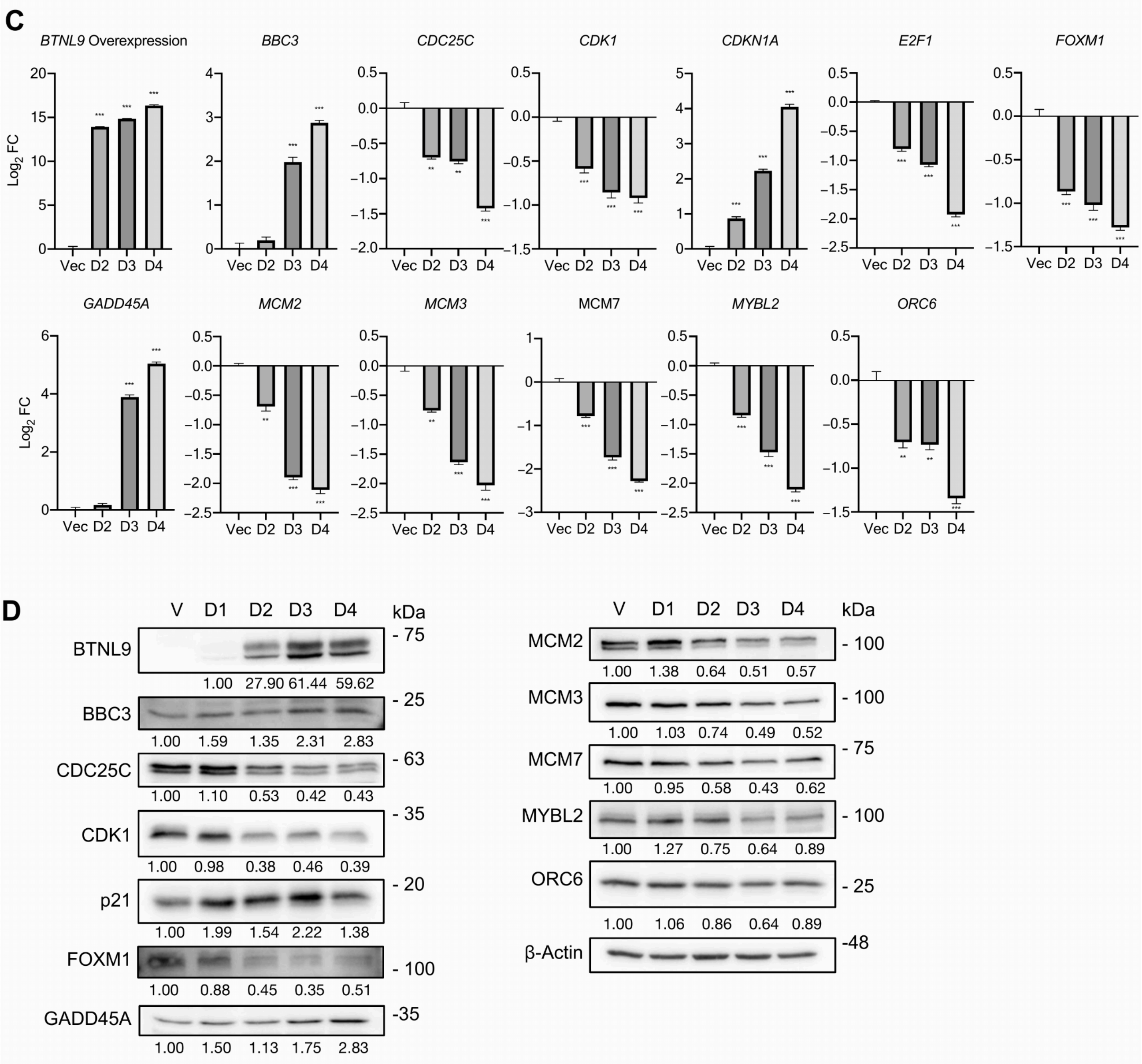
C
BTNL9 Overexpression
BBC3
CDC25C
CDK1
CDKN1A
E2F1
FOXM1
GADD45A
MCM2
MCM3
MCM7
MYBL2
ORC6
Log2 FC
Vec D2 D3 D4
D
V D1 D2 D3 D4 kDa
BTNL9 - 75
1.00 27.90 61.44 59.62
BBC3 - 25
1.00 1.59 1.35 2.31 2.83
CDC25C - 63
1.00 1.10 0.53 0.42 0.43
CDK1 - 35
1.00 0.98 0.38 0.46 0.39
p21 - 20
1.00 1.99 1.54 2.22 1.38
FOXM1 - 100
1.00 0.88 0.45 0.35 0.51
GADD45A -35
1.00 1.50 1.13 1.75 2.83
MCM2 - 100
1.00 1.38 0.64 0.51 0.57
MCM3 - 100
1.00 1.03 0.74 0.49 0.52
MCM7 - 75
1.00 0.95 0.58 0.43 0.62
MYBL2 - 100
1.00 1.27 0.75 0.64 0.89
ORC6 - 25
1.00 1.06 0.86 0.64 0.89
β-Actin -48

Figure S4

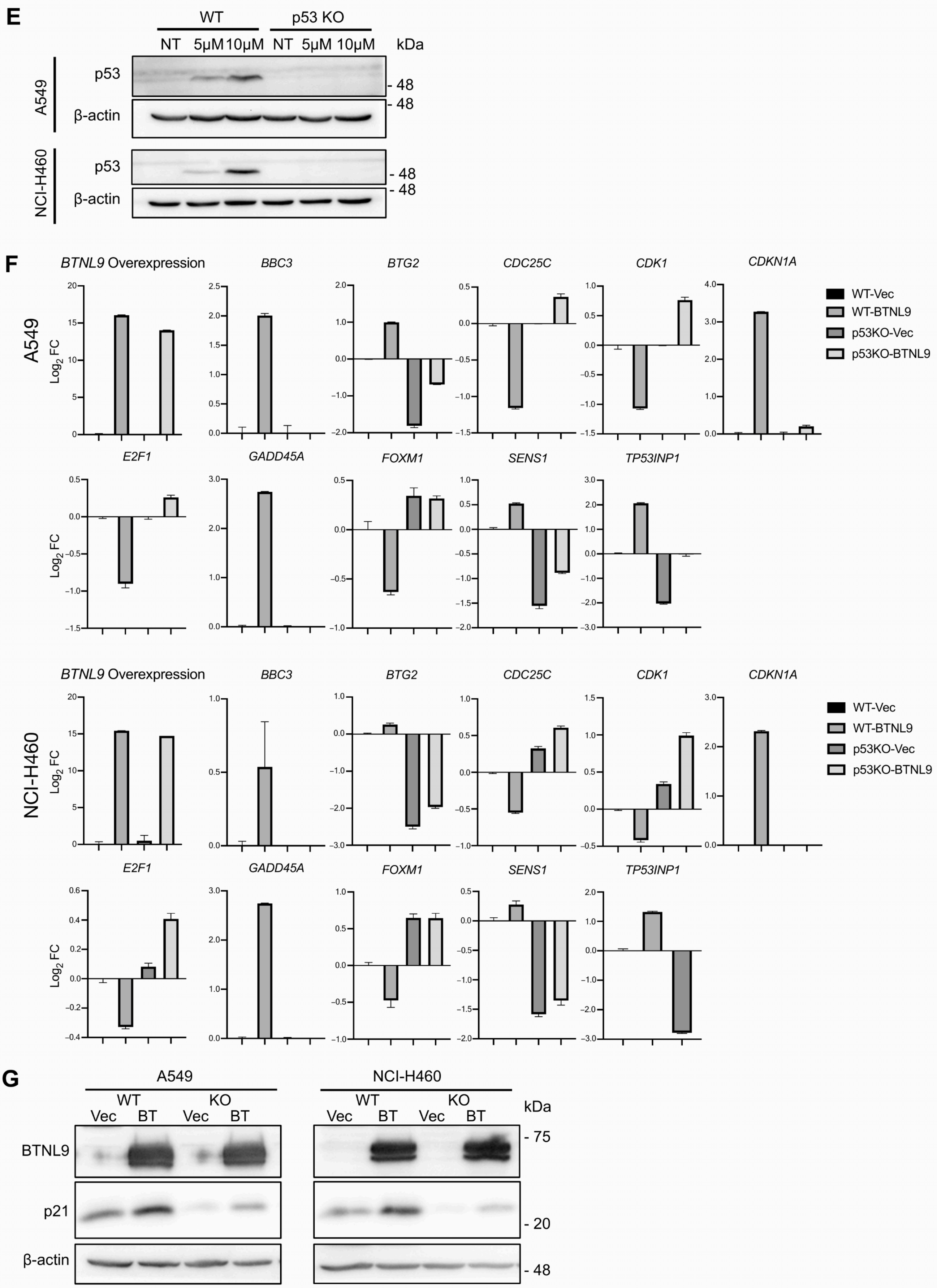
E
WT
p53 KO
NT 5μM 10μM NT 5μM 10μM
kDa
A549
p53
β-actin
- 48
- 48
NCI-H460
p53
β-actin
- 48
- 48
F
BTNL9 Overexpression
BBC3
BTG2
CDC25C
CDK1
CDKN1A
E2F1
GADD45A
FOXM1
SENS1
TP53INP1
A549
NCI-H460
Log2 FC
WT-Vec
WT-BTNL9
p53KO-Vec
p53KO-BTNL9
G
A549
NCI-H460
WT
KO
Vec BT Vec BT
kDa
BTNL9
p21
β-actin
- 75
- 20
- 48

Figure S4. Analysis of BTNL9 RNA-seq data obtained in NCI-H460 cell line. **(A)** String analysis showing interconnection between the selected 12 genes from three clusters identified in RNA-seq data. **(B)** Integrative Genomics Viewer (IGV) plot showcasing ChIP-seq peaks of BTNL9, H3K27ac, and input controls at the loci of *BBC3*, *CDC25C*, *CDK1*. *FOXM1*, *GADD45A*, *MCM3*, *MCM7* and *ORC6*. **(C)** Bar graphs representing qRT-PCR analyses in NCI-H460 cells overexpressing either an empty vector control or BTNL9, with samples harvested on the indicated day. All values were calculated as mean $\log_2$ FC ±SD. Significance was determined by a two-tailed Student's *t*-test: * $p < 0.05$, ** $p < 0.01$ and *** $p < 0.001$. All experiments were conducted in triplicates. Primers for qRT-PCR of tested genes were listed in Table S8. **(D)** Representative western blot for selected genes in NCI-H460 cells overexpressing either an empty vector control or BTNL9, with samples harvested on the indicated day. β-actin was used as a loading control. Signal intensities were quantified with iBright Analysis software and semi-quantitative protein expression levels were calculated by normalizing to β-actin. E2F1 was not included in the western blot results as its signal was underdetectable. **(E)** Western blot analysis of p53 expression in wild-type (WT) and p53 knockout (p53 KO) A549 and NCI-H460 cell lines. Cells were treated with Nutlin-3 at 5 μM and 10 μM for 24 hours. β-actin was used as a housekeeping control. **(F)** mRNA expression levels of BTNL9 downstream target genes (*BBC3*, *BTG2*, *CDC25C*, *CDK1*, *CDKN1A*, *E2F1*, *GADD45A*, *FOXM1*, *SENS1* and *TP53INP1*) were assessed in WT and p53 KO A549 and NCI-H460 cells using RT-qPCR. Primers for qRT-PCR were listed in Table S9. **(G)** BTNL9 induces p21 protein in a p53-dependent manner. Western blot of BTNL9, p21, and β-Actin in A549 and NCI-H460 cells under wild-type (WT) and p53 knockout (KO) backgrounds, transfected with empty vector control (Vec) or BTNL9 (BT). BTNL9 overexpression was confirmed in BT lanes. p21 protein increased in WT-BT A549 cells compared to WT-Vec, but this effect was absent in p53 KO cells. β-Actin served as a loading control. For (C) and (F) data represents the mean of biological triplicates and tested by Student's *t* test (unpaired two-tailed). * $p < 0.05$, ** $p < 0.01$ and *** $p < 0.001$.

Figure S5

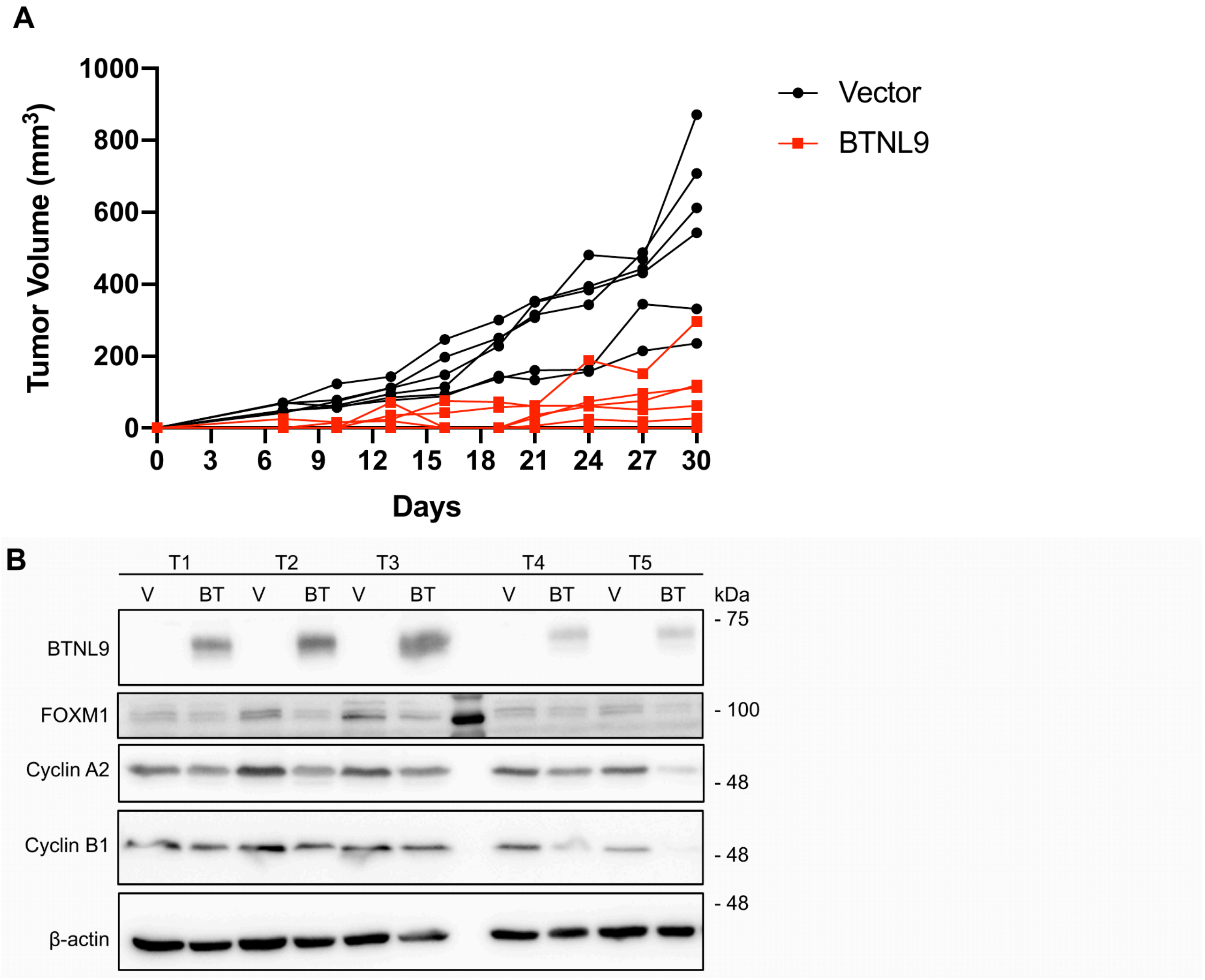

Figure S5. BTNL9 overexpression suppresses cell cycle protein expression in A549 xenograft tumors. **(A)** Tumor volumes were recorded every 3 days until Day 30 for each mouse in the indicated treatment groups. Data are presented as individual growth curves to illustrate variability within groups and the time point at which differences between treatments became evident. **(B)** Western blot analysis of tumor lysates from A549 xenografts transduced with either vector control or BTNL9 overexpression constructs. Protein expression of key cell cycle regulators was examined alongside β-actin as a loading control. Compared to vector control tumors, BTNL9-overexpressing tumors displayed suppresses levels of selected cell cycle proteins, consistent with transcriptional repression observed in vitro. These results provide in vivo evidence that BTNL9 modulates cell cycle progression at the protein level.

Figure S6

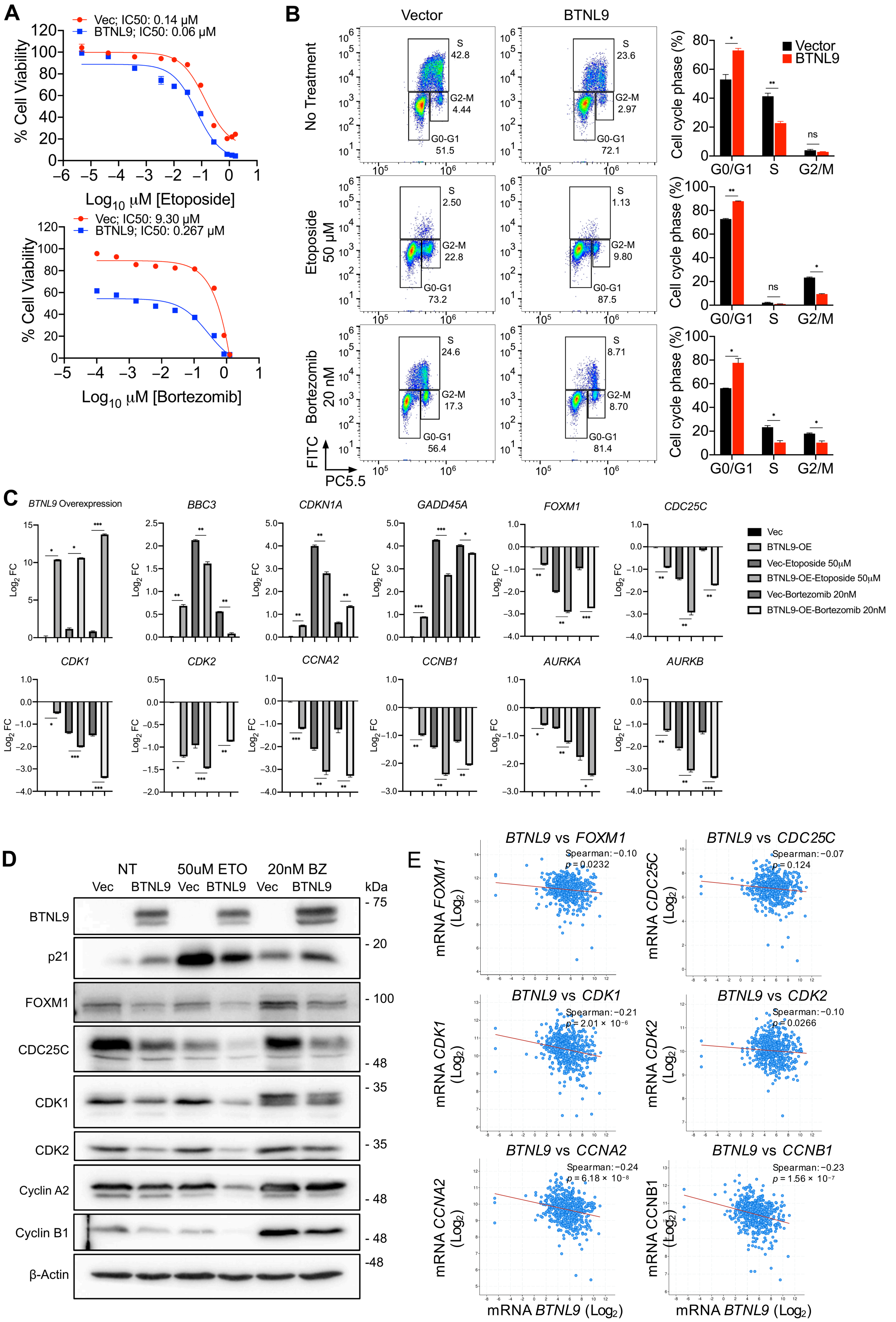
A
Vec; IC50: 0.14 μM
BTNL9; IC50: 0.06 μM
% Cell Viability
Log10 μM [Etoposide]
Vec; IC50: 9.30 μM
BTNL9; IC50: 0.267 μM
Log10 μM [Bortezomib]
B
Vector
BTNL9
No Treatment
Etoposide 50 μM
Bortezomib 20 nM
FITC
PC5.5
S
G2-M
G0-G1
Cell cycle phase (%)
G0/G1
S
G2/M
ns
C
BTNL9 Overexpression
BBC3
CDKN1A
GADD45A
FOXM1
CDC25C
CDK1
CDK2
CCNA2
CCNB1
AURKA
AURKB
Log2 FC
Vec
BTNL9-OE
Vec-Etoposide 50μM
BTNL9-OE-Etoposide 50μM
Vec-Bortezomib 20nM
BTNL9-OE-Bortezomib 20nM
D
NT
50uM ETO
20nM BZ
Vec
BTNL9
kDa
BTNL9
p21
FOXM1
CDC25C
CDK1
CDK2
Cyclin A2
Cyclin B1
β-Actin
E
BTNL9 vs FOXM1
Spearman: −0.10
p = 0.0232
BTNL9 vs CDC25C
Spearman: −0.07
p = 0.124
BTNL9 vs CDK1
Spearman: −0.21
p = 2.01 × 10−6
BTNL9 vs CDK2
Spearman: −0.10
p = 0.0266
BTNL9 vs CCNA2
Spearman: −0.24
p = 6.18 × 10−8
BTNL9 vs CCNB1
Spearman: −0.23
p = 1.56 × 10−7
mRNA BTNL9 (Log2)

Figure S6. BTNL9 enhances drug sensitivity to etoposide and bortezomib in lung cancer cells by regulating cell cycle pathways in NCI-H460. **(A)** Dose-response curves from cytotoxicity assays of etoposide and bortezomib treatments for 48 hr in NCI-H460 cells overexpressing BTNL9 or vector control was used to estimate for IC50 value. **(B)** Representative scatter plots for the cell cycle assay, performed using BrdU staining on vector control and BTNL9-overexpressing in NCI-H460 cells. Cell populations representing G0/G1 phase, S phase and G2/M phase were gated. Bar graphs representing the compilation of individual experimental results with mean ± SD. **(C)** Bar graphs representing RT-qPCR analyses in NCI-H460 cells overexpressing either empty vector control or BTNL9. All values were calculated as mean $\log_2$ FC ±SD. Significance was determined by a two-tailed Student's *t*-test: * $p < 0.05$, ** $p < 0.01$ and *** $p < 0.001$. All experiments were conducted in biological triplicates. Primers for qRT-PCR of tested genes were listed in Table S9. **(D)** Representative western blot of selected key cell cycle markers in NCI-H460 cells overexpressing either empty vector control or BTNL9. β-actin was used as a loading control. **(E)** Correlation analysis between BTNL9 mRNA levels and the expression of cell cycle-related genes (*FOXM1*, *CDC25C*, *CDK1*, *CDK2*, *CCNA2*, and *CCNB1*) in LUSC patient samples derived from TCGA dataset. Spearman correlation coefficients and p-values are shown, part of the cell cycle markers indicating a negative correlation with BTNL9 expression. For (B), (C), and (D), cells were either with no treatment (NT), or treated with 50 μM etoposide or 20 nM bortezomib for 18 hr. For (B) and (C) data represents the mean of biological triplicates and tested by Student's *t* test (unpaired two-tailed). * $p < 0.05$, ** $p < 0.01$ and *** $p < 0.001$.

Figure S7

A

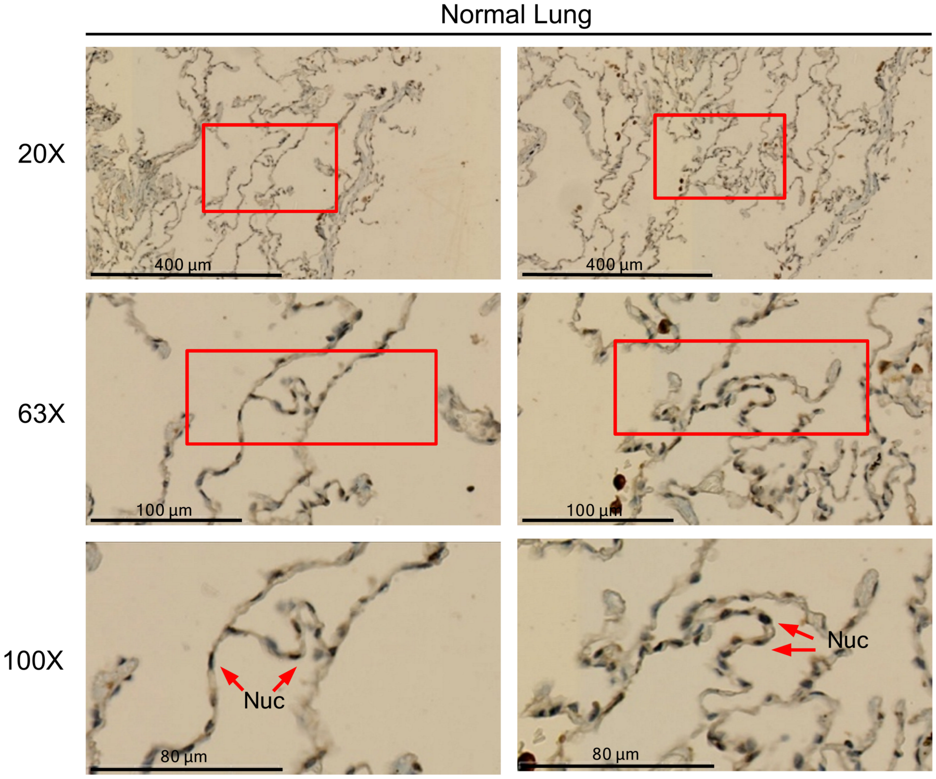
Normal Lung
20X
63X
100X
400 µm
400 µm
100 µm
100 µm
80 µm
80 µm
Nuc
Nuc

B

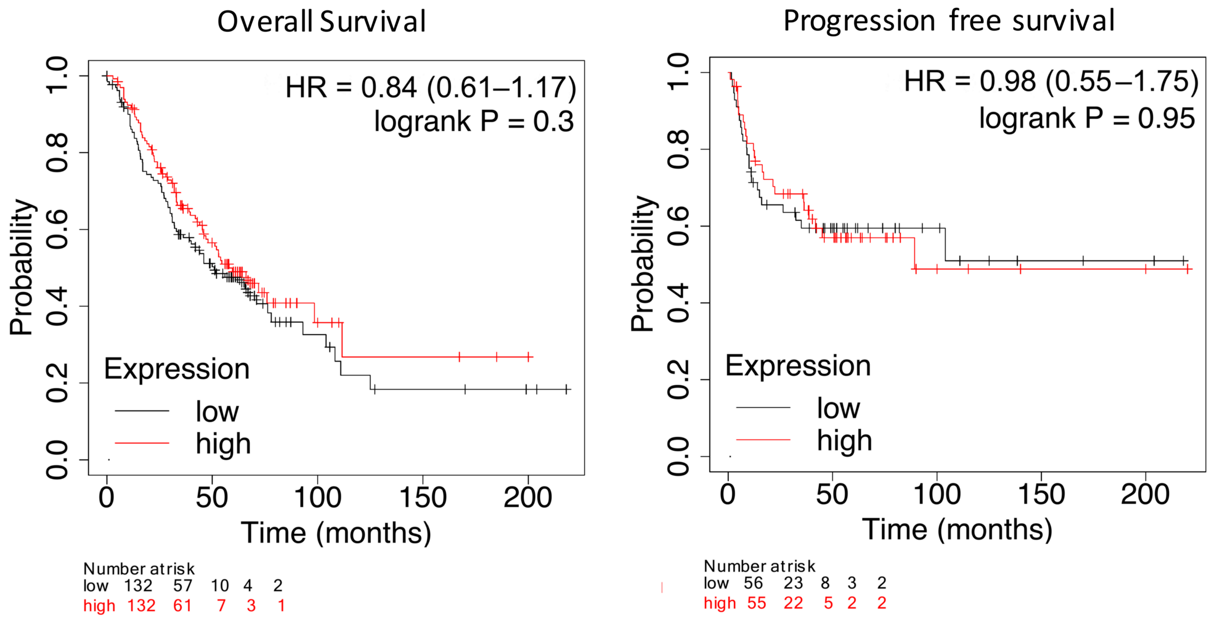
Overall Survival
HR = 0.84 (0.61–1.17)
logrank P = 0.3
Probability
Time (months)
Expression
low
high
Number at risk
low 132 57 10 4 2
high 132 61 7 3 1
Progression free survival
HR = 0.98 (0.55–1.75)
logrank P = 0.95
Probability
Time (months)
Expression
low
high
Number at risk
low 56 23 8 3 2
high 55 22 5 2 2

Figure S7

C

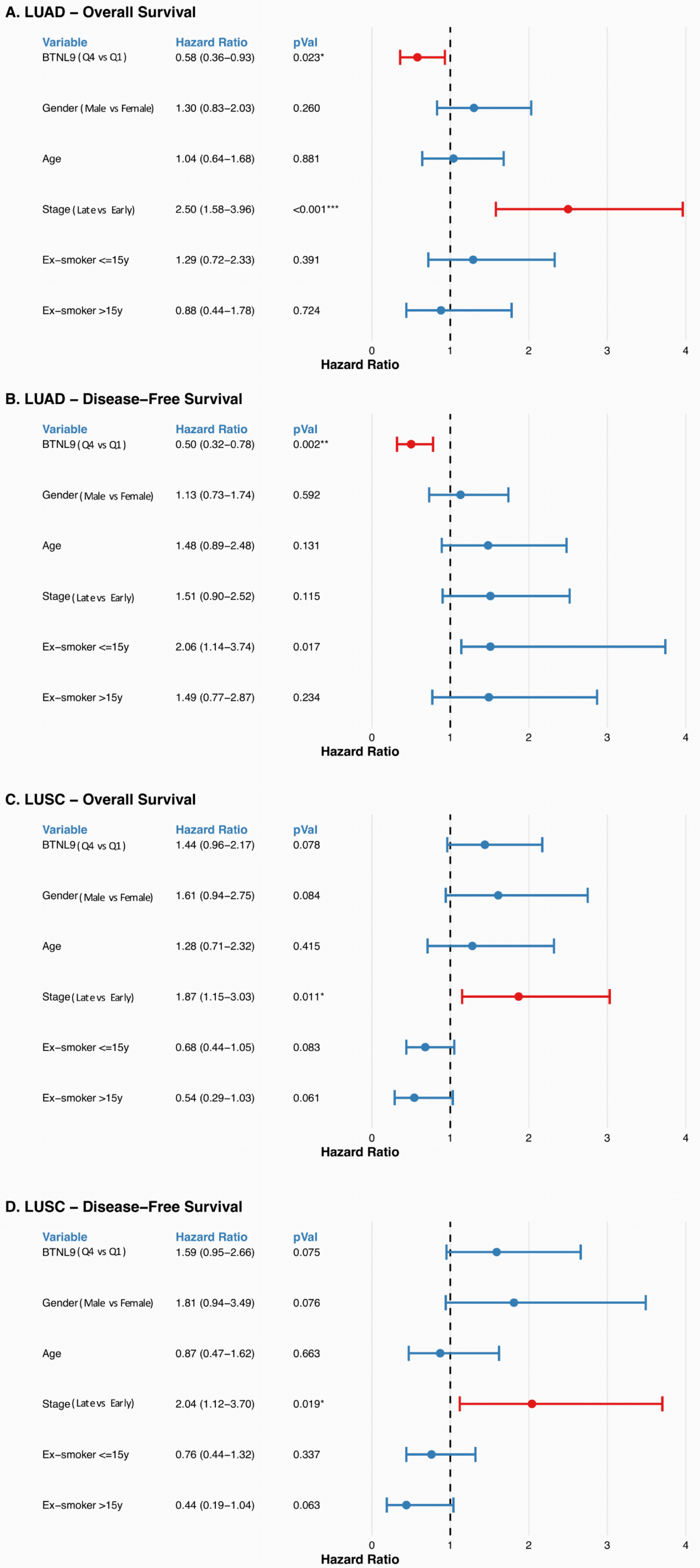
A. LUAD – Overall Survival
Variable Hazard Ratio pVal
BTNL9 (Q4 vs Q1) 0.58 (0.36–0.93) 0.023*
Gender (Male vs Female) 1.30 (0.83–2.03) 0.260
Age 1.04 (0.64–1.68) 0.881
Stage (Late vs Early) 2.50 (1.58–3.96) <0.001***
Ex–smoker <=15y 1.29 (0.72–2.33) 0.391
Ex–smoker >15y 0.88 (0.44–1.78) 0.724
0 1 2 3 4
Hazard Ratio
B. LUAD – Disease–Free Survival
Variable Hazard Ratio pVal
BTNL9 (Q4 vs Q1) 0.50 (0.32–0.78) 0.002**
Gender (Male vs Female) 1.13 (0.73–1.74) 0.592
Age 1.48 (0.89–2.48) 0.131
Stage (Late vs Early) 1.51 (0.90–2.52) 0.115
Ex–smoker <=15y 2.06 (1.14–3.74) 0.017
Ex–smoker >15y 1.49 (0.77–2.87) 0.234
0 1 2 3 4
Hazard Ratio
C. LUSC – Overall Survival
Variable Hazard Ratio pVal
BTNL9 (Q4 vs Q1) 1.44 (0.96–2.17) 0.078
Gender (Male vs Female) 1.61 (0.94–2.75) 0.084
Age 1.28 (0.71–2.32) 0.415
Stage (Late vs Early) 1.87 (1.15–3.03) 0.011*
Ex–smoker <=15y 0.68 (0.44–1.05) 0.083
Ex–smoker >15y 0.54 (0.29–1.03) 0.061
0 1 2 3 4
Hazard Ratio
D. LUSC – Disease–Free Survival
Variable Hazard Ratio pVal
BTNL9 (Q4 vs Q1) 1.59 (0.95–2.66) 0.075
Gender (Male vs Female) 1.81 (0.94–3.49) 0.076
Age 0.87 (0.47–1.62) 0.663
Stage (Late vs Early) 2.04 (1.12–3.70) 0.019*
Ex–smoker <=15y 0.76 (0.44–1.32) 0.337
Ex–smoker >15y 0.44 (0.19–1.04) 0.063
0 1 2 3 4
Hazard Ratio

Figure S7

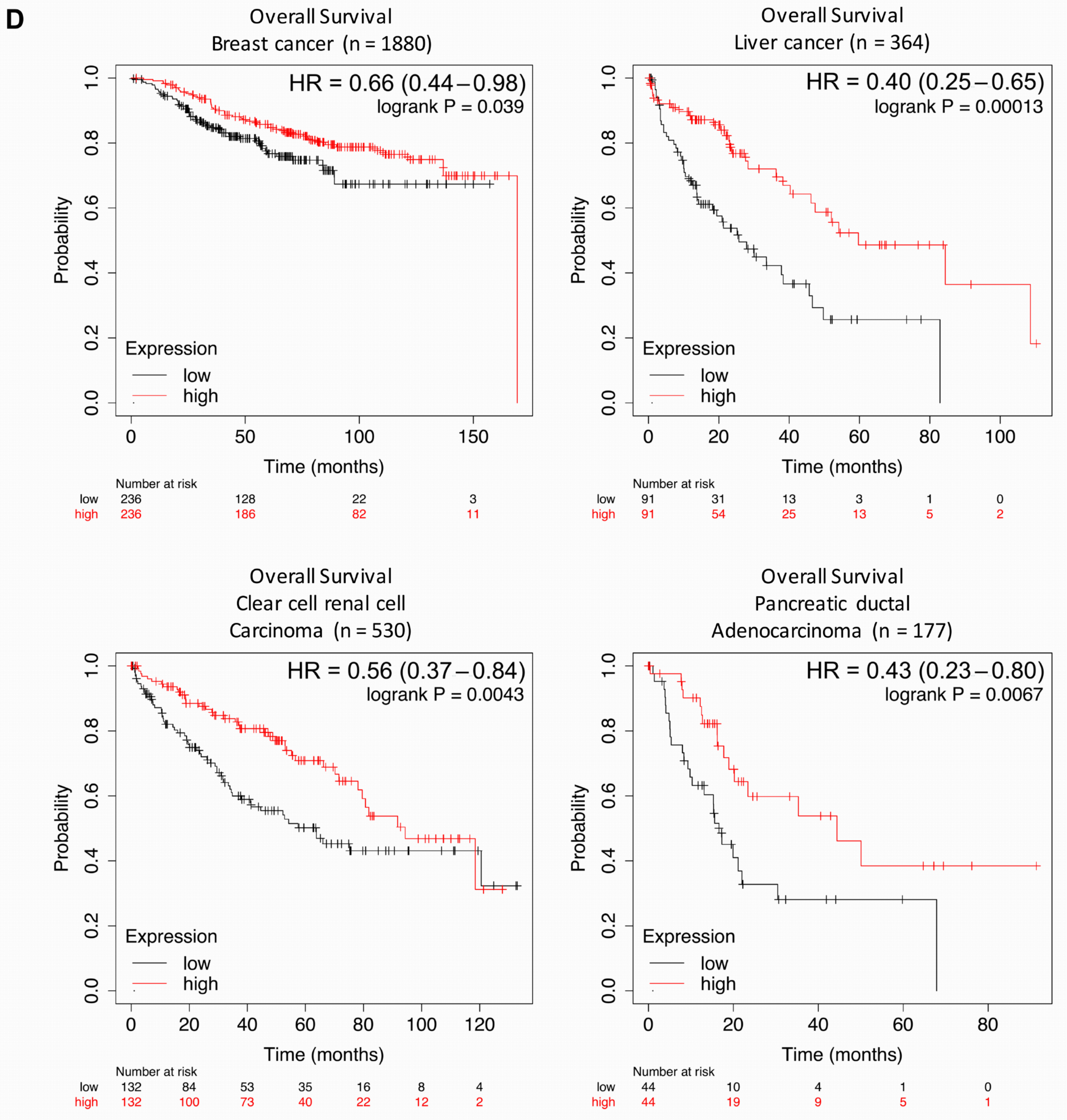
D
Overall Survival
Breast cancer (n = 1880)
HR = 0.66 (0.44 – 0.98)
logrank P = 0.039
Probability
Expression
low
high
Time (months)
Number at risk
low 236 128 22 3
high 236 186 82 11
Overall Survival
Liver cancer (n = 364)
HR = 0.40 (0.25 – 0.65)
logrank P = 0.00013
Number at risk
low 91 31 13 3 1 0
high 91 54 25 13 5 2
Overall Survival
Clear cell renal cell
Carcinoma (n = 530)
HR = 0.56 (0.37 – 0.84)
logrank P = 0.0043
Number at risk
low 132 84 53 35 16 8 4
high 132 100 73 40 22 12 2
Overall Survival
Pancreatic ductal
Adenocarcinoma (n = 177)
HR = 0.43 (0.23 – 0.80)
logrank P = 0.0067
Number at risk
low 44 10 4 1 0
high 44 19 9 5 1

Figure S7

E

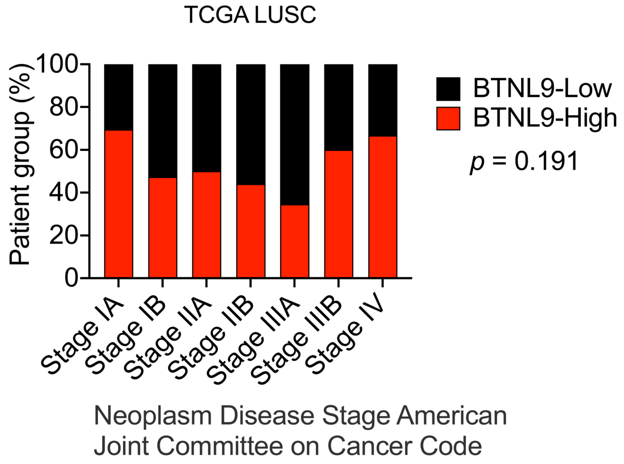

F

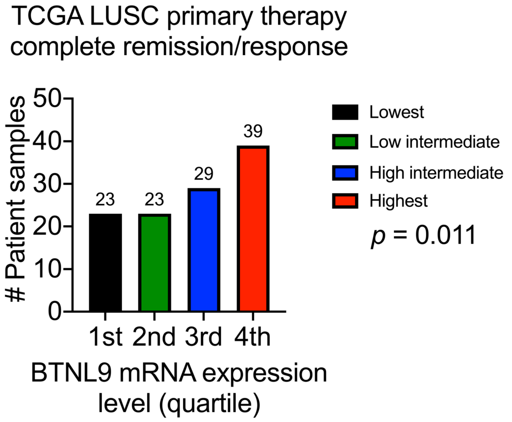

G

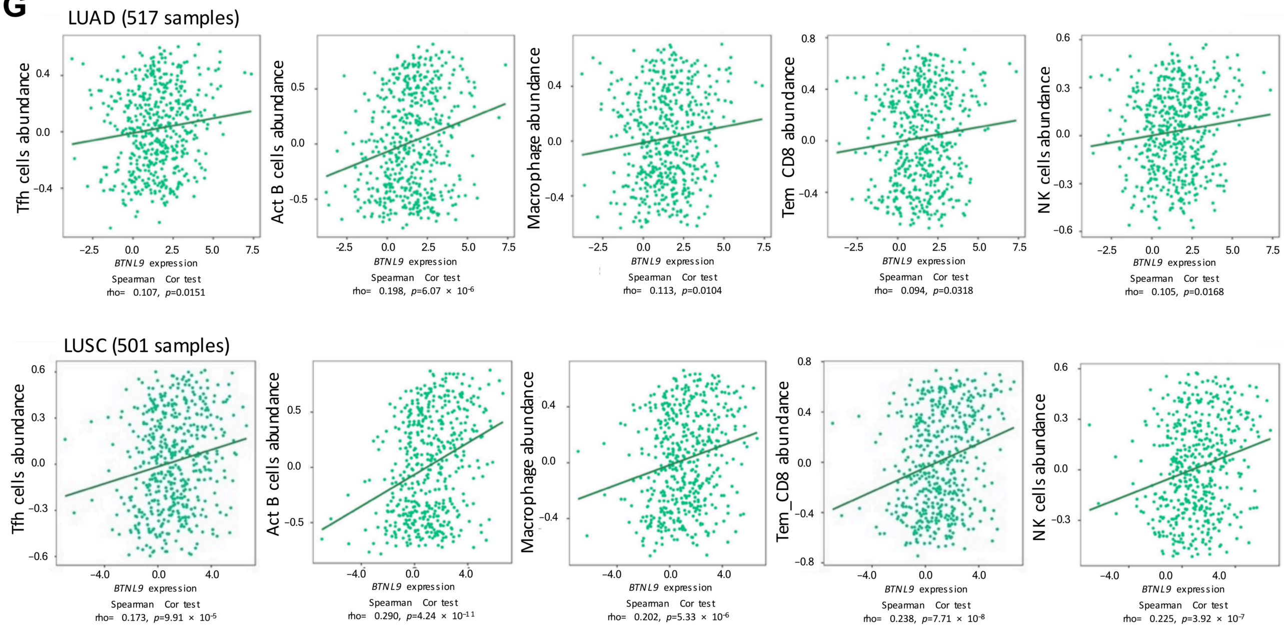

H

```
            240         250         260               270         280         290
                                                                  Human bZIP domain
hBTNL9  LVVQIADVFVPGASAWKSAFVATL---PL-LLVLAALALGVLRKQRRSREKLRKQAEKRQ
        .:.:::::::.:  : ::.:::.::    :: :.::. :::   . : :   .::   ::.:. .
mBtnl9  FVIQIADVFLPRMSPWKKAFVGTLVVLPLSLIVLTMLALRYFYKLRSFQEKQVKQGEEVR

            300         310         320         330         340         350
        Human bZIP domain
hBTNL9  EKLTAELEKLQTELDWRRAEGQAEWRAAQKYAVDVTLDPASAHPSLEVSEDGKSVSSRGA
        :::         ::::::::.::::::::::.::.::::::::.::::::::::.::.:::::  .
mBtnl9  EKL-------QTELDWRRSEGQAEWRAAQQYAADVTLDPATAHPSLEVSNNGKTVSSRLG
```

Figure S7. Multifaceted impact of BTNL9 on the progression and prognosis of lung cancer. **(A)** IHC staining on normal lung tissue samples from a tissue microarray. Images were captured at 20X and 63X magnifications, with areas inside of box further magnified to X100. Red arrows highlight pneumocytes exhibiting nuclear-localized BTNL9 expression. Kaplan–Meier curve analysis comparing overall survival and progression-free survival in **(B)** LUSC patients within the first and fourth quartiles of BTNL9 expression. *p*-value was obtained from the log-rank test. **(C)** Multivariate Cox regression analysis of BTNL9 expression and clinical covariates in LUAD and LUSC. Forest plots show hazard ratios (HR) and 95% confidence intervals (CI) for overall survival (OS) and disease-free survival (DFS) in LUAD (A–B) and LUSC (C–D). BTNL9 expression was stratified into quartiles; Q4 (highest) vs Q1 (lowest) was used for comparison. Models were adjusted for gender (male vs female), age, tumor stage (late vs early), and smoking history. Asterisks indicate statistical significance (* $p < 0.05$, ** $p < 0.01$ and *** $p < 0.001$). Vertical dashed lines represent HR = 1.0. **(D)** Kaplan–Meier curve analysis comparing overall survival in cancers other than lung cancer, specifically breast cancer, liver cancer, clear cell renal cell carcinoma, and pancreatic ductal adenocarcinoma, within the first and fourth quartiles of BTNL9 expression. *p*-value was determined by the log-rank test. **(E)** Bar graph showing the distribution of LUSC patients within the lowest (1st quartile) and highest (4th quartile) of BTNL9 expression according to tumor stage (American Joint Committee on Cancer, AJCC). Data were generated using the TCGA LUSC dataset. Statistical association was assessed by Chi-squared test (p=0.191) and Cochran-Armitage trend test (Z=-0.753, p=0.452), indicating no significant association between BTNL9 expression and tumor stage in LUSC. **(F)** Bar graph showing the number of LUSC patients with complete remission/response following primary treatment, distributed across the four quartiles of BTNL9 mRNA expression level. Data were generated using the TCGA LUSC dataset. Statistical significance of the trend across quartiles was assessed by Cochran-Armitage trend test (Z=2.539, p=0.011). **(G)** Correlation analysis of BTNL9 gene expression with immune cell infiltration based on TCGA LUAD and LUSC dataset. A positive correlation with *rho* > 0 and a negative correlation with *rho* < 0. Significant data points are represented by $p < 0.05$. Five immune cell populations were examined: Follicular helper T cells (Tfh), activated B cells, macrophages, effector CD8+ T cell, and natural killer (NK) cells. **(H)** Pairwise sequence alignment of human BTNL9 (hBTNL9) and mouse BTNL9 (mBtnl9) illustrating the conserved b2P domain (blue box). Amino acid position numbers of hBTNL9 are shown above the alignment.

Table S1
A549 ChIP-seq peak call data for BTNL9 revealed a list of 9,709 genes, identified through stringent criteria with $p < 1.0 \times 10^{-5}$ (MACS recommendations), and a false discovery rate (FDR) of $q < 0.005$. (This is a data file in Excel spreadsheet.)

Table S2
Genomic Regions Enrichment of Annotations Tool (GREAT) was used to annotate ChIP peaks to the nearest genes within ±1 kb of the transcription start site (TSS), identifying 9,709 genes associated with BTNL9 binding sites. (This is a data file in Excel spreadsheet.)

Table S3
Selected 5 genes for ChIP-qPCR validation

Gene name	Chr	Start	End	Length	Signal Value	Fold Change	p-Value	Benjamini-Hochberg FDR
TP53	chr17	7589100	7592500	3400	242.39	4.88	7.47×10^{-10}	1.95×10^{-6}
CDKN1A	chr6	36645000	36649100	4100	200.10	4.03	7.11×10^{-7}	2.35×10^{-4}
E2F1	chr20	32274600	32275000	400	205.62	4.14	5.21×10^{-8}	3.69×10^{-5}
MCM7	chr7	99697100	99700700	3600	161.19	3.25	1.04×10^{-13}	3.53×10^{-9}
BBC3	chr19	47733000	47736300	3300	165.46	3.33	6.89×10^{-13}	1.40×10^{-8}

Table S4

Summary of genes identified from A549 ChIP-seq data with significant clinical relevance to BTNL9 expression in lung cancer patients. (Spearman's Correlation coefficient $r \leq -0.45$ or $r \geq 0.45$). (This is a data file in Excel spreadsheet.)

Table S5

BTNL9 binding site was identified in 594 gene encoded transcription factor. (This is a data file in Excel spreadsheet.)

Table S6

List of 444 genes from A549 RNA-seq genes that fit the criteria of $\log_2$ FC ± 0.5 and FDR $q < 0.1$. (This is a data file in Excel spreadsheet.)

Table S7

Three main gene clusters regulated by BTNL9 as identified by RNA-seq analysis

DNA replication

Gene	Log_2 FC	*p*-vals
CCNA2	-0.821	9.37×10^{-8}
CDC45	-0.533	9.64×10^{-3}
CDC6	-0.553	6.34×10^{-4}
CDK2	-0.600	6.88×10^{-8}
CDT1	-1.065	1.28×10^{-10}
DBF4	-0.630	5.79×10^{-6}
DNA2	-0.602	2.65×10^{-3}
E2F1	-0.522	3.06×10^{-3}
FEN1	-0.763	7.24×10^{-9}
GINS1	-0.623	1.88×10^{-5}
MCM10	-0.795	3.15×10^{-7}
MCM2	-0.736	2.98×10^{-8}
MCM3	-0.644	1.69×10^{-6}
MCM4	-0.698	8.50×10^{-12}
MCM5	-0.836	3.48×10^{-7}
MCM6	-0.635	7.73×10^{-6}
MCM7	-0.929	5.76×10^{-22}
MCM8	-0.631	1.22×10^{-5}
ORC6	-0.502	4.38×10^{-3}
POLA2	-0.541	6.50×10^{-3}
POLD3	-0.545	3.37×10^{-4}
POLE	-0.542	3.89×10^{-3}
PRIM1	-0.654	1.06×10^{-4}
RFC5	-0.539	7.35×10^{-4}
RPA3	-0.663	5.82×10^{-7}
UBE2C	-0.729	1.11×10^{-12}

Transcription regulation mediated by p53

Gene	Log_2 FC	*p*-vals
AURKA	-0.756	2.06×10^{-16}
AURKB	-0.822	2.69×10^{-12}
BBC3	0.594	5.11×10^{-9}
BIRC5	-0.775	3.42×10^{-15}
BLM	-0.562	1.85×10^{-3}
BRCA1	-0.699	2.18×10^{-7}
BRIP1	-0.640	7.24×10^{-4}
BTG2	0.789	5.61×10^{-5}
CCNA2	-0.821	9.37×10^{-8}
CCNB1	-0.563	1.96×10^{-7}
CDC25C	-0.968	3.90×10^{-8}
CDK1	-0.618	2.46×10^{-9}
CDK2	-0.600	6.88×10^{-8}
CDKN1A	1.301	0.00
DNA2	-0.602	2.65×10^{-3}
E2F1	-0.522	3.06×10^{-3}
EXO1	-0.518	9.79×10^{-3}
FANCD2	-0.633	1.18×10^{-5}
FANCI	-0.677	2.68×10^{-7}
FOS	-0.662	7.48×10^{-6}
GADD45A	1.134	6.23×10^{-20}
JUN	0.792	7.49×10^{-13}
MDC1	-0.510	7.26×10^{-3}
MDM2	1.107	1.01×10^{-35}
PLK2	0.631	1.81×10^{-7}
PLK3	0.728	4.15×10^{-4}
RFC5	-0.539	7.35×10^{-4}
RMI2	-0.737	4.07×10^{-5}
RRAGD	0.562	4.96×10^{-3}
RRM2B	0.704	1.59×10^{-6}
SESN1	0.586	1.44×10^{-3}
SESN2	0.720	1.19×10^{-7}
TIGAR	0.863	2.45×10^{-8}
TP53I3	0.519	8.17×10^{-5}
TP53INP1	0.669	8.40×10^{-4}
TPX2	-0.739	3.79×10^{-19}

Table S7 (continue)

Cell cycle

Gene	Log_2 FC	*p*-vals
AAAS	-0.544	5.94×10^{-4}
AURKA	-0.756	2.06×10^{-16}
AURKB	-0.822	2.69×10^{-12}
BIRC5	-0.775	3.42×10^{-15}
BLM	-0.562	1.85×10^{-3}
BRIP1	-0.640	7.24×10^{-4}
BRCA1	-0.699	2.18×10^{-7}
BRCA2	-0.561	3.00×10^{-4}
BUB1	-0.797	1.24×10^{-8}
BUB1B	-0.525	2.06×10^{-4}
CASC5	-0.652	2.09×10^{-5}
CKS1B	-0.837	2.89×10^{-27}
CDC20	-0.545	9.99×10^{-5}
CDC25B	-0.536	4.96×10^{-10}
CDC25C	-0.968	3.90×10^{-8}
CDC45	-0.533	9.64×10^{-3}
CDC6	-0.553	6.34×10^{-4}
CDCA5	-0.771	5.11×10^{-5}
CDCA8	-0.824	6.63×10^{-9}
CENPA	-0.874	2.34×10^{-6}
CENPE	-0.800	2.77×10^{-17}
CENPF	-0.868	0.00
CENPH	-0.700	1.62×10^{-4}
CENPK	-0.755	2.49×10^{-6}
CENPM	-0.598	1.71×10^{-3}
CENPN	-0.727	6.14×10^{-7}
CENPU	-0.525	2.74×10^{-3}
CENPW	-0.568	8.61×10^{-4}
CEP152	-0.535	1.20×10^{-4}
CDT1	-1.065	1.28×10^{-10}
CLSPN	-0.771	8.76×10^{-8}
CCNA2	-0.821	9.37×10^{-8}
CCNB1	-0.563	1.96×10^{-7}
CCNB2	-0.967	2.91×10^{-15}
CCND1	0.629	1.25×10^{-16}
CDK1	-0.618	2.46×10^{-9}
CDK2	-0.600	6.88×10^{-8}
CDKN1A	1.301	0.00
CDKN2C	-0.883	2.14×10^{-6}
DBF4	-0.630	5.79×10^{-6}

Gene	Log_2 FC	*p*-vals
DHFR	-0.722	8.46×10^{-5}
DNA2	-0.602	2.65×10^{-3}
DSN1	-0.603	6.84×10^{-6}
E2F1	-0.522	3.06×10^{-3}
ESCO2	-0.947	1.23×10^{-9}
EXO1	-0.518	9.79×10^{-3}
FBXO5	-0.737	1.66×10^{-5}
FEN1	-0.763	7.24×10^{-9}
FOXM1	-0.727	2.81×10^{-5}
GTSE1	-0.607	7.47×10^{-5}
GINS1	-0.623	1.88×10^{-5}
H2AFV	-0.549	2.94×10^{-15}
H2AFZ	-0.752	2.93×10^{-35}
HIST1H2BK	0.582	6.94×10^{-6}
HIST1H4C	-0.800	1.40×10^{-44}
HIST2H2AC	-0.529	5.20×10^{-3}
HJURP	-0.799	6.45×10^{-16}
HMMR	-0.777	1.47×10^{-12}
ITGB3BP	-0.931	5.78×10^{-13}
KIF18A	-0.625	4.82×10^{-4}
KIF20A	-0.826	7.39×10^{-8}
KIF23	-0.835	2.20×10^{-11}
KIF2C	-0.782	6.48×10^{-7}
KNTC1	-0.728	8.40×10^{-5}
LMNB1	-0.693	1.83×10^{-9}
MAD2L1	-0.718	7.07×10^{-5}
MDM2	1.107	1.01×10^{-35}
MDC1	-0.510	7.26×10^{-3}
MCM10	-0.795	3.15×10^{-7}
MCM8	-0.631	1.22×10^{-5}
MCM2	-0.736	2.98×10^{-8}
MCM3	-0.644	1.69×10^{-6}
MCM4	-0.698	8.50×10^{-12}
MCM5	-0.836	3.48×10^{-7}
MCM6	-0.635	7.73×10^{-6}
MCM7	-0.929	5.76×10^{-22}
MIS18BP1	-0.771	1.10×10^{-11}
NDC80	-0.596	1.61×10^{-3}
NEK2	-0.738	1.17×10^{-6}
NCAPD2	-0.699	2.40×10^{-12}

Gene	Log_2 FC	*p*-vals
NCAPG	-0.743	1.92×10^{-7}
NCAPH	-0.731	4.55×10^{-6}
NCAPD3	-0.650	6.38×10^{-4}
NCAPG2	-0.705	4.95×10^{-7}
NUF2	-0.913	1.05×10^{-12}
OIP5	-0.929	5.39×10^{-8}
ORC6	-0.502	4.38×10^{-3}
PTTG1	-0.865	3.27×10^{-28}
PLK1	-0.846	3.77×10^{-18}
POLA2	-0.541	6.50×10^{-3}
POLE	-0.542	3.89×10^{-3}
POLD3	-0.545	3.37×10^{-4}
PRIM1	-0.654	1.06×10^{-4}
PRKAR2B	-0.502	1.46×10^{-2}
PKMYT1	-0.786	1.30×10^{-5}
RAD21	-0.581	7.42×10^{-15}
RHNO1	-0.540	9.91×10^{-4}
RMI2	-0.737	4.07×10^{-5}
RFC5	-0.539	7.35×10^{-4}
RPA3	-0.663	5.82×10^{-7}
RRM2	-0.734	3.26×10^{-13}
SUN2	-0.502	3.15×10^{-3}
SGOL1	-0.713	2.31×10^{-6}
SGOL2	-0.699	3.41×10^{-9}
SPC25	-0.577	1.53×10^{-3}
SKA1	-0.615	1.86×10^{-3}
SMC2	-0.781	2.05×10^{-17}
SMC4	-0.680	9.51×10^{-29}
TK1	-1.089	1.95×10^{-12}
TMPO	-0.556	6.64×10^{-10}
TOP2A	-0.867	0.00
TPX2	-0.739	3.79×10^{-19}
TUBA1A	0.735	2.32×10^{-8}
TUBA1B	-0.510	5.00×10^{-25}
UBE2C	-0.729	1.11×10^{-12}
MYBL2	-0.550	8.00×10^{-6}
VRK1	-0.502	1.32×10^{-3}
WHSC1	-0.617	1.91×10^{-9}
ZWINT	-0.713	1.53×10^{-6}

Table S8

Overlapping genes between 9,709 genes from ChIP-seq analysis and 444 DEGs from RNA-seq in A549 cells. (This is a data file in Excel spreadsheet.)

Table S9
Primers list

RT-qPCR

Primer name	Sequence (5' to 3')	Amplicon size
AURKA-F	cttcttggatcagctggagagc	170 bp
AURKA-R	caaagaactccaaggctccaga	
AURKB-F	agtgggacacccgacatcttaa	158 bp
AURKB-R	tctatctgggacttgaagaggacc	
BBC3-F	acgacctcaacgcacagtacga	147 bp
BBC3-R	cctaattgggctccatctcggg	
BTNL9-Opti-F	ccattcagaacctgctgctgagc	114 bp
BTNL9-Opti-R	gcagtggcagtgtagcaacaaagg	
CCNA2-F	gaatatcaacccggaaaaggcag	171 bp
CCNA2-R	ggaacggtgacatgctcatcat	
CCNB1-F	caaagtcagtgaacaactgcagg	174 bp
CCNB1-R	tcaggttctggctcaggttct	
CDC25C-F	agaagcccatcgtccctttgga	133 bp
CDC25C-R	gcaggatactggttcagagacc	
CDK1-F	ggaaaccaggaagcctagcatc	124 bp
CDK1-R	ggatgattcagtgccattttgcc	
CDK2-F	cggagttgtgtacaaagccaga	173 bp
CDK2-R	ctgtgtgaatgacatccagcagc	
E2F1-F	ggacctggaaactgaccatcag	121 bp
E2F1-R	cagtgaggtctcatagcgtgac	
FOXM1-F	tctgccaatggcaaggtctcct	145 bp
FOXM1-R	ctggattcggtcgtttctgctg	
GADD45A-F	ctggaggaagtgctcagcaaag	146 bp
GADD45A-R	agagccacatctctgtcgtcgt	
MCM2-F	tgccagcattgctccttccatc	163 bp
MCM2-R	aaactgcgacttcgctgtgcca	
MCM3-F	cgagacctagaaaatggcagcc	107 bp
MCM3-R	gcagtgcaaagcacataccgca	
MCM7-F	gccaagtctcagctcctgtcat	136 bp
MCM7-R	cctctaaggtcagttctccactc	
MDM2-F	tgtttggcgtgccaagcttctc	113 bp
MDM2-R	cacagatgtacctgagtccgatg	
MYBL2-F	caccagaaacgagcctgcctta	130 bp
MYBL2-R	ctcaggtcacaccaagcatcag	
ORC6-F	ccagacacagcaagtggatcttg	125bp
ORC6-R	ttacaccggatgtggctaccat	
GAPDH-F	acatcgctcagacaccatg	143 bp
GAPDH-R	tgtagttgaggtcaatgaaggg	

Table S9 (continue)

ChIP-qPCR

Primer name	Sequence (5' to 3')	Amplicon size
Chp53-F	GACTGACAGCCTTCAATTGGATT	176
Chp53-R	CATGGCATCCTAGTGAAAACTGG	
ChCDKN1A-F	GACATTTGACAACCAGCCCTTTG	122
ChCDKN1A-R	GATTGTGGCTAAACCCCAGAAAG	
ChE2F1-F	AAAGTCCCGGCCACTTTTACG	135
ChE2F1-R	CCGGCGCGTTAAAGCCAATA	
ChMCM7-F	CTGGTAAAGGTGTTAGGGTCGTG	160
ChMCM7-R	GAACCTGGGAATGCCCAAAAG	
ChBBC3-F	AGACTCGGAGGAACTGGAGAA	165
ChBBC3-R	TCAGATTCCAGGAGGGACTTCC	

Plasmid construction

Primer name	Sequence (5' to 3')	Amplicon size	Remark
BTNL9-WT-F	GTCAGATCCGCTAGCGTCGCCACCATGGTCGAT	1664	
BTNL9-WT-R	TGTCTCGAGGTCGAGAATTCTACCACCAGTCCAGAGCAGGATCA		
BTNL9-AmTag-F	GTCAGATCCGCTAGCGTCGCCACCATGGTCGATCTGAG	1782	
BTNL9-AmTag-R	TCGAGGTCGAGAATTCTAGTAAGCCTGAGAGAGTATCACG		
BTNL9-Flag-R	TCGAGGTCGAGAATTCTACTTGTCATCGTCATCCTTGTAGTCGATGTCATGATCTTTATAATCACCGTCATGGTCTTTGTAGTCaCTACCACCTCCGCCAGATCCACCGCCTCCgtgCCACCAGTCCAG	1746	Paired with BTNL9-AmTag-R
BTNL9-ΔbZIP-IntR	AGCCCGCCATTCAGCCTGCTTTCTCAGCACTCCCAGAG		Paired with BTNL9-AmTag-F
BTNL9-ΔbZIP-IntF	GTGCTGAGAAAGCAGGCTGAATGGCGGGCTGC		Paired with BTNL9-AmTag-R
BTNL9-MutbZIP-IntR	TCTGCTTCTCCGCTGCTTTCTCAGCACTCCCAGAGCagg		Paired with BTNL9-AmTag-F
BTNL9-MutbZIP-IntF	AAAGCAGCGGAGAAGCAGAccgAAGccaCGGAAGCAGGCCccaAAGCGGCAAccgAAACTGACCGCCcctccgGAAAAAcct		Paired with BTNL9-AmTag-R

DNA-protein pulldown template

Primer name	Sequence (5' to 3')	Amplicon size
DP-E2F1-F	CTATAGAAAGGTCAGTGGGATGCG	235
DP-E2F1-Rbiotin	GGTTCCTATTGGCTTTAACGCGC	

CRIPSR-cas9 construction

Primer name	Sequance (5' to 3')
TP53-F	CACCGTCCACTCGGATAAGATGCTG
TP53-R	AAACCAGCATCTTATCCGAGTGGAC

Table S10
Antibodies list

Antibody	Cat number	Source	Identifier
Am-Tag	91111	Active Motif	RRID:AB_2793779
BBC3	55120-1-AP	Proteintech	RRID:AB_10859944
BTNL9	LS-C399149	LS-Bio	
CASP9	9502S	Cell Signaling	RRID:AB_2068621
CDC25C	16485-1-AP	Proteintech	RRID:AB_2291330
CDK1	19532-1-AP	Proteintech	RRID:AB_10638617
CDK2	A0094	Abclonal	RRID:AB_2861449
CCNA2	A19036	Abclonal	RRID:AB_2862528
CCNB1	A19037	Abclonal	RRID:AB_2862529
CDKN1A	2947T	Cell Signaling	RRID:AB_823586
E2F1	32-1400	Thermo Fisher Scientific	RRID:AB_2533065
FOXM1	13147-1-AP	Proteintech	RRID:AB_2106213
GADD45A	A11768	Abclonal	RRID:AB_2758745
H3K27ac	MA5-23516	Thermo Fisher Scientific	RRID:AB_2608307
MCM2	10513-1-AP	Proteintech	RRID:AB_2142131
MCM3	15597-1-AP	Proteintech	RRID:AB_2141973
MCM7	11225-1-AP	Proteintech	RRID:AB_2297584
MDM2	66511-1-Ig	Proteintech	RRID:AB_2881874
MYBL2	18896-1-AP	Proteintech	RRID:AB_10644283
ORC6	17784-1-AP	Proteintech	RRID:AB_2283320
TP53	2527	Cell Signaling	RRID:AB_10695803
YY1	63227	Cell Signaling	RRID:AB_2799641
β-Actin	4970	Cell Signaling	RRID:AB_2223172
Goat anti-mouse HRP	115-035-003	Jackson Immunoresearch	RRID:AB_10015289
Goat anti-rabbit HRP	111-035-003	Jackson Immunoresearch	RRID:AB_2313567
Goat anti-rabbit IgG Alexa Fluor® 594-conjugated	A32740	Thermo Fisher Scientific	RRID:AB_2762824
Mouse IgG2a Isotype Control	02-6200	Thermo Fisher Scientific	RRID:AB_2532943
